# Is the Illegal Trade of Glass Eels (*Anguilla anguilla*) Increasing the Spread of Disease? A Case of EVEX

**DOI:** 10.3390/microorganisms10112208

**Published:** 2022-11-08

**Authors:** Ivana Giovanna Zupičić, Dražen Oraić, Željko Pavlinec, Dinko Novosel, Luka Žuvić, Tanja Šegvić-Bubić, Snježana Zrnčić

**Affiliations:** 1 Laboratory for Fish Pathology, Croatian Veterinary Institute, 10000 Zagreb, Croatia; 2 Department of Ornithology, Croatian Academy of Sciences and Arts, 10000 Zagreb, Croatia; 3 Laboratory for General Pathology, Croatian Veterinary Institute, 10000 Zagreb, Croatia; 4 Laboratory for Aquaculture, Institute of Oceanography and Fisheries, 21000 Split, Croatia

**Keywords:** European eel (*Anguilla Anguilla*), illegal trade, EVEX virus, phylogenetic analyses, critically endangered species

## Abstract

The European eel (*Anguilla anguilla*) is a catadromous species that inhabits the rivers of the Adriatic watershed in Croatia. It is a critically endangered fish species, according to the IUCN, due to its declining abundance in European rivers caused by overfishing and trafficking and by diseases caused by nematodes, pathogenic bacteria and viruses. An illegal parcel of glass eels was confiscated at the Zagreb Airport and was intended to re-populate Croatian rivers. Barcoding was employed to determine species affiliation, and a thorough health check was carried out. This study reports the evaluation of gross lesions, histological findings, and EVEX virus isolation and identification. Since the confiscated glass eels were of unknown origin and given the serological and genetic similarities of EVA and EVEX, we designed primers and probes for almost whole genome sequencing to elucidate the origin of glass eels based on viral phylogeny. Bayesian phylogeny showed that the isolated strain had the most common ancestor with a Danish isolate and likely evolved from the French isolate of EVEX. These findings are discussed in light of the divergence of recently isolated strains and their possible contribution to the decrease of the abundance of the European eel in European waters.

## 1. Introduction

The European eel (*Anguilla anguilla*) is a catadromous species [1] that inhabit the rivers of the Adriatic watershed in Croatia. Due to the general decline of its abundance in the European rivers, it has been classified as a globally critically endangered fish species according to the International Union for Conservation of Nature (IUCN) Red List, facing an extremely high risk of extinction in the wild [2]. Similarly, the surveillance of the length–weight relationship (LWR) of this species in Croatian rivers has shown a notable decline [3]. The main threats to its survival in European rivers are overfishing and trafficking [4], in addition to diseases like swim bladder nematode *Anguillicoloides crassus* [5], pathogenic bacteria [6] and pathogenic viruses such as the eel virus European (EVE), the eel virus European X (EVEX), anguillid herpesvirus (AnHV) and the eel picornavirus 1 (EPV-1) [7,8,9].

European eel (*A. anguilla*) and Japanese eel (*Anguilla japonica*) are traditional culinary delicacies consumed primarily in Asia and, to a lesser extent, in European countries [10]. Although some progress in the artificial reproduction of eels has been achieved [11], it is not yet possible on a commercial scale. Therefore, all market demands are supplied on catches of wild glass eels or elvers for aquaculture production and conservation [12]. In Europe, *A. anguilla* is produced in intensive recirculation systems with a regulated water temperature [8].

In recent decades, many disease outbreaks in eels have been reported, with new viruses isolated and proven to be the causative agents [13,14,15]. Occasionally, viruses were isolated from clinically healthy eels [16,17,18,19,20,21] and from those with disease symptoms, and double infections with different viruses have also been described [1,9,21,22,23].

The first report of eel virus European X (EVEX) from the genus *Vesiculovirus* (family *Rhabdovirus,* order *Mononegavirales)* in Europe dates back to the early 1980s [16,18,24]. It is widespread in both wild and farmed European eel populations, and it has been detected in eels originating from Germany [22], Denmark, the United Kingdom, Sweden, the Netherlands, France [16,18,25] and Italy [1]. Although several authors have isolated EVEX from apparently healthy *A. anguilla* elvers [16,18,20], the infection may result in severe haemorrhagic disease with significant mortalities [20,26]. However, rhabdovirus EVEX was described as serologically, morphologically and genetically related to eel virus American (EVA) described by van Beurden [27].

In early 2019, an illegal parcel containing 252,000 glass eels was confiscated at the Zagreb Airport. Eels were placed into the tanks of the city zoo and earmarked for the re-population of natural eel habitats in Croatian karst rivers. Therefore, an extensive health check was undertaken. The present study reports the results of the health status evaluation describing gross lesions, histological findings, EVEX virus isolation and identification. Since the origin of the confiscated glass eels was not known, primers and probes for almost whole genome sequencing of the isolated virus were designed to elucidate their origin based on viral phylogeny.

## 2. Materials and Methods

The confiscated glass eels were transferred to the city zoo and placed in several fibreglass tanks with a capacity between 200 and 3500 L and supplied with continuously aerated tap water. The water in the tanks was replaced daily. Aquarium heaters were placed in each tank, and the water temperature was maintained between 15 and 20 °C. Fish were fed frozen roe, and later, the frozen roe was mixed into commercial pellets. Dead fish were removed daily.

### 2.1. Gross Pathology, Parasitological, Bacteriological and Histopathological Examination

A sample of 50 glass eels was submitted for health control and species determination to Croatian Veterinary Institute, Laboratory for Fish Pathology Animals were sacrificed by immersion in MS-222 solution (Pharmaq, Overhalla, Norway), and caudal peduncles of five animals were preserved in absolute ethanol and sent to the Institute of Oceanography and Fisheries, Laboratory for Aquaculture for species identification. The submitted glass eels were necropsied, and samples were prepared for parasitological, bacteriological, virological and molecular analysis. Skin and gill scrapings of 30 animals were checked for ectoparasites. Internal organs were compressed between two slides and checked for the presence of endoparasites. Visceral organs were collected, and three pooled samples consisting of the visceral organs of ten fish were prepared for virological examination.

Swabs of skin and internal organs were plated onto Blood agar (BA, Oxoid, Hampshire, UK) and Tryptone Soy Agar (TSA, Merck, Kenilworth, NJ, USA) and incubated at 22 °C for 72 h.

The anterior parts of ten specimens were fixed in 10% neutral buffered formalin. Formalin-fixed tissues for histological examination were dehydrated through a graded series of ethanol, succeeded by xylene, embedded in paraffin, sectioned at 5 μm, and mounted on Microme EC 350-2 slides (Thermo Scientific, Waltham, MA, USA). Mounted slides were heated to 60 °C, deparaffinised and rehydrated in xylene, a graded series of alcohol and finally water, followed by staining with hematoxylin and eosin (H&E).

### 2.2. Ethical Approval

The glass eels were submitted by the Fund for Environmental Protection and Energy Efficiency, Ministry of Economy and Sustainable Development. Since the animals were not used for experiments but for diagnostic purposes, the sacrifice of animals was conducted in compliance with the principles of good veterinary practice and in full respect of animal welfare as stipulated in the Act on Animal Protection in Research Purposes, Chapter 1, Article 5. Fish, Annex IV, Table 3 [28]. The Ethics Committee of the Croatian Veterinary Institute decided that no formal approval was required, and the study was in accordance with the national legislation.

### 2.3. DNA Extraction and Amplification for Barcoding

Five specimens of glass eel were chosen for species identification using a DNA barcoding approach. DNA was extracted from caudal peduncle tissue using the DNeasy 96 Tissue Kit (Qiagen, Germany) following the manufacturer’s protocol. A partial fragment of the mitochondrial cytochrome c oxidase subunit I (COI) was amplified following Ward et al. [29]. PCR product sequencing was performed by Macrogen (Amsterdam, The Netherlands). BLAST (NCBI, available online) was used for sequence identification. Sequence alignment was run by the ClustalW tool [30], while phylogenetic analysis was carried out using the Maximum likelihood (ML) approach in Mega v6 software [31].

### 2.4. Virological Examination and Virus Identification

The three pooled samples of visceral organs of ten fish were homogenised by mortar and pestle with sterile sand and suspended 1:10 in EMEM (Euro Clone, Pero, Italy) supplemented with 10% *v*/*v* foetal calf serum (Biological Industries, Cromwell, CT, USA) and 2% *v*/*v* antibiotic-antimycotic solution (penicillin 100 UI/mL, streptomycin sulphate 10 mg/mL, amphotericin B 25 μg/mL and kanamycin 10 mg/mL) (Gibco, Waltham, MA, USA). Samples were centrifuged at 2500× *g* for 20 min at 4 °C. Supernatants in dilutions of 1:10 and 1:100 were inoculated onto one-day-old epithelioma papulosum cyprini (EPC) [32] and bluegill fry (BF-2) [33] cell monolayers grown in 96-well cell culture plates (Sigma-Aldrich Nunc, Denmark). Each pooled sample was inoculated on 24 wells; 12 wells were used as negative control and 12 wells were inoculated with viruses used as positive controls both on EPC and BF2 cell lines. The plates were incubated at 15 °C for 7 days. The supernatants from wells showing a cytopathic effect (CPE) were used for virus identification. After CPE was detected, a 25 mL flask containing 24-h-old BF2 cell lines was inoculated with 15 µL supernatant to propagate the virus for further studies.

#### 2.4.1. ELISA for Detection of Infectious Pancreatic Necrosis Virus (IPNV)

Supernatants collected from the wells in which CPE was observed were analysed for the presence of IPNV using a commercial ELISA kit (Test-line Ltd., Brno, Czech Republic) according to the manufacturer’s instructions.

#### 2.4.2. DNA/RNA Extraction

Nucleic acid was extracted from both tissue homogenate and supernatant from cell lines with an observed cytopathic effect (CPE). The flask containing BF2cell lines where 75% of the surface showed CPE development was frozen at −80 °C overnight, thawed, centrifuged, and the supernatant was filtered through a membrane filter (0.45 μm; Millipore, Germany), aliquoted and refrozen at −80 °C. DNA extraction and purification were performed from 200 µL filtrate and 200 µL tissue homogenate using a MagMAX CORE Nucleic Acid Purification Kit for rapid purification of high-quality DNA and RNA for downstream molecular analysis (Applied Biosystems, Waltham, MA, USA) according to the manufacturer’s protocol on a KingFisher Duo Prime Purification System (Thermo Scientific, Waltham, MA, USA).

#### 2.4.3. PCR Testing for the Presence of AnHV

Extracted DNA was used for endpoint PCR aiming to detect the presence of AnHV according to the procedure described by Rijsewijk et al. [34]. The reaction mix was prepared using 10 µL HotStarTaq Master Mix (Qiagen, Hilden, Germany), 2 µL purified nucleic acid, 0.4 µM primers developed by Rijsewijk et al. [34] and nuclease-free water to a final volume of 20 µL. Amplification was performed in a ProFlex PCR System (Applied Biosystems, Waltham, MA, USA), using the following thermal profile: 15 min at 95 °C for polymerase activation, followed by 40 cycles of denaturation, annealing and extension as follows: 30 s at 94 °C, 45 s at 65 °C, 1 min at 72 °C, and final extension 10 min at 72 °C. Electrophoresis was performed in the QIAxcel system using the QIAxcel DNA Screening Kit (Qiagen, Hilden, Germany).

#### 2.4.4. Real-Time RT-PCR for the Presence of EVE and EVEX

For the detection of EVE and EVEX, we used Taqman real-time PCR (rPCR) on the Rotor-Gene Q system (Qiagen, Hilden, Germany) using the QIAGEN OneStep RT-PCR kit according to the manufacturer’s instructions. For EVE we used primers and probes developed by Janssen [35], and for EVEX, primers and probes developed by [27].

### 2.5. Sequencing and Phylogeny of EVEX

#### 2.5.1. Primer and Probe Design

Since the origin of glass eels was unknown, we designed new primers that enabled a comprehensive phylogenetic comparison of EVEX strains of European origin and eel virus America (EVA) strains originating from Cuba. EVEX genome sequences available in the Nucleotide database (*Nucleotide [Internet]*, 2019), listed in Supplementary Materials, were aligned in Geneious 11.0.5 using MAFFT v7.388 [36]. We used all available EVEX and EVA sequences that included N, P, C, M and G genes. From this alignment, we designed four pairs of primers to amplify the region covering the N, P, C, M and G genes (Table 1).

Amplification was carried out using SuperScript III One-Step RT-PCR System with Platinum Taq DNA Polymerase (Invitrogen; Waltham, MA, USA). The thermal profile was as follows: reverse transcription for 20 min at 50 °C, followed by 40 cycles of denaturation for 15 s at 94 °C, 30 s at annealing temperature (Table 1) and 2 min of elongation at 68 °C, followed by final elongation at 68 °C for 5 min. Amplified fragments were purified using Illustra ExoProStar (Merck; Germany) according to the manufacturer’s instructions. Sanger sequencing was performed by Macrogen Europe (Amsterdam; The Netherlands). Obtained sequences were aligned and assembled in one contig using Geneious Prime 2019.2.1 software. For the phylogenetic analysis, we compared our sequence with the sequences listed in Supplementary Materials.

#### 2.5.2. Bayesian Phylogeny

Sequences were split into partitions where each partition was one ORF/gene: gene N 1-1382, gene P 1491-2471, gene C 1959-2156, gene M 2476-3365, gene G 3368-5242 and gene L 5249-11747. Complete sequences were aligned using the ClustalW program in MEGA 6 software, each partition/gene was aligned using the algorithm for codons, and all stop codons were removed from sequences [30]. Complete genome sequences and partition sequences were generated to the nexus file in MEGA6 software. Bayesian phylogeny was calculated using the BEAST v1.10.3 software package. The program was run with Markov Chain Monte Carlo 800,000,000 states in length until effective sample size values were over 200. The calculation was performed using four different models:

1. substitution model General time reversible (GTR) [37], invariant sites were set to be estimated with four categories of gamma-distributed rate heterogeneity and a proportion of invariant sites GTR (GTR + Г4 + I), for the complete sequence. For partitions codon models Shapiro–Rambault–Drummond 2006 (SRD06) [38] uncorrelated relaxed lognormal [39], Coalescent Bayesian Skyline model;
2. for complete sequence, same as above. Shapiro–Rambault–Drummond 2006 (SRD06) [38] for genes N, M, G and L while for overlapping genes P and C substitution model GTR [37], invariant sites were set to be estimated or to be empirical with four categories of gamma-distributed rate heterogeneity and a proportion of invariant sites GTR (GTR + Г4 + I). We used uncorrelated relaxed lognormal [39] and the Coalescent Bayesian Skyline model;
3. for complete sequence, same as above. For all partitions, codon models Yang 1996 (YANG96) [40] we used the uncorrelated relaxed lognormal Coalescent Bayesian Skyline model;
4. for complete sequence, same as above. For all partitions, codon models SRD06 [38] we used a strict molecular clock [39] and a Coalescent Constant size model.

To compare different phylogenetic models, BEAST log files were analysed in Tracer v1.6 to calculate the Akaike information criterion for Markov chain Monte Carlo (AICM) parameters and which model has a better fit [41,42]. The selected tree file was compiled in TreeAnnotator v2.4.7 from the BEAST package, while clade credibility bars were calculated when Posterior probability (PP) was higher than PP > 0.95, which was considered statistically significant. The Most common ancestor (MCA) tree was constructed in FigTree v1.4.3 [43].

## 3. Results

Nearly half of the population transferred to the city zoo tanks died within three days. The affected fish showed lethargy and ataxia. It was observed that the conversion of feed was better when the water temperature was between 18–20 °C. During the time in the tanks, the glass eel started to metamorphose into elvers.

### 3.1. Gross Pathology, Parasitological and Bacteriological Examination

**Figure 1:**
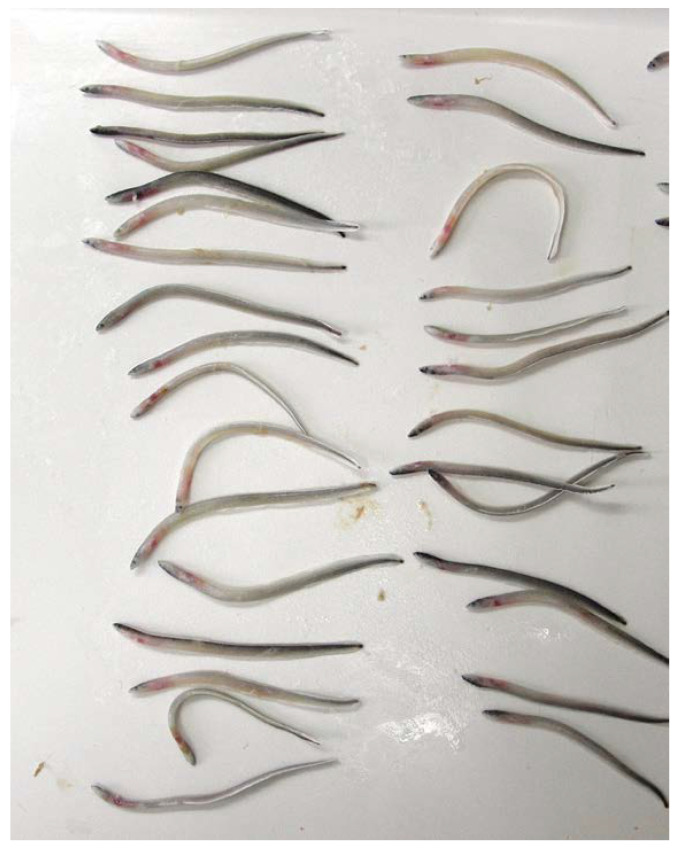
Haemorrhages on the skin of European glass eel.

**Figure 2:**
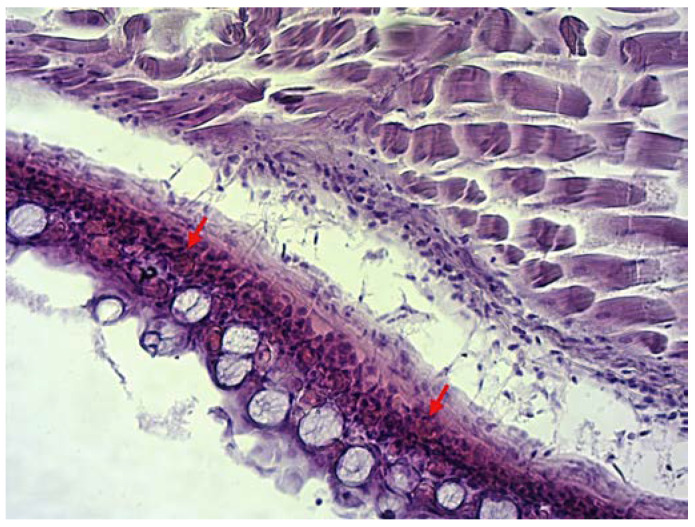
Haemorrhages in subcutaneous tissue. Figure showing section through the subcutis and musculature tissue. Arrows show area of haemorrhages. (H&E, magnification 20×).

Specimens submitted for health control were 60 to 70 mm in length. During the clinical examination, extensive haemorrhages were noticed on the opercula and around the pectoral and ventral fins (Figure 1). Parasitological and bacteriological examinations tested negative. Results of the histological analysis in infected animals disclosed haemolysis and dilated capillaries on the gills and light to moderate haemorrhages in subcutaneous tissue (Figure 2) with massive intravascular haemolysis.

### 3.2. Barcoding

**Figure 3:**
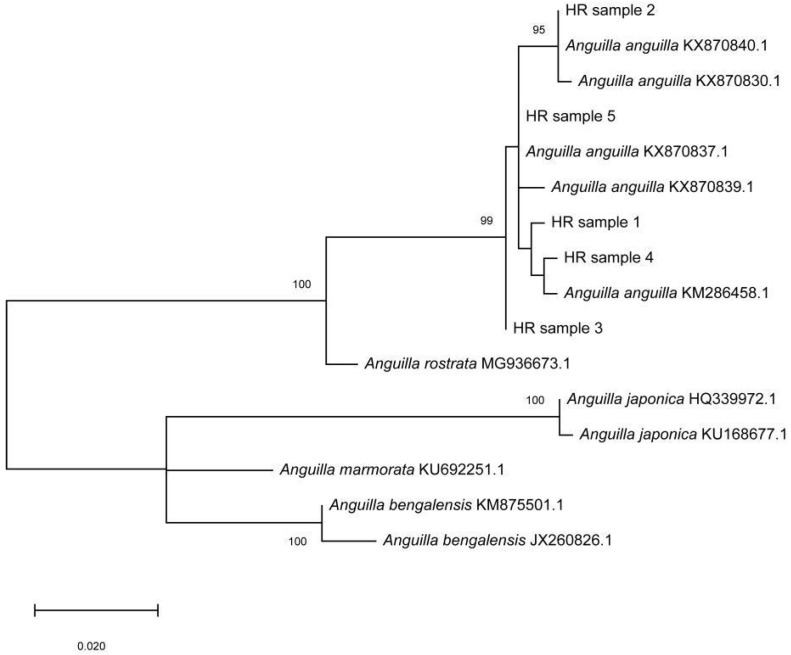
Unrooted Maximum-likelihood (ML) phylogenetic tree of the genus *Anguilla* inferred from the cytochrome b coding (COI) region of mtDNA. HR samples 1–5 were the individuals studied. COI sequence data are labelled with their GenBank accession numbers. Numbers at nodes are bootstrap percentages (>90%) after 1000 replicates based on distance. The scale bar represents an interval of the Kimura two-parameter (K2P) model.

Genetic analysis undoubtedly identified the eel specimens as the European eel *Anguilla anguilla.* Namely, 559 bp long mtDNA COI fragments of the studied specimens produced significant alignments with sequences of the European eel from the Genbank with 99–100% similarity. In addition, phylogenetic reconstruction segregated the tested samples (HR samples 1–5) and *A. anguilla* samples from other *Anguillidae* species into a separate clade (Figure 3). The obtained COI sequences were deposited in GenBank under the accession numbers OP493206-OP493210.

### 3.3. Virological Examination and Identification

All three pooled samples of homogenised organs inoculated onto BF2 cell lines and incubated at 15 °C produced CPE after five days. Supernatant collected from the cell culture tested negative for the presence of IPNV using ELISA. Examination of tissue homogenates to rule out AnHV using endpoint PCR tested negative. Tissue homogenates and the supernatant collected from cell culture tests targeting EVE tested negative. However, both tissue homogenates and the supernatant tested positive for the EVEX.

### 3.4. Phylogenetic Analysis

**Figure 4:**
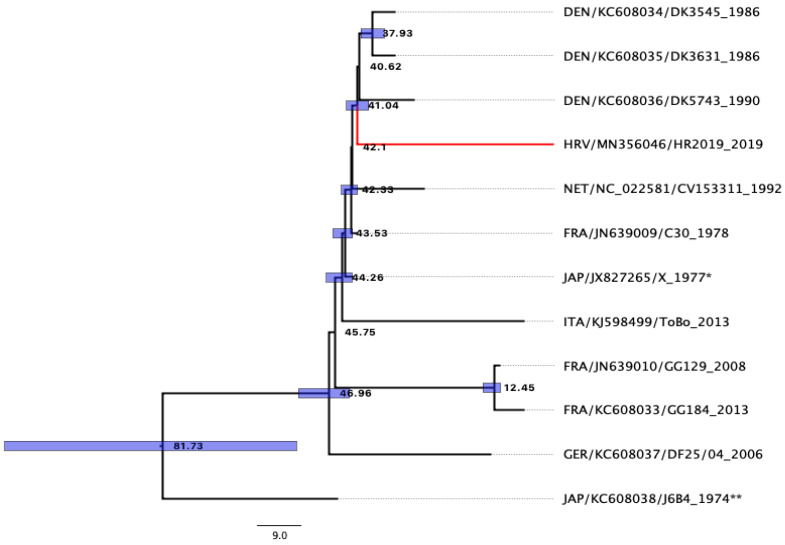
The most common ancestor (MCA) phylogenetic tree of the EVEX isolate based on the studied genes (N, P, C, M and G), constructed using Bayesian phylogenetic analysis. The numbers on the tree are equal to years. Blue boxes mark credibility interval with probability PP > 0.95. * Strain isolated in Japan from eels imported from France [14]. ** Strain isolated in Japan from eels imported from Cuba [13].

Analysis of sequences obtained using primers designed for this study confirmed that the isolated virus belongs to the eel virus European X. After assembly, a single consensus sequence of our EVEX isolate was obtained and was 5150 base pairs in length (GenBank accession number MN356046). The obtained phylogenetic tree can be seen in Figure 4.

Results obtained using Bayesian phylogeny showed that AICM was lowest when using the substitution model GTR [37], with sites set to be estimated with four categories of gamma-distributed rate heterogeneity and a proportion of invariant sites GTR (GTR + Г4 + I) for complete sequence, and partitions with codon model YANG96 [40] using an uncorrelated relaxed lognormal Coalescent Bayesian Skyline model. The most common ancestor to the Croatian strain HR2019 (accession number MN356046) was the Danish strain before 41.04 y (95% HPD 38.67–43.32 y, PP 0.98) and likely evolved from the French strain C30 (KC608036) from 1978. Taxa names consisted of the following information: “Country of origin 3digit code/sequence accession number/strain name _year of isolation”.

## 4. Discussion

The samples of confiscated eels were analysed to confirm anticipated species affiliation and to evaluate their health status. The glass eels are European eels *A. anguilla.* All obtained sequences showed high similarity (99–100%) to the sequences available in GenBank. In recent decades, populations of European eel have shown a strong decline of almost 90–99% [44] globally and have been listed as a critically endangered species [2]. As a significant decline has also occurred in Croatian open waters [3], re-population of Croatian karst rivers using the confiscated consignment would be exceptionally beneficial. Since the origin of the smuggled eels was unknown, it was essential to avoid the spread of any infectious pathogen into Croatian natural habitats, thereby further endangering the remaining population of European eels. Unfortunately, there are no studies or available data on the presence of any pathogens in populations of European eels in Croatian or in regional waters to date. For this reason, prior to any release of the confiscated glass eels into open waters, they were quarantined, and efforts were made to ensure the proper conditions for their survival until the completion of the health status analysis.

During quarantine at the city zoo, increased mortality was noted, and laboratory analysis revealed the presence of eel virus European X (EVEX), one of the three major viruses in farmed and wild European eels [8,12]. Necropsy showed haemorrhages in the skin of analysed glass eels (Figure 1) and supported the pathology described previously [20,26]. This finding was confirmed histologically (Figure 2) though other histopathological changes were not pronounced to a level that would cause mortality. Therefore, we could assume that crowding in the transportation bags and placing glass eels in zoo tanks induced stress, likely causing favourable conditions for virus multiplication and disease manifestation. A similar scenario was previously described in Atlantic salmon [45].

It has been suggested that this virus hinders the migration of spawners, which was supported by the migration simulation of silver eels experimentally infected with EVEX [26]. Haemorrhagic septicaemia and concomitant anaemia caused by EVEX hamper swimming activities and the stamina needed to reach the spawning area in the Sargasso Sea. During the last decade, EVEX has only rarely been confirmed in wild populations, and the assumption of the virus’s contribution to the decline of eels seemed to be inconsistent [12]. Nevertheless, our finding of EVEX in wild eels of unknown origin has confirmed the presence of the virus in wild populations, presumably caught in European rivers upon their return from the Sargasso Sea. The presence of the virus in wild eels was also recently confirmed in the report of mortalities of wild European eels in 2019 associated with EVEX in England [46], again raising the possibility of the hindered migration of spawners due to infectious diseases. Additionally, a thorough health study using sensitive molecular tools was conducted in 2015 on European eel populations from Lough Neagh, the largest wild-caught European eel fishery in Europe. The results showed the presence of both EVE and EVEX viruses [47] for the first time in this fishery. Moreover, clinical symptoms of the diseases were also observed. Previously, there were no records of viral diseases in this area based on clinical signs. However, it is difficult to claim that the viruses were not present in the population since they can be present in asymptomatic carriers [14,16,18,25]. In 2018, the presence of AnHV, EVEX and, for the first time, an eel picorna virus was detected in wild yellow and silver elver categories of European eels without any symptoms collected in the North Rhine Westphalia rivers in Germany [48]. Based on these reports, it can be hypothesized that the viruses were circulating in wild populations without visible disease symptoms or disease outbreaks. On the contrary, a clinical disease outbreak caused by EVEX was reported from an experimental hatchery in Italy [49] triggered by harvesting, shipping and placing elvers into tanks. Elvers originated from nature and were seeded in tanks at the size of 15–35 g, but the country of their origin was not defined. Phylogenetic analyses of the viral genome revealed divergence compared to the previously analysed EVEX isolates.

The origin of the smuggled glass eels in our study was unknown, and the main purpose of this study was to use molecular tools to determine whether they belong to the species *A. rostrata* or *A. anguilla* and to determine the strain of virus, EVA or EVEX, isolated from individuals [50]. After the detection of two very similar viruses in eels imported to Japan [13,14], Stone et al. [50] suggested that both EVA and EVEX belong to the new species of Anguillid rhabdoviruses within the genus *Perharhabdovirus*. A newly designed set of primers in this study undoubtedly confirmed the virus’s affiliation with the species *Pherhabdovirus anguilla* within the genus *Perhabdovirus* [51]. The results of Bayesian phylogenetic inference of the studied genes showed that the age of the most common ancestor of EVEX and EVA is 81.73 years (95%HPD 53.82–114.87, PP 1). Unfortunately, there have been very few reports of sequencing and phylogenetic studies of the genes analysed in this study, and we additionally attempted to compare our EVEX isolate with previously described ones. This analysis suggests that there was an explosion of different EVEX strains in Europe between 41.04 and 46.96 years ago (95%HPD 38.67–53.36), with most of the current strains, including the one detected here, evolving from and descending directly from the French strain C30 (Figure 4).

The nucleotide distances and divergence times of several EVEX strains suggest that additional divergent strains are circulating in European waters, as shown in the example of the recently isolated and analysed strain in an Italian hatchery [49]. Bellec et al. [52] studied over 50 EVEX isolates from five European countries over a period of 40 years and concluded that their evolutionary relationships were not correlated with geographical isolation, date or host life stage. However, if longer sequences of these isolates are available, it would be interesting to compare them to obtain a clearer and more accurate picture of the evolutionary history of EVEX. Most eel farming in the Netherlands, Denmark and France relies on the import of elvers from France and Portugal, and viral strains are circulating through Europe with the intense trade of eels.

EVEX is widespread in different European countries, causing either disease symptoms, as noted in two cases in the UK [46,47] and an Italian hatchery [49], or being present in the host without disease symptoms, as found in German rivers [48]. It is known that nucleotide substitutions during virus replication could be caused by aquaculture practices, as high fish densities and water temperatures increase stress on fish. In the case of rhabdoviruses [8], experimental trials suggested that mortalities are lowest at 10 °C and highest at 20 °C, which could explain the onset of mortalities in our described case.

Finally, we could conclude that the batch of confiscated, EVEX-positive eels was not appropriate for re-populating Croatian rivers. However, if the European eels inhabiting Croatian open waters are also infected with EVEX, as was the case in German rivers [48], the introduction of these individuals may not have been an additional cause of threat. To avoid losing similar opportunity of increasing their abundance in the wild, greater efforts should be invested in evaluating their health status by creating programmes aimed at preventing their extinction.

## Figures and Tables

**Table 1:** Set of primers designed for phylogenetic analysis of isolated EVEX.

	Sequence 5′–3′	Amplicon Length (bp)	Annealing Temperature
EVEX-N-F1	GGCTATTCTTTAACAGACATCTG	1559	50 °C
EVEX-N-R2	CTTTTGCCAGCAATTGTATCCC		
EVEX-P-F1	GTGAGTTGAAGCAGRAATGARTC	1223	50 °C
EVEX-P-R2	GATTCTGTYTTCTTCCCCTTC		
EVEX-M-F1	GATGCCTTRAATTGGCTTGAC	989	50 °C
EVEX-M-R2	CTCRAAAYAATSGTGTATCACTG		
EVEX-G-F1	GGTGTTCAATCTTGATTCTG	2088	46 °C
EVEX-G-R2	CTTCGTCATACATGATGACTG		

## Data Availability

All data are available upon reasonable request from the corresponding author.

## Supplementary Materials

The following supporting information can be downloaded at: https://www.mdpi.com/article/10.3390/microorganisms10112208/s1, Table S1: EVEX genome sequences available in Nucleotide database (*Nucleotide [Internet]*., 2019 used in this study. References [49,51,53,54,55] are cited in the Supplementary Materials.
