# Supplementary material for: Is the Illegal Trade of Glass Eels (Anguilla anguilla) Increasing the Spread of Disease? A Case of EVEX
Source: Microorganisms. 2022 Nov 8;10(11):2208. doi: 10.3390/microorganisms10112208 (PMC9692847; doi:10.3390/microorganisms10112208)
Supplement: Supplementary file 1 [file microorganisms-10-02208-s001.zip › microorganisms-1973437-supplementary.pdf]

Table S1: EVEX genome sequences available in Nucleotide database (*Nucleotide [Internet].*, 2019 used in the study

Accesion number	Sequence	Additional information from GenBank
JN639009	TAAGGCTATTCTTTAACAGACATCTGTTTGTTTCATTGAAAATGGCTGCCATGAACT<br>CCATCAAAGTTACAGTTAGAGCCAATGGACAAGATTTCACCCCAGTTTGTCCTAAA<br>ATGGAGGATGAAGTGGCTTACCCATCTGATTATTTAGATGCTAATGGATTACCAACC<br>TTCCAGCTCTACTATCATGATCTGTCAAAAAAGGATCTGCTAGACCTGATCTGGGGA<br>GAAGCATCAGAGGCAAGGCTGCCTAGTGAGCTGGTGACCGCGTACGTGTACAAGG<br>TGGTGAGTGAGTGGAAAGAGATGCTGGAGAGTGATTGGTCATCTTTCCGATTCCAG<br>ATTGGCAAAGCGGGAGAGGAGATAACCCCATTCAACCTGATTGGCATGACTGTGA<br>ACGGTCAGAAACTAGCTGACTACAAAAAAGCAGTTGCTCCTGAGGGGATAGATGA<br>GGTAGCAATGGTGATCTATCTCCTGGCACCATACCGGATAGTCGGAATTAAAAACG<br>AAGACTATCAAGATCGGGTGATCACCAACATCCAGAACCAGCTGGACAGTCTCGG<br>CGCAAAGAAGCTGCAGGTGAAGGCACTGAAGAATGTCACTACTCTCATCAACAGT<br>GCCAACTATCTGAAAATGGTGGCAGTGATTGACATGTTCTACTACCATTTCAAGAAC<br>AGTCAAGAGAGAGCTGTTGTCAGGATTGCCACTCTGAGTTCCCGTCACAAAGACTG<br>TGCAGCTCTATCCACCCTGAATCACATCACCAGCTTCACTGGGAGGAGTTTTGTGCA<br>GGTGTTGGATTGGGTTTTCACAGATCAAGTTGCCAAGGAGATCGGCAGGATGATGC<br>GTGCGGGTCAAGAAATTGATCGTCCTGAATCCTACATGCCGTACCTGAAGGACTTG<br>GGGCTGAGCAGAAAATCCCCCTACTCCTCCTCAGCAAATCCTGGCACTCACTGCTG<br>GGCACAGATGGTATGTGCCATGATGGGATCCAAAAGATCTCAAAACGCAATCGCC<br>AGCACTGAAGAGAATCTCAGCAATCTGACAAGAAATGCTGAGATTATGGCCTATGC<br>CCTGGGAGTGGGAGCAGACCTGGTGAAGGGACTGATCATTGGAGATCAGAAGGAA<br>GGAGACTCCGGTGTCATCCCGGATGAGGAGGGAATGGATGAGCCCAACAACATGG<br>AGGCCCAAGATTGGCTGGAGTACATGGCCTCAAAGGGATTCAAGCTGACTCCAAAT<br>ATGGAGCTGCAAGTCCGCCACATGTGCCTGCGAATCACCAATCCCAGAAAAGCCA<br>CTCTGGGGAGTTACCTGCGGGAGAGATACAGTTCATCCCTGTGAGTTGAAGCAGAA<br>ATGAGTCATTTTTAGAACTATTATATTTTTATCCTAATAGCCTTAAATAGTTAGTCAT<br>CTTGTGCTTAGATATTATGCCCTAGTTATATGAAAAAAAACTTTAACAGGGATAAAC<br>ATAGTTTCTACGGCTCTATTTTATCTCTCACATCTTAATTAACATGTTGTCATCTAAC<br>AAAAACAAAAACAAATTCGACTTAGAGGGGATACAATTGCTGGCAAAAGGAGTCA<br>AAAATGCAGGAGAATCCATAGATAAAGCCACAGATGAGAAGCCTGAACATCTCTC<br>TGACGCATTCTCAAAATACACAGAATATTTGTCCAATGAGAGCAAGGGGGAGGAG<br>GAGGATTTCCAATTCGAACAGGTGGACTATGGATTTCAAGAGTCACCTGAATCCAA<br>TAACCCTCTGCAAGAGAGCTGGATAGCGAAAGATGACCTGGAACCAGATATGGGA<br>AAGTCCTGGGTAGAGTATCAAGCTAAGATGTCCTTTGATTACAGTGAGCAGGTGAA<br>ACCCACTGTGATGAGGGAGATCAACGGCTTGCTGGGTATGCTGGGTGGATTTGCTA<br>AATTCCAAGATGGAAAGAAAGAATATCTTTTCTATCTTCCAGAGAAGAAAGAGTCA<br>GAAGAGAGAAGGTCTGATAAGAAACAATGTCCTTTCAAGGTTGATGTCACCCCAAA<br>ACAAGACCCAAAAATCACTCCCACACCTGAGAAAGAGCCAAAACATCTGCCCACA<br>TCAGCTGCCGGCAAAGAAATCACCGAAGAAGCAGCCGTGATGCAAGGATTCTGGA<br>TGGACGGAATGAGGCTGACAGAGAAGACATCAGGGAAGTATTGCCTGTTCTTCCCA<br>CAAAAAATGGGATGGTCTCAAGCCGAATGGATCAGCAAATCAGAAGACATTAATC<br>CCCGAACTCTGGCACATGACATCTTCAAATGGATGGTATCCAAATCTCCGAAACGG<br>GCCACATACTTGAGGAAATACATGGTAGAAGAGTAACCACAGGTCTGAACCTCTTG<br>ACTATTTTTGATGCCTTAAATTGGCTTGACGTACACGTATTACTTATTACATATCTCA<br>GTTATTTATATGAAAAAAATGTTAACAGACGTCAATAACATTTGTTATATGCGCTAT<br>CATCGTGAGACTATATTCAAACGTCGAATCAATTGAAGTTAAATGGCTCTATCTATA<br>TTTAAGAAGAAGGGGAAGAAGACAGAATCTCCCAAGATGTTTCTAATGGATGCAA<br>ACCAGATGACCCCGAGTGCTCCACCCCCTTACCAGGCAGAACCTGGTCCGTTCGAC<br>ACTTGGGGGAATGAGGAACTAGAAGAAGCTATGAAAGTGTGCTACTTAGTAGACA<br>CCTGCTTGTCTGTGACAACAAGAGAACCTATTCGATCAGTGGTGGATGCCTATATCA<br>TAGCACAAGGAGTCTTAGATCATTATACCGGCCCGATCCTTACCCGTCCGTTTTACA<br>TTGCCTTGTTCTTGGGCGGGATCCACGGGATGCAAGCGGGAGTTAAAGGAGCGAGA<br>AGCATCCGGTATGAAAGAGAACATCATGGACCTCTGGTGTTCCCATATCACAGAAG<br>CAACCCGCTAGATTGGACTCCCCGGGCTATTGAATTCCAGTACACCACTAGTCTGCG<br>GGGGAAGCCGGTAGAGGTGAGCTTCCAGGCCCGTCTGCAAGCTACCAGACAATTTG<br>GACCTGGCGTAGAGGTTTATCTGGATGGCCTGCAAAGGATAGAACGCCCTAGCAAC<br>GAACTAGTTCTAAAGCAATTCAGAGTCCCCTTGCTGATGCTGGAGAAAGGGAAATG<br>GGTGTTCAATCTTGATTCTGAGGGCAGTTACAATTAAAAGGGTTCAACAATCACATC<br>ATCTGAGTCACTTTTGCTATAATTATACGGTCAAGTTTACCGCCACAAAAGGGTTGA<br>GTAGCATGTAGACACATATAAATGCATCATATTATTAATTAATTAATTGTTAAGTTA<br>TATGAAAAAAACTAACAGTGATACACCATTGTTTTGAGACATTTGTCACTGTGAAAT<br>TCAGTTGGTTCAATAGAGACATGGATACTCTGATTAAAATTCTGCTGATTATAGTCA<br>TTCTAAAGTCTCTCCATGCTCACATCGAATTCGTGCCACATGATCTGAGTAAATGGA<br>GAGACATCAGCATAGAGCATCTGGATTGTCCAATCTATGGAGATCTGTCTAATCAA<br>GCAACTAGAACAACCCCTGTGAAATACAGCAGTGTTCAATGGGGCCTAAAAAATA<br>ACATTGATGGGTACCTTTGTATATCTGCCAAATGGTCGGTCACTTGTGATTATAGGT<br>GGTATGGCTCCAAATACATTTCAACCTCAATTGAATATGTTCCAACCAAAGAATCA<br>GAATGCAGAGATGCCATCAAGTCATCAAAAAAATGGAGAGTTAGTTAATCCCCATTT<br>CATGCCCGAAAATTGTGGATGGAACAATGTGTTAACGGAGAGTGTGACATACACA	JN639009.1 Eel virus European X isolate C30, partial genome [51]

	ACTGTATCATCTCACGAGGTAAAATTAGATCCATACCAGATGACTTTCGTGGATTCA<br>CTCTTTCCGGGCGGTAAATGCTCCTCTTCAGTGTGTAGCACCATTTATCATCAAGGA<br>GTCTGGATAAACCCGAGTAACAACTTAGGGTTTTGCAAAGATCCGGTCGATCATCA<br>AGGTCAGTTGTACATGGCTGGTCTTGTTGGAGCTCACGGAGAGATTGTGAAGGAAG<br>TTTGGAATCTCAGATCTGTGTTCAAGCCAGAAATTGGTAGATCCAAGCATTTGACTG<br>GATCTTGTTGGATGACATACTGTGATCAAAGAGGACTGAGATTTTCAGATGGAGAA<br>TGGGCAGGTTTCCAAATTCCAGAGATCTCAGCACTGAAACAGGTTCTGCTAGGACT<br>TCCTGAGTGCAAAGATGATGTGCTGGTTCATGCTCATGACACGAACACTGAGTTGA<br>GGGAAATTTTGGAACACATGGACGAGTCAGCATTGAATGCCATCTGCCAACAAGA<br>AGTCCGCAGGGCAAAAGAGAGAGGAGTGGTTTCTGATTGGCTATTGAGTATGATGA<br>CACCATTCACAGAGGGGTTGGGGCCAGTTTATCGGCTGAACAAAGGAAAACTAGA<br>AGCATCAATGGGATATTATAGAAAGGTATATATAGACTCTAGCAACGCCCCTCAGG<br>CCTTCGGACAGACAGAAGATAAAGAATCTGTTGGATGGTCGGATCTTGTACCAAAA<br>GATGCAGATGGAGCTATATCATCGATGTACAATGGGAATGTCGTCATCAATAATCA<br>GATAAAATGGGCTAAAAATGCACTAGGATCTCATATATTAGATGAGATTTCTGCCTT<br>AGAATTTGAAACCCCCGTTGTGCATCATCCGCATTTGACAATCTTGTCGGTAAATCA<br>TAGTGATCTAGTGAGTTCCACACATCCAAATGGGCAAGGAGTTAATCTGATTGAGA<br>GTGTATCTCACTGGGCCGGTGGGTTGTGGGCATCCATAGGATCTGGGTTGATGATCT<br>TGGTTCTAGTGGCTTTAGTCGGATTTTGTACCATCAAGGTATGCTTAGCTTATGTTCC<br>ATCGATTTGGGCACAAAAAAATCGGAATGGTAAGAGAAGAGGAACAACATCCCAA<br>CGATCAACAGAACAGGAGATGTTCGAGCTATCAGCTGTGTAGGTACAAGTGATACT<br>TCAGGTTAAATCGGAACTCTCATTTCTGTGTAATCAGATCTATATATTAAAAGACCT<br>ACTTAAAATATTGCATGTGTTACTGATGTCAGCGTAATAAGCTGAATCATAATTTAC<br>TTCTCATGACAATTTTGTTAACCTACATGTTTTCATCTTATTACATTTTATTCTTATTTT<br>TATTTTTATGAATATATATGAAAAAAACTATTCAACAGTCATCATGTATGACGAAGA<br>TCATTCAAGAGGATATGAATCCGATGACCATTATGACCTGCCTGAATGGCTGGAAG<br>AGGATATACAATCAGGAAATCCACTCAATCAAAAAGACTATAGCTTAAATTCTCCT<br>CTCATTGTAGATCTCACAGAAGCGCTGGTAAAATATCTAAATCAAGGTAATATAGA<br>GAGACGATTCCTCAGACATCAAGACAGATTTGAGAACATACAAAGTGAAATCCGC<br>AACATTGCATGGAAGAATCCATCGAGCTCAAATCACAGATGGTGGGGGAAGTGGG<br>CTCAACAAACAGAAAAGAGTCCAGAGTTTGTAAGGCTGTTGTCTGATGTTAACCAG<br>GACATAGAAGAGACTAGTGACGTCTTGATGTCATTCTTGAAAGGATGGATCCAAGA<br>CACAACCTCTGTTCCAACAAAATTAAATTGGACATCTACTCAATTGAGCTATGGATC<br>CAAATTCTTCTTCATGCACAAATTGATCCTGTTCATGAATGCGCAATCCGATGAGGA<br>GAGGGTTATTCTCCAAGGGCACATCAAAGTCTCGGAAACAAAAAAGACTGGGATC<br>TACAAAGGTTCTCATCCTAGCTTAGGAAATTTTGTGCTGACCTCTGAATTCTTACTAC<br>TAGAACGACACAGAGTGATACTAGACCGTTCTTTCCTGTTGATGGTTAAAGATACTC<br>TAGTAGGGAGATTTCAGACACTCGCTAGCTTCATGAATAGGGAAGACAAAAAAATAT<br>CCAGAAGACGTGATAGAAAAGGTGGAGACCCTCTATTCCTTAGGAGATCAATTAGT<br>AGAGGATCYAGGGGATGAAGCATATTCGGGGATAAAGTTGCTAGAACCAGCCTGC<br>AACCTCCGACTCGCAGAGTTGGCTAGAGAATTTAGGCCTTTGATCCCCGAATTCCCA<br>CACTTTCGAAACCATGTAGAGACAGCAATAGCAGAGGAGTCTGTCTTTAGTCCTGG<br>AATCACAGAATTTTTCAACCATGTAAATAAAGAAACAAATGTTGAAATCATCTTGG<br>CCTACTTCAGCTCTTTCCGACATTGGGGACATCCATACATTGATTATTTCCAAGGGTT<br>AATCAAATTGAACAAACAAGTCACTCTAGAAAAAGACATTGATACAGAATACGCT<br>AATGCACTGGCTAGTGATTTGGCCTACATGATTCTTCGAGGACATTTTAACACTAAA<br>AGAGTCTGGGCGGTAGACAAATCACTTGTGTCAAAGCAACACCCGTTGAGTGAGCA<br>TATTTTCAATGCCACTTGGCCAACTCCGAAACAGATTGATGACTTCGGAGATCACTG<br>GCATGAATTGCCTCTGATCAAAATTTATGACATCCCAGATTTAATTGACCCATCTGT<br>CATCTACTCTGACAAAAGTCATTCAATGGGGAGAGAAGAAGTGTTAAAACATGTTC<br>AAAGGAATCCAACACAAGCAATTCCTACAAAAAAGGTCTTAGAGACACTCTTACA<br>AAAACCTGCCACAAATTGGCCAGAATTCTTGTCCTCGATTGAAAAAGATGGCCTAC<br>CTAAAGACAGCTTGATTATCGGACTGAAGGGAAAAGAGAGGGAACTAAAAAAAG<br>CTGGAAGGTTCTTTTCTCTAATGTCCTGGGAATTGAGGGAGTATTTTGTAATAACTG<br>AATACCTGATCAAAACTCATTACGTCCCTCTCTTTAAAGGATTGACCATGGCAGATG<br>ACATGACAGAGGTCGTCAAGAAGATGTTAGAAAGAAGTCAAGGACAAGGAGAGG<br>ATGATTACGAGCATGTCAGCATTGCCAACCACATTGATTATGAAAAATGGAACAAC<br>CATCAAAGGAAAGAGTCTAACGGCCCAGTGTTCAGAGTCATGGGACAATTTCTGGG<br>GTACCCTAGTTTGATAGAAAAGACCCATGACTTCTTTGAGCAAAGCTTGATCTATTA<br>TAATGGCAGACCTGACTTGATGCAGACAGACGGAGATGAGTTGCAAAACCGAACG<br>GAAGCTTTGGTGTGTTGGAATGGTCAGAAAGGAGGATTAGAAGGACTCAGGCAAA<br>AAGGATGGAGCATCCTCAATCTGTTGGTCATCAAAAGAGAATCTAAAATCAGAAA<br>CACTAAAGTGCAAACTTTGGCCCAGGGAGACAATCAGGTAGTGTGTACCCAATACC<br>GCATTATGCCAACCAGATCGACACTTGAATTGCAGGCAGAACTTGAAAAAGTTAAG<br>AAGAACAATCAAGTTATTATGGATGCTATTGAGACAGGGACTAACAAATTAGGGCT<br>ACTGATAAACAATGATGAAACCATTCAATCCGCTGATTTCCTAACCTATGGTAAGG<br>TTCCAATATTTAGAGGTAATATTCGCTGTTTGGAAACTAAAAGATGGTCTAGAGTGA<br>CTTGTGTCACTAATGATCAATTGCCATCCCTTTCCAATGTCATGTCATCTGTGTCGAC<br>AAACAGTCTGACGGTCTCGCATTTCGATGTCAGTCCTATAGAATCCATGAGACAAT<br>ACCTGTTCTTCGGAAATTTTGCTCGGAGATTGGTGGAATTCCACAATCCTGCGATGA<br>GAGTGCCGATTAGTCTAGAAGACCTGGATTCCAAACAAAAGTCTGTTTATCTGAAC<br>GCAGTGCTTTTCTTGGACCCATCTCTGGGTGGCGTGTCTGGTATGTCATTGTCAAGAT<br>TCTTGACTAGGATGTTTCCTGATCCCATCACAGAAGGATTATCTTTTTGGAAAATCG<br>TTTACGAACATACCACTTCCAAAGACACTCAACTGCTCTGCAGAATCGCTGGAAGC<br>CCCGAATTGGCTAGACGCCAGAACAATCTGGACAAGTTGATAGAGAATCCAACTG<br>CATTGAATCTGAGCAAAGAAACTTCTGCCCTAAGTGTCATTAAGAAAGAAGTCAGG<br>TCACGCCTGTACAAGGATTGCGACAAATTCAAGAACAAATTGATTGCAGATGCAAT<br>TGGGATTGCTAGAGATGAAGAGGCTCACCTGGAATTATTCTTAATGTCAATCCGAC<br>CATTATTTCCGAGATTCCTGGCAGAGTTCAAAGCAGCAACATTTGTTGGCATCACAG<br>AATCTCTCATTTCTTTATTCCAAAATTCCAAAACCATTCGCAACATCTTCAGGAAGA	

	AATATGCGAAAGAATTGGAACTTAGAGTAGTGCAGTGTGAATACAGATCCATCAAT<br>TTGATGTTGAGCCTCGCAGATCGATCCCACTTAGATGAAATGTGGACATGCTCGGC<br>ATCTAAAGCAGACGAACTCCGAACCTTATCATGGGGGACTACAATAATTGGAACA<br>ACTGTCCCTCATCCTTTAGAGATGATCAATCATGCTCATATAGGACAGAAATGCGA<br>CTCGTTAGAAACCCTGGACTACATAAATGTCACTGTGGTTCAAGATCTGACTGATTG<br>TCTAACTAGCAAAGGGAAGTTACCAGCGTACTTAGGATCCAAAACATCAGAGACT<br>ACAAGCATCTTGCAACCATGGGAAAAGGAGACAAAGATTCCTGTGATTCGTAGAG<br>CTGCTAAATTGAGAGCTGCAATCACCTGGTTTGTAGAGCCTGATTCTCTTTTGGCAC<br>AAAGCATCCTGAACAACATTGAAAGTCTGACTGGAGAAGATTGGTCTGCTTCAATA<br>TCTGGATTCAAAAGGACAGGATCCGCACTACATCGCTTCACTAGTGCTAGAGTGAG<br>TGCAGGAGGCTTTTCAGCTCAAAGCCCGGCCAGATTGACCAGGATGATGGCAACAA<br>CAGATACTTTCCGAGAAATTGGGTCAGATAATTACGATTTTATGTTCCAATCATTAC<br>TACTCTTTGCCCAAATGACTACAGGTGAGATTTACAAAAGGAGTCCAGCTACTAATT<br>TCCACTTTCATCTGAGTTGCCACCAATGTCTTCGTAAGATCGAAGAGCCTACCTTAA<br>ACTCTGATTTTGCCTACAACCCTATTCAGAGATCGGACATCTTGGATAAGTGGAAAC<br>CTCAGACCACAGATTGGTCTTCAGAAAGAAAGGCTCCGGAGATAGAGGAAGGGAA<br>TTGGGACAGATTGACACATCAGGAGCAAAGTTTTCAGATAGGGAAGTCCATCGGGT<br>TTCTGTTTGGAGATTTGACAATGACAAAGAATTCACATGCTCAGGATTCTTCTATTTT<br>CCCTTTATCAATTCAATACAAGATCACAGCTGCGGAGTTTCTGGAAGGAATTCTAGA<br>TGGGATAGTCAAGGCTTCTGCTTTGTCTACTATCCACCGGAGGAACTTCGATCATCA<br>CAGCAAGTACAAGTCCACTGTTTCAGGAACTGTTGATTATTTAATCGAATTGATTTC<br>AGAGTCCGCGGGGTTCACAAATCTGACCAGGAATGGTCCCTTGAAGGCTTGCTTAC<br>TGACCATTCCACATAAGATACCTCCATCCTATCCTCTCAGCCAATCTGATCTTGGTG<br>CTATGTCAAGAAACTACCTCAGACTGTTGCACCGAAGAATGTCTACCGGCACCTAC<br>AAGACCAGGTGGCCGACAAATTGGATATTCTCTGATATGATGAGCCCTAACATAAT<br>CTATCCATTTGTGATCAGTGTCTCCTGCGTTGGACTGGCTTACTCATCATCTTGGACA<br>AAGAAATCTGCTGACAAATTGAGAGGGTTAAGAGGAGTAGCTGAGTTGATTCGATC<br>ATCAGATGATGTGCAATTGCCTGTTGGGAAGCTATTCAAAACAGTAAACCAAGAAA<br>TTAGACATGCCATAAAACATCATGCATCGGACGATGCTGAAATCCCCGAAAGCCAC<br>ATCCAAGAAGGCTGGAAAAAAGAGCTCATAGTCAACATTAACATGCAGCCAATTG<br>ACTATAGTAGAACAGCGACAGCGAAGTCGTTAGACCGGCCTGCTCAAATTCGAGA<br>CCCTTTAATATCTGGACTAAGAACAGCACAATTGGCCACTGGATCCCACTACAAGT<br>TACGATCCATACTAGTGCAAAACCGAATCCAAGTTACCGATGCTCTGTGCGGAGGG<br>GATGGATCTGGTGGAATCGGGGCTTGTGTCCTGAGACAATACCCGTTTGCTAAGCTG<br>ATTTACAACAGTCTATTCGAGATTCAAGATCTGGATATGAGGGGGAGTGCACCTGG<br>ACCGCCATCTGCTATTGCCGCAATGGGAAACATGAGTATGAGGTGTGTCAATCGAG<br>ATTCTGCATGGAAAAATCCATCTGATCTGAGCCACACTTCCACCTGGGAATATTTTC<br>AATCGTTGATGACCCAACATCAGTTGCGGTGCAATCTGTGGACCTTTGACATGGAG<br>GTTCGCAGCCATGACATTTCAGATGCTATAGAAAAGCAAATAGTGGCCAATCTTCA<br>TTTGCTACCAAAAAATGGGACAATCATATACAAGACATATCTAACGAAATTGTCTG<br>ACATGGAGACAACAATCTTGGACAGACTGGGTGGATTCTTCAAAAGAGTCAGTCTT<br>GTGTCTACAGACGCTACATCAAGCCATAGCTCTGAAGTCTACGCTGTATTCCAAAA<br>CAAACTGGATAAGAGACAATTGGAAATCCACCCCAACTGGTCTTCCTGCAATTTAG<br>GTTCTGACATACACCCATGCTGGAAAAGTGAAGATGAGGAGTTTGAAAGGGCAAG<br>AAGATTCTTTCACATGAAGAGACAACAAGGGGTGCCGACTCGATTGAGACCTACTT<br>TGGATTCCGAAATCCAGGTCCTGAGCGTCTCGGCCGGAGTTGAAAACGGGGTAGCA<br>ATGACATTAGCTATGGACGTAAGCAATCAGGTCTCGGATCCAACCACTGGGGCATT<br>TCTTTGGCTTTTAGTAACTTTGCAACATATATGCCCAATAGGCCCATCATTCAAGAT<br>CCCATCCAGTTCTGCAGTTGAATCTTATTTGGCCATACTGATTGGATTCAGTAGTATC<br>TACCAGCTTCAGACAGGCAATAACAAAGCTTATGCCAGCATCAAGACCTGCTTGTC<br>TCAATCTGCTCCATTCTTCTGCAACCCTGATGGATGGAGTTGCATTAAAGGACTTGA<br>CAAGTCTATGCGGATGGACAGAAAGTTGGCTTTAGTGGGCAGTGTGATACGGGCAT<br>GGTCAAAGTGGAACTTGACTCAGTCAATCAACTTTCACAAACTAGATGGTATGATG<br>AGACATTATCTACCGAAGGGAACATTGAGGCAGATTGGCTGCAAAACCGGGATCT<br>GGGACTATATCAATGGGGCAGTGAAAGGAGTACGAAGTGCTGCCAATCAGGACAG<br>TCCAAAAGAGTCAGCAGCTGCTTGGAGAGATTAGTATATATAACATTGTGTCTATC<br>ATCCAATTCATTATTAGATAGGTGACAATTAGTTAAAGAACAAGATTACCCCATGT<br>AAGAGAATTTTTTATGTCCTAATTTGCCGTATGCACTTGTAAATAAATGTGTATTTTA<br>ATGTATATGTGTATGTGTATTTGATGTATTGGTGTATATGTCAACAAATCAATAATAT<br>GATCACATAGGATAAGGTCAAGACCAATTGTTTAAGTTTTATTAGAATTCCGTGAAT<br>ATGAAAAAAACAAAACAGGTCCTTTTGGATAAATCCTTAATAATGGGG	
JN639010	TAAGGCTATTCTTTAACAGACATCTGTTTGTTTCATTGAAAATGGCTGCCATGAACT<br>CCATCAAAGTTACAGTTAGAGCCAATGGACAAGATTTCACCCCAGTTTGTCCTAAA<br>ATGGAGGATGAAGTGGCTTACCCATCTGATTATTTAGATGCTAATGGATTACCAACC<br>TTCCAGCTCTACTATCATGATCTGTCAAAAAAGGATCTGCTAGACCTGATCTGGGGA<br>GAAGCATCAGAGGCAAGGCTGCCTAGTGAGCTGGTGACTGCGTACGTGTACAAAGT<br>GGTGAGTGAGTGGAAAGAGAAGCTGGAGAGTGATTGGTCATCTTTCCGATTCCAGA<br>TTGGCAAAGCGGGAGAGGAGATAACCCCATTCAACCTGATTGGAATGACTGTGAA<br>CAGTCAGAAACTAGCTGACTACAAAAAAGCAGTTGCTCCTGAGGGGATAGATGAG<br>GTGGCAATGGTGATCTATCTCCTGGCACCATACCGGATAGTCGGAATTAAAAACGA<br>AGACTATCAAGATCGGGTGATCACCAACATCCAGAACCAGCTAGACAGTCTCGGC<br>GCAAAAAAGCTGCAGGTGAAGGCACTGAAGAATGTCACTACTCTCATCAACAGTG<br>CCAACTATCTGAGAATGGTGGCAGTGATTGACATGTTCTACTACCATTTCAAGAACA<br>GTCAAGAAAGAGCTGTTGTCAGGATTGCCACTCTGAGTTCCCGTCACAAAGACTGT<br>GCAGCTTTATCCACTCTGAATCACATCACCAGCTTCACTGGGAGGAGTTTTGTGCAG<br>GTGTTGGATTGGGTTTTCACAGATCAAGTTGCCAAGGAGATCGGCAGGATGATGCG	JN639010.1 Eel virus European X isolate GG129, partial genome<br>[51]

	TGCGGGTCAAGAAATTGATCGTCCTGAATCCTACATGCCGTACCTGAAGGACTTGG<br>GGCTGAGCAGAAAATCCCCCTACTCCTCCTCAGCAAATCCTGGCACTCACTGCTGG<br>GCACAGATGGTATGTGCCATGATGGGATCCAAAAGATCTCAAAACGCAATCGCCA<br>GCACTGAAGAGAATCTCAGCAATCTGACAAGAAATGCTGAGATTATGGCCTATGCC<br>CTGGGAGTGGGAGCAGACCTGGTGAAGGGACTGATCATTGGAGATCAGAAAGAAG<br>GAGACTCCGGTGTCATCCAGGATGAGGAAGGAATGGATGAGCCCAACAACATGGA<br>GGCCCAAGATTGGCTGGAGTACATGGCCTCAAAGGGATTCAAGCTGACTCCAAATA<br>TGGAGCTGCAAGTCCGCCACATGTGCCTGCGAATCACCAATCCCAGAAAAGCCACT<br>CTGGGGAGTTACCTGCGGGAGAGATACAGTTCATCCCTGTGAGTTGAAGCAGAAAT<br>GAGTCATTTTTAGAACTATTATATTTTTATCCCAATAGCCTTAAATAGCTAGTCATCC<br>TGTGCTTAGATATTATGCCCTAGTTATATGAAAAAAACTTTAACAGAGATAAACAT<br>AGTTTCTACGGCTCTATTTTATCTCTCACATCTTAATTAACATGTTGTCATCTAACAA<br>AAACAAAAACAAATTCGACTTAGAGGGGATACAATTGCTGGCAAAAGGAGTCAAA<br>AATGCAGGGGAATCCATAGATAAAGCCACAGATGAGAAGCCTGAACATCTCTCTG<br>ACGCATTCTCAAAATACACAGAATATTTGTCAAATGAGAGCAAGGGGGATGAGGA<br>GGATTTCCAATTTGAACAGGTGGACTATGGATTTCAAGAGTCACCTGAATCCAATA<br>ACCCTCTGCAAGAGAGCTGGGTAGCGAAAGATGACCTGGAACCAGATATGGGAAA<br>GTCCTGGGTAGAGTATCAAGCTAAGATGTCTTTTGATTACAATGAGCAGGTGAAAC<br>CCACTGTGATGAGGGAGATCAACGGCTTGCTGGGTATGCTGGGTGGATTTGCTAAA<br>TTCCAAGATGGAAGGAAAGAATATCTTTTCTATCTTCCAGAGAAGAAAGAGTCAGA<br>AGAGAGAAGGTCTGATAAGAAACAATGTCCTTTCAAGGTTGATGTCACCCCAAAAC<br>AAGACCCAAAAATCACTCCCACACCTGAGAAAGAGCCAAAACATCTGCCCACATC<br>AGCTGCCGGCAAAGAAATCACCGAAGAAGCAGCCGTGATGCAAGGATTCTGGACG<br>GACGGAATGAGGCTGACAGAGAAGACATCAGGGAAGTATTGCCTGTTCTTCCCACA<br>AAAAATGGGATGGTCTCAAGCCGAATGGATCAGCAAATCAGAAGACATTAATCCC<br>CGAACTCTGGCACATGACATCTTCAAATGGATGGTATCCAAATCTCCGAAACGGGC<br>CACATACTTGAGGAAATACATGGTAGAAGAGTAACCACAGGTCTGAATCTCTTGAC<br>TATTTTTGATGCCTTAAATTGGCTTGACGTACACGTATTACTTATTACATATCTCAGT<br>TATTTATATGAAAAAAATGTTAACAGACGTCAATAACATTTGTTATATGCGCTATCA<br>TCGTGAGACTATATTCAAACATCGAATCAATTGAGGTTAAATGGCTCTATCTATATT<br>TAAGAAGAAGGGGAAGAAGACAGAATCTCCCAAGATGTTTCTAATGGATGCAAAC<br>CAGATGACCCCGAGTGCTCCACCCCCTTACCAGGCAGAACCTGGTCCGTTCGACAC<br>TTGGGGGAATGATGAGCTAGAAGAAGCTATGAAAGTGTGCTACTTAGTAGACACTT<br>GCTTGTCTGTGACAACAAGAGAACCTATTCGATCAGTGGTGGATGCCTATATCATA<br>GCACAAGGAGTCTTGGATCATTATACCGGCCCGATCCTTACCCGTCCATTTTACATT<br>GCCTTGTTCTTGGGCGGGATCCACGGGATGCAGGCGGGAGTTAAAGGAGCGAGAA<br>GCATCCGGTATGAAAGAGAACATCATGGACCTCTGGTGTTCCCATATCACAGAAGC<br>AACCCGCTAGATTGGACTCCCCGGGCTATTGAATTCCAGTACACCACTAGTCTGCG<br>GGGGAAGCCGGTAGAGGTGAACTTCCAAGCCCGTCTGCAAGCTACCAGACAATTTG<br>GACCTGGAGTAGAGGTTTATCTGGATGACCTGCAAAGGATAGAACGCCCTAGCAAC<br>GAACTAGTTCTAAAGCAATTCAGAGTCCCCCTGCTGATGCTGGAGAAAGGGAAATG<br>GGTGTTCAATCTTGATTCTGAGGGCAGTTACAATTGAAAGGATTCAACAATCACATC<br>ATCTGAGCCACTTTTGCTATAATTATACAGTCAAGTTTACCGCCACAAAAGGGTTGA<br>GTAGCATGTAGACACATATAAATGCATCATATTATTAATTAATTAATTGTTAAGTTC<br>TATGAAAAAAACTAACAGTGATACACCATTGTTTTGAGACATTTGTTACTGTGAAAT<br>TCAGTTGGTTCAATAGAGACATGGATACTCTGATTAAAATTCTGCTGATTATAGTCA<br>TTCTAAAGTCTCTCCATGCTCACATCGAATTCGTGCCACATGATCTGAGCAAATGGA<br>GAGACATCAGCATTGAGCATCTGGATTGTCCAATCTATGGAGATCTGTCTAATCAA<br>GCAACTAGAACAACCCCTGTGAAATACAGCAGTGTTCAATGGGGCCTAAAAAATA<br>ACATTGATGGGTACCTTTGTATATCTGCCAAATGGTCGGTCACTTGTGATTATAGGT<br>GGTATGGCTCCAAATACATTTCAACCTCAATTGAATATGTTCCAACCAAAGAATCA<br>GAATGCAGAGATGCCATCAAGTCATCAAAAAAATGAAGAGTTAGTTAGTCCTCATTT<br>CATGCCCGAAAATTGTGGATGGAACAATGTGTTAACGGAGAGTGTGACATACACA<br>ACTGTATCATCTCACGAGGTAAAATTAGATCCATACCAGATGACTTTCGCGGATTCA<br>CTCTTTCCGGGCGGTAAATGCTCCTCTTCAGTGTGTAGCACCATTTATCATCAAGGA<br>GTCTGGATAAACCCTAGTAACAATTTAGGATTTTGCAAAGATCCGGTCGATCATCA<br>AGGTCAGTTGTACATGGCTGGTCTTGTTGGAGCTCGCGGAGAGATTGTGAAGGAAG<br>TTTGGAATCTCAGATCTGTGTTCAAGCCAGAGATTGGTAGATCCAAGCATTTGACTG<br>GATCTTGTTGGATGACATACTGTGATCAAAGAGGACTGAGATTTTCAGATGGAGAA<br>TGGGCAGGTTTCCAAATTCCAGAGATCTCAGCACTGAAACAGGTTCTGCTAGGACT<br>TCCTGAGTGCAAAGATGATGTGCTGGTTCATGCTCATGACACGAACACTGAGTTGA<br>GGGAAATTTTGGAACACATGGACGAGTCAGCATTGAATGCCATCTGCCAACAAGA<br>AGTCCGCAGGGCAAAAGAGAGAGGAGTGGTTTCCGATTGGCTATTGAGTATGATGA<br>CACCATTCACAGAGGGGTTGGGGCCAGTTTATCGGCTGAACAAAGGAAAACTAGA<br>AGCATCAATGGGATATTATAGAAAGGTATATATAGACTCCAGCAACGCCCCTCAGG<br>CCTTCGGACAGACAGAAGACAAAGAATCTGTTGGATGGTCGGATCTTGTACCAAAA<br>GATGCAGATGGAGCTATATCATCGATGTACAATGGGAATGTCGTCATCAATAATCA<br>GATAAAATGGGCTAAAAATGCACTAGGATCTCATATATTAGATGAGATTTCTGCCTT<br>AGAATTTGAAACCCCCATTGTGCATCATCCTCATTTGACAATCTTGTCGGTAAATCA<br>TAGTGATCTAGTGAGTTCCACACATCCAAATGGGCAAGGAGTTAATCTGATTGAAA<br>GTGTATCTCACTGGGCCGGTGGGTTGTGGGCATCCATAGGATCTGGGTTGATGATCT<br>TGGTTCTAGTGGCCTTAGTCGGATTTTGTACCATCAAGGTCTGCTTAGCTTATGTTCC<br>ATCGATTTGGGCACAAAAAAATCAAAATGGTAAGAGAAGAGGAACAACATCCCAA<br>CGATCAACAGAACAGGAGATGTTCGAGCTATCAGCTGTGTAGGTACAAGTGATACT<br>TCAGGTTAAATCGGAACTTTCATTTCCGTGGAATCAGATCTATATATTAAAAGACCT<br>ACTTAAAATATTGCATGTGTTACTGATGTCAGCGTAATAAGCTGAATCATAATCTAC<br>TTCTCATGACAATTTGGTTAACCTACATGTTTTCATCTTATTACATTTTATTCTTATTTT<br>TATTTTATGAATATATATGAAAAAAACTGTTCAACAGTCATCATGTATGACGAAGAT<br>CATTCAAGAGGATATGAATCCGATGACCATTATGACCTGCCTGAATGGCTGGAAGA<br>GGATATACAATCAGGAAATCCACTCAATCAGAAAGACTATAGCTTAAATTCTCCTC	

	TCATTGTAGATCTCACAGAAGCGCTGATAAAATATCTAAATCAAGGTACTATAGAG<br>AGACGATTCCTCAGACATCAAGACAGATTTGAGAACATACAAAGTGAAATCCGCA<br>ACATTGCATGGAAGAATCCATCGAGCTCAAATCACAGATGGTGGGGGAAGTGGGC<br>TCAACAAACAGAAAAGAGTCCAGAGTTTGTAAGGTTGTTGTCTGATGTTAACCAAG<br>ACATAGAAGAGACTAGTGACATCTTGATGTCATTCTTGAAAGGATGGATCCAAGAC<br>ACAACCTCTGTTCCAACAAAATTAAATTGGACATCTACTCAATTGAGCTATGGATCT<br>AAATTCTTCTTCATGCACAAATTGATCCTGTTCATGAATGCGCAATCCGATGAGGAG<br>AGGGTTATTCTCCAAAGGCACATCAAAGTCTCGGAAACAAAAAAGACTGGGATCT<br>ACAAAGGTTCTCATCCTAGCTTAGGAGATTTTGTGCTGACCTCTGAATTCTTACTACT<br>AGAACGACACAGAGTGATACTAGACCGTTCTTTCCTGTTGATGGTTAAAGATACTCT<br>AGTAGGGAGATTTCAGACACTCGCTAGCTTCATGAATAGGGAAGACAAAAAATAT<br>CCAGAAGACGTGATCGAAAAGGTGGAGACCCTCTATTCCTTAGGAGATCAATTGGT<br>AGAGGATCTAGGGGATGAAGCATATTCGGGGATTAAGTTGCTAGAACCAGCCTGCA<br>ACCTCCGACTCGCAGAGTTGGCTAGAGAATTCAGGCCTTTGATCCCCGAATTCCCAC<br>ACTTTCGAAATCATGTAGAGACAGCAATAGCAGAGGAGTCTGTCTTTAATCCTGGA<br>ATCACAGAATTTTTCAACCATGTAAATAAAGAAACAAATGTTGAAATCATCTTGGC<br>CTACTTCAGCTCTTTCCGACATTGGGGACATCCATACATTGATTATCTCCAAGGGTT<br>AATCAAATTGAACAAACAAGTCACTCTAGAAAAAGATATTGATACAGAATACGCT<br>AATGCACTGGCTAGTGATTTGGCCTACATGATTCTTCGAGGACATTTTAACACTAAA<br>AGAGTCTGGGCAGTAGACAAATCACTTGTGTCAAAGCAACACCCGTTGAGTGAGCA<br>TATTTTCAATGCCACTTGGCCAACTCCGAAACAGATTGATGACTTCGGAGATCACTG<br>GCATGAACTGCCTCTGATCAAAATTTATGACATCCCAGATTTAATTGACCCATCTGT<br>CATCTACTCTGACAAAAGCCATTCAATGGGGAGAGAAGAAGTGTTAAAACATGTTC<br>AAAGGAATCCAACACAAGCAATTCCTACAAAAAAGGTCTTAGAGACACTCTTACA<br>AAAACCTGCCACAAATTGGCCAGAATTCTTGTCCTCGATTGAAAAAGATGGCCTAC<br>CTAAAGACAGCTTGATTATCGGACTGAAGGGAAAAGAGAGGGAACTAAAAAAAG<br>CTGGAAGATTCTTTTCTCTAATGTCCTGGGAACTGAGGGAGTATTTTGTAATAACTG<br>AATACCTGATCAAAACTCATTACGTCCCTCTTTTTAAAGGATTGACTATGGCAGATG<br>ACATGACAGAGGTTGTCAAGAAGATGTTAGAAAGAAGTCAAGGACAAGGAGAGG<br>ATGATTACGAGCATGTCAGCATTGCCAACCACATTGATTATGAAAAATGGAACAAC<br>CATCAAAGGAAAGAGTCTAACGGCCCAGTGTTCAGAGTCATGGGACAATTTCTTGG<br>GTACCCTAGTTTGATAGAGAAGACCCATGACTTCTTTGAGCAAAGCTTGATCTATTA<br>TAATGGCAGACCTGACTTAATGCAGACAGACGGAGATGAGTTGCAAAACCGAACG<br>GAAGCTTTGGTGTGTTGGAATGGTCAGAAAGGAGGATTAGAAGGACTCAGGCAAA<br>AAGGATGGAGCATCCTCAATCTGTTGGTCATTAAAAGAGAATCTAAAATCAGAAAC<br>ACTAAAGTGCAAACTTTGGCCCAGGGAGACAATCAGGTAGTGTGTACCCAATACCG<br>CATTATGCCAACCAGATCGACACTTGAATTGCAGGCAGAACTTGAAAAAGTTAAGA<br>AGAACAATCAAGTTATTATGGATGCTATTGAGACAGGGACTAACAAATTAGGGCTA<br>CTGATAAACAATGATGAAACCATTCAATCCGCTGATTTCCTAACATATGGTAAGGTT<br>CCAATATTTAGAGGTAATATTCGCTGTTTGGAAACCAAAAGATGGTCTAGAGTGAC<br>TTGTGTCACTAATGATCAATTGCCATCCCTTTCCAATGTCATGTCATCTGTGTCGACA<br>AACAGTCTGACGGTCTCGCATTTCGATGTCAGTCCTATAGAATCCATGAGACAATA<br>CCTGTTCTTCGGAAATTTTGCCAGGAGATTGGTGGAATTCCATAATCCTGCGATGAG<br>AGTGCCGATTAGTCTAGAAGATCTGGATTCCAAACAAAAGTCCGTTTATCTGAACG<br>CAGTGCTTTTCTTGGACCCATCTCTGGGTGGCGTGTCTGGTATGTCATTGTCAAGATT<br>CTTGACTAGGATGTTTCCTGATCCCATCACAGAAGGATTATCTTTTTGGAAAATTGTT<br>TACGAACATACCACTTCCAAAGACACTCAACTGCTTTGCAGAATCGCTGGAAGCCC<br>CGAATTGGCTAGACGCCAGAACAATCTAGACAAGTTGATAGAGAATCCAACAGCA<br>TTGAATCTGAGCAAAGAAACTTCTGCCCTAAGTGTCATTAAGAAAGAAGTCAGGTC<br>ACGCCTGTACAAGGATTGCGACAAATTCAAGAACAAATTGATTGCAGATGCAATTG<br>GGATTGCTAGAGATGAAGAGGCTCACCTGGAATTATTCTTAATGTCAATCCGACCA<br>TTATTTCCGAGATTCCTGGCAGAGTTCAAAGCAGCAACATTTGTTGGCATCACAGAA<br>TCTCTCATTTCTTTATTCCAAAATTCCAAAACCATTCGCAACATATTCAGGAAGAAA<br>TATGCGAAAGAATTGGAACTTAGAGTAGTGCAGTGTGAATACAGATCCATCAATTT<br>GATGCTGAGCCTCGCAGATCAATCCCACTTAGATGAAATGTGGACATGCTCGGCAT<br>CTAAAGCAGACGAACTCCGAACCTTATCATGGGGGACTACAATAATTGGAACAACT<br>GTCCCTCATCCTTTAGAGATGATCAATCATGCTCATATAGGACAGAAATGCGACTC<br>GTTAGACACCCTGGACTACATAAATGTCACTGTGGTTCAAGATTTGACTGATTGTCT<br>AACTAGCAAAGGGAAGTTACCAGCGTACTTAGGATCCAAAACATCAGAGACTACA<br>AGCATCTTGCAACCATGGGAAAAGGAGACAAAGATTCCTGTGATTCGTAGAGCTGC<br>TAAATTGAGAGCTGCAATCACCTGGTTTGTAGAGCCTGATTCTCTTTTGGCACAAAG<br>CATCCTGAACAACATTGAAAGTCTGACTGGAGAAGATTGGTCTGCTTCAATATCTG<br>GATTCAAAAGGACAGGATCTGCACTACATCGCTTCACTAGTGCTAGAGTGAGTGCA<br>GGAGGATTTTCAGCTCAAAGCCCGGCCAGATTGACCAGGATGATGGCGACAACAG<br>ATACTTTCCGAGAAATTGGGTCAGATAATTACGATTTTATGTTCCAATCATTACTACT<br>CTTTGCCCAAATGACTACAGGTGAGATTTACAAAAGGAGTCCAGCTACTAACTTCC<br>ACTTTCATCTGAGTTGCCACCAATGTCTTCGTAAGATCGAAGAGCCTACCTTAAACT<br>CTGATTTTGCCTACAACCCTATTCAGAGATCGGACATCTTGGATAAGTGGAAACCTC<br>AGACCACAGATTGGTCTTCAGAAAGAAAGGCTCCAGAGATAGAGGAAGGGAATTG<br>GGACAGATTGACACATCAGGAGCAAAGTTTTCAGATAGGGAAGTCCATCGGGTTTC<br>TGTTTGGAGATTTGACAATGACAAAGAATTCACATGCCCAGGATTCTTCTATTTTCC<br>CTTTATCAATTCAATACAAGATCACAGCTGCGGAGTTTCTGGAAGGAATTCTAGATG<br>GGATAGTCAAGGCTTCTGCTTTGTCTACTATCCACCGGAGGAACTTCGATCATCACA<br>GCAAGTACAAGTCCACTGTTTCAGGAACTGTTGATTATTTAATCGAATTGATTTCAG<br>AGTCCGCTGGGTTCACAAATCTGACCAGGAACGGTCCCTTGAAAGCTTGCTTACTG<br>ACCATTCCACATAAGATACCTCCATCCTATCCTCTCGCCAATCTGATCTTGGTGCTAT<br>GTCAAGAAACTACCTCAGACTGTTGCACCGAAGAATGTCTACCGGCACCTACAAGA<br>CCAGGTGGCCGACAAATTGGATATTCTCTGATATGATGAGCCCTAACATAATCTATC<br>CATTTGTGATCAGTGTCTCCTGCGTTGGACTGGCTTACTCGTCATCTTGGACAAAGA<br>AATCTGCTGACAAATTGAGAGGGTTGAGAGGAGTAGCTGAGTTGATTCGATCATCA	

	GATGATGTGCAATTGCCTGTTGGGAAGCTATTCAAAACAGTAAACCAAGAAATTAG<br>ACATGCCATAAAACATCATGCATCGGATGATGCAGAAATCCCCGAAAGCCACATC<br>CAAGAAGGCTGGAAAAAAGAGCTCGTAGTCAACATTAACATGCAGCCAATTGACT<br>ATAGTAGAACAGCGACAGCGAAGTCGTTGGACCGGCCTGCTCAAATTCGAGACCCT<br>TTAATATCTGGACTAAGAACAGCACAATTGGCCACTGGATCCCACTACAAGTTACG<br>ATCCATACTAGTGCAAAACCGAATCCAAGTTACCGATGCTCTGTGCGGAGGGGATG<br>GATCTGGTGGAATCGGGGCTTGTGTCCTGAGACAATACCCGTTTGCTAAACTGATTT<br>ACAACAGTCTATTCGAGATTCAAGATCTGGATATGAGGGGGAGTGCACCTGGACCG<br>CCATCCGCTATTGCCGCAATGGGGAACATGAGCATGAGGTGTGTCAATCGAGATTC<br>TGCATGGAAAAATCCATCTGATCTGAGCCACCCTTCCACCTGGGAATATTTTCAATC<br>GTTGATGACCCAACATCAGTTGCGGTGCAATCTGTGGACCTTTGACATGGAGGTTCG<br>CAGCCATGACATTTCAGATGCGATAGAAAAGCAAATAGTGGCCAATCTTCATTTGC<br>TACCAAAAAAATGGGACAATCATATACAAGACATATCTAACGAAATTGTCTGACATG<br>GAGACAACAATCTTAGACAGACTGGGTGGATTCTTCAAAAGAGTCAGTCTTGTGTC<br>TACAGACGCTACATCAAGCCATAGCTCTGAAGTCTACGCTGTATTCCAAAACAAAC<br>TGGATAAGAGACAATTGGAAATCCACCCCAACTGGTCTTCCTGCAATTTAGGTTCTG<br>ACATACACCCATGCTGGAAAAGTGAAGATGAGGAGTTTGAAAGGGCAAGAAGATT<br>CTTTCACATGAAGAGACAACAAGGGGTGCCGACTAGATTGAGACCCACTTTGGATT<br>CCGAAATCCAGGTCCTGAGCGTCTCGGCTGGAGTTGAAAACGGGGTAGCAATGAC<br>ATTAGCTATGGACGTAAGCAATCAGGTCTCGGATCCAACCACTGGGGCATTTCTTTG<br>GCTTTTAGTAACTTTGCAACATATATGCCCAATAGGCCCATCATTCAAGATACCATC<br>CAGTTCTGCAGTTGAATCTTATTTGGCCATACTGATTGGATTCAGTAGTATCTACCAG<br>CTTCAGACAGGCAATAACAAAGCCTATGCCAGCATCAAGACCTGCTTGTCTCAATC<br>TGCTCCATTCTTCTGCAACCCTGATGGATGGAATTGCATTAAAGGACTTGACAAGTC<br>TATGCGGATGGACAGAAAGTTGGCTTTGGTGGGCAGTGTGATACGGGCATGGTCAA<br>AGTGGAACTTGACTCAGTCGATCAACTTTCACAAATTAGATGGTATGATGAGACAT<br>TATCTACCGAAGGGAACATTGAGGCAGATTGGCTGCAAAACCGGGATCTGGGACT<br>ATATTAATGGGGCAGTGAAAGGAGTACGAAGTGCTGCCAATCAGGACAGTCCAAA<br>AGAGTCAGCAGCTGCTTGGAGAGATTAGTATATATAACATTGTGTCTCTCATCCAAT<br>TTATTATTAGATAGGTGACAATTAGTTAATGAACAAGATTACCCCATGTAATAGAAT<br>TTTTTATGTCCTAATTTGCTGTATGCACTTGTAAATAAATATGTATTTCAATGTATAT<br>GTGTATGTGTATTTGATGCATTGGTGTATATGTCAACAAATCAATAATATGATCACA<br>TAGGATAAGGTCAAGACCAATTTTTAATTTTTATTAGAAGTCCGTAAATATGAAAAA<br>AACAAAACAGGTCCTCTCGGATAAATCCTTAATAATGGGG	
JX827265	AAAACCCATTAAACGACTTAATACTTTTCTCTGCCTTTTAAGGCTATTCTTTAACAGA<br>CATCTGTTTGTTTCATTGAAAATGGCTGCCATGAACTCCATCAAAGTTACAGTTAGA<br>GCCAATGGACAAGATTTCACCCCAGTTTGTCCTAAAATGGAGGATGAAGTGGCTTA<br>CCCATCTGATTATTTAGATGCTAATGGATTACCAACCTTCCAGCTCTACTATCATGAT<br>CTGTCAAAAAAGGATCTGCTAGACCTGATCTGGGGAGAAGCATCAGAGGCAAGGC<br>TGCCTAGTGAGCTGGTGACCGCGTACGTGTACAAGGTGGTGAGTGAGTGGAAAGAG<br>ATGCTGGAGAGTGATTGGTCATCTTTCCGATTCCAGATTGGCAAAGCGGGAGAGGA<br>GATAACCCCATTCAACCTGATTGGCATGACTGTGAACGGTCAGAAACTAGCTGACT<br>ACAAAAAAGCAGTTGCTCCTGAGGGGATAGATGAGGTGGCAATGGTGATCTATCTC<br>CTGGCACCATACCGGATAGTCGGAATTAAAAACGAAGACTATCAAGATCGGGTGA<br>TCACCAACATCCAGAACCAGCTGGACAGTCTCGGCGCAAAGAAGCTGCAGGTGAA<br>GGCACTGAAGAATGTCACTACTCTCATCAACAGTGCCAACTATCTGAAAATGGTGG<br>CAGTGATTGACATGTTCTACTACCATTTCAAGAACAGTCAAGAAAGAGCTGTTGTC<br>AGGATTGCCACTCTGAGTTCCCGTCACAAAGACTGTGCAGCTCTATCCACCCTGAAT<br>CACATCACCAGCTTCACTGGGAGGAGTTTTGTGCAGGTGTTGGATTGGGTTTTCACA<br>GATCAAGTTGCCAAGGAGATCGGCAGGATGATGCGTGCGGGTCAAGAAATTGATC<br>GTCCTGAATCCTACATGCCGTACCTGAAGGACTTGGGGCTGAGCAGAAAATCCCCC<br>TACTCCTCCTCAGCAAATCCTGGCACTCACTGCTGGGCACAGATGGTATGTGCCATG<br>ATGGGATCCAAAAGATCTCAAAACGCAATCGCCAGCACTGAAGAGAATCTCAGCA<br>ATCTGACAAGAAATGCTGAGATTATGGCCTATGCCCTGGGAGTGGGAGCAGACCTG<br>GTGAAGGGACTGATCATTGGAGATCAGAAGGAAGGAGACTCCGGTGTCATCCAGG<br>ATGAGGAAGGAATGGATGAGCCCAACAACATGGAGGCCCAAGATTGGCTGGAGTA<br>CATGGCCTCAAAGGGATTCAAGCTGACTCCAAATATGGAGCTGCAAGTCCGCCACA<br>TGTGCCTGCGAATCACCAATCCCAGAAAAGCCACTCTGGGGAGTTACCTGCGGGAG<br>AGATACAGTTCATCCCTGTGAGTTGAAGCAGAAATGAGTCATTTTTAGAACTATTAT<br>ATTTTTATCCTAATAGCCTTAAATAGTTAGTCATCTTGTGCTTAGATATTATGCCCTA<br>GTTATATGAAAAAAACTTTAACAGGGATAAACATAGTTTCTACGGCTCTATTTTATC<br>TCTCACATCTTAATTAACATGTTGTCATCTAACAAAAACAAAAACAAATTCGACTTA<br>GAGGGGATACAATTGCTGGCAAAAGGAGTCAAAAATGCAGGAGAATCCATAGATA<br>AAGCCACAGATGAGAAGCCTGAACATCTCTCTGACGCATTCTCAAAATACACAGA<br>ATATTTGTCCAATGAGAGCAAGGGGGAGGAGGAGGATTTCCAATTCGAACAGGTG<br>GACTATGGATTTCAAGAGTCACCTGAATCCAATAACCCTCTGCAAGAGAGCTGGAT<br>AGCGAAAGATGACCTGGAACCAGATATGGGAAAGTCCTGGGTAGAGTATCAAGCT<br>AAGATGTCCTTTGATTACAGTGAGCAGGTGAAACCCACTGTGATGAGGGAGATCAA<br>CGGCTTGCTGGGTATGCTGGGTGGATTTGCTAAATTCCAAGATGGAAAGAAAGAAT<br>ATCTTTTCTATCTTCCAGAGAAGAAAGAGTCAGAAGAGAGAAGGTCTGATAAGAA<br>ACAATGTCCTTTCAAGGTTGATGTCACCCCAAAACAAGACCCAAAAATCACTCCCA<br>CACCTGAGAAAGAGCCAAAACATCTGCCCACATCAGCTGCCGGCAAAGAAATCAC<br>CGAAGAAGCAGCCGTGATGCAAGGATTCTGGATGGACGGAATGAGGCTGACAGAG<br>AAGACATCAGGGAAGTATTGCCTGTTCTTCCCACAAAAAATGGGATGGTCTCAAGC<br>CGAATGGATCAGCAAATCAGAAGACATTAATCCCCGAACTCTGGCACATGACATCT<br>TCAAATGGATGGTATCCAAATCTCCGAAACGGGCCACATACTTGAGGAAATACATG<br>GTAGAAGAGTAACCACAGGTCTGAACCTCTTGACTATTTTTGATGCCTTAAATTGGC<br>TTGACGTACACGTATTACTTATTACATATCTCAGTTATTTATATGAAAAAAATGTTA<br>ACAGACGTCAATAACATTTGTTATATGCGCTATCATCGTGAGACTATATTCAAACGT<br>CGAATCAATTGAAGTTAAATGGCTCTATCTATATTTAAGAAGAAGGGGAAGAAGAC	JX827265.1 Eel virus European X, partial genome<br>[53]

	AGAATCTCCCAAGATGTTTCTAATGGATGCAAACCAGATGACCCCGAGTGCTCCAC<br>CCCCTTACCAGGCAGAACCTGGTCCGTTCGACACTTGGGGGAATGAGGAACTAGAA<br>GAAGCTATGAAAGTGTGCTACTTAGTAGACACCTGCTTGTCTGTGACAACAAGAGA<br>ACCTATTCGATCAGTGGTGGATGCCTATATCATAGCACAAGGAGTCTTAGATCATTA<br>TACCGGCCCGATCCTTACCCGTCCGTTTTACATTGCCTTGTTCTTGGGCGGGATCCAC<br>GGGATGCAGGCGGGAGTTAAAGGAGCGAGAAGCATCCGGTATGAAAGAGAACAT<br>CATGGACCTCTGGTGTTCCCATATCACAGAAGCAACCCGCTAGATTGGACTCCCCG<br>GGCTATTGAATTCCAGTACACCACTAGTCTGCGGGGGAAGCCGGTAGAGGTGAGCT<br>TCCAGGCCCGTCTGCAAGCTACCAGACAATTTGGACCTGGCGTAGAGGTTTATCTG<br>GATGGCCTGCAAAGGATAGAACGCCCTAGCAACGAACTAGTTCTAAAGCAATTCA<br>GAGTCCCCTTGCTGATGCTGGAGAAAGGGAAATGGGTGTTCAATCTTGATTCTGAG<br>GGCAGTTACAATTAAAAGGGTTCAACAATCACATCATCTGAGCCACTTTTGCTATA<br>ATTATACGGTCAAGTTTACCGCCACAAAAGGGTTGAGTAGCATGTAGACACATATA<br>AATGCATCATATTATTAATTAATTAATTGTTAAGTTATATGAAAAAAACTAACAGTG<br>ATACACCATTGTTTTGAGACATTTGTCACTGTGAAATTCAGTTGGTTCATTAGAGAC<br>ATGGATACTCTGATTAAAATTCTGCTGATTATAGTCATTCTAAAGTCTCTCCATGCTC<br>ACATCGAATTCGTGCCACATGATCTGAGTAAATGGAGAGACATCAGCATAGAGCAT<br>CTGGATTGTCCAATCTATGGAGATCTGTCTAATCAAGCAACTAGCACAACCCCTGTG<br>AAATACAGCAGTGTTCAATGGGGCCTAAAAAATAACATTGATGGGTACCTTTGTAT<br>ATCTGCCAAATGGTCGGTCACTTGTGATTATAGGTGGTATGGCTCCAAATACATTTC<br>AACCTCAATTGAATATGTTCCAACCAAAGAATCAGAATGCAGAGATGCCATCAAGT<br>CATCAAAAAATGGAGAGTTAGTTAGTCCCCATTTCATGCCCGAAAATTGTGGATGG<br>AACAATGTGTTAACGGAGAGTGTGACATACACAACTGTATCATCTCACGAGGTAAA<br>ATTAGATCCATACCAGATGACTTTCGTGGATTCACTCTTTCCGGGCGGTAAATGCTC<br>CTCTTCAGTGTGTAGCACCATTTATCATCAAGGAGTCTGGATAAACCCGAGTAACA<br>ACTTAGGGTTTTGCAAAGATCCGGTCGATCATCAAGGTCAGTTGTACATGGCTGGTC<br>TTGTTGGAGCTCACGGAGAGATTGTGAAGGAAGTTTGGAATCTCAGATCTGTGTTCA<br>AGCCAGAAATTGGTAGATCCAAGCATTTGACTGGATCTTGTTGGATGACATACTGTG<br>ATCAAAGAGGACTGAGATTTTCAGATGGAGAATGGGCAGGTTTCCAAATTCCAGAG<br>ATCTCAGCACTGAAACAGGTTCTGCTAGGACTTCCTGAGTGCAAAGATGATGTGCT<br>GGTTCATGCTCATGACACGAACACTGAGTTGAGGGAAATTTTGGAACACATGGACG<br>AGTCAGCATTGAATGCCATCTGCCAACAAGAAGTCCGCAGGGCAAAAGAGAGAGG<br>AGTGGTTTCTGATTGGCTATTGAGTATGATGACACCATTCACAGAGGGGTTGGGGCC<br>AGTTTATCGGCTGAACAAAGGAAAACTAGAAGCATCAATGGGATATTATAGAAAG<br>GTATATATAGACTCTAGCAACGCCCCTCAGGCCTTCGGACAGACAGAAGATAAAG<br>AATCTGTTGGATGGTCGGATCTTGTACCAAAAGATGCAGATGGAGCTATATCATCG<br>ATGTACAATGGGAATGTCGTCATCAATAATCAGATAAAATGGGCTAAAAATGCACT<br>AGGATCTCATATATTAGATGAGATTTCTGCCTTAGAATTTGAAACCCCCGTTGTGCA<br>TCATCCTCATTTGACAATCTTGTCGGTAAATCATAGTGATCTAGTGAGTTCCACACA<br>TCCAAATGGGCAAGGAGTTAATCTGATTGAGAGTGTATCTCACTGGGCCGGTGGGT<br>TGTGGGCATCCATAGGATCTGGGTTGATGATCTTGGTTCTAGTGGCTTTAGTCGGATT<br>TTGTACCATCAAGGTATGCTTAGCTTATGTTCCATCGATTTGGGCACAAAAAAATCG<br>GAATGGTAAGAGAAGAGGAACAACATCCCAACGATCAACAGAACAGGAGATGTT<br>CGAGCTATCAGCTGTGTAGGTACAAGTGATACTTCAGGTTAAATCGGAACTCTCATT<br>TCTGTGTAATCAGATCTATATATTAAAAGACCTACTTAAAATATTGCATGTGTTACT<br>GATGTCAGCGTAATAAGCTGAATCATAATTTACTTCTCATGACAATTTTGTTAACCT<br>ACATGTTTTCATCTTATTACATTTTATTCTTATTTTTATTTTTATGAATATATATGAAA<br>AAAACTATTCAACAGTCATCATGTATGACGAAGATCATTCAAGAGGATATGAATCC<br>GATGACCATTATGACCTGCCTGAATGGCTGGAAGAGGATATACAATCAGGAAATCC<br>ACTCAATCAAAAAGACTATAGCTTAAATTCTCCTCTCATTGTAGATCTCACAGAAGC<br>GCTGGTAAAATATCTAAATCAAGGTAATATAGAGAGACGATTCCTCAGACATCAAG<br>ACAGATTTGAGAACATACAAAGTGAAATCCGCAACATTGCATGGAAGAATCCATC<br>GAGCTCAAATCACAGATGGTGGGGGAAGTGGGCTCAACAAACAGAAAAGAGTCCA<br>GAGTTTGTAAGGCTGTTGTCTGATGTTAACCAGGACATAGAAGAGACTAGTGACGT<br>CTTGATGTCATTCTTGAAAGGATGGATCCAAGACACAACCTCTGTTCCAACAAAATT<br>AAATTGGACATCTACTCAATTGAGCTATGGATCCAAATTCTTCTTCATGCACAAATT<br>GATCCTGTTCATGAATGCGCAATCCGATGAGGAGAGGGTTATTCTCCAAGGGCACA<br>TCAAAGTCTCGGAAACAAAAAAGACTGGGATCTACAAAGGTTCTCATCCTAGCTTA<br>GGAAATTTTGTGCTGACCTCTGAATTCTTACTACTAGAACGACACAGAGTGATACTA<br>GACCGTTCTTTCCTGTTGATGGTTAAAGATACTCTAGTAGGGAGATTTCAGACACTC<br>GCTAGCTTCATGAATAGGGAAGACAAAAAATATCCAGAAGACGTGATAGAAAAGG<br>TGGAGACCCTCTATTCCTTAGGAGATCAATTAGTAGAGGATCTAGGGGATGAAGCA<br>TATTCGGGGATAAAGTTGCTAGAACCAGCCTGCAACCTCCGACTCGCAGAGTTGGC<br>TAGAGAATTTAGGCCTTTGATCCCCGAATTCCCACACTTTCGAAACCATGTAGAGAC<br>AGCAATAGCAGAGGAGTCTGTCTTTAGTCCTGGAATCACAGAATTTTTCAACCATGT<br>AAATAAAGAAACAAATGTTGAAATCATCTTGGCCTACTTCAGCTCTTTCCGACATTG<br>GGGACATCCATACATTGATTATTTCCAAGGGTTAATCAAATTGAACAAACAAGTCA<br>CTCTAGAAAAAGACATTGATACAGAATACGCTAATGCACTGGCTAGTGATTTGGCC<br>TACATGATTCTTCGAGGACATTTTAACACTAAAAGAGTCTGGGCGGTAGACAAATC<br>ACTTGTGTCAAAGCAACACCCGTTGAGTGAGCATATTTTCAATGCCACTTGGCCAAC<br>TCCGAAACAGATTGATGACTTCGGAGATCACTGGCATGAATTGCCTCTGATCAAAA<br>TTTATGACATCCCAGATTTAATTGACCCATCTGTCATCTACTCTGACAAAAGTCATTC<br>AATGGGGAGAGAAGAAGTGTTAAAACATGTTCAAAGGAATCCAACACAAGCAATT<br>CCTACAAAAAAGGTCTTAGAGACACTCTTACAAAAACCTGCCACAAATTGGCCAG<br>AATTCTTGTCCTCGATTGAAAAAGATGGCCTACCTAAAGACAGCTTGATTATCGGAC<br>TGAAGGGAAAAGAGAGGGAACTAAAAAAAGCTGGAAGGTTCTTTTCTCTAATGTCC<br>TGGGAATTGAGGGAGTATTTTGTAATAACTGAATACCTGATCAAAACTCATTACGTC<br>CCTCTCTTTAAAGGATTGACCATGGCAGATGACATGACAGAGGTCGTCAAGAAGAT<br>GTTAGAAAGAAGTCAAGGACAAGGAGAGGATGATTACGAGCATGTCAGCATTGCC<br>AACCACATTGATTATGAAAAATGGAACAACCATCAAAGGAAAGAGTCTAACGGCC	

	CAGTGTTCAGAGTCATGGGACAATTTCTGGGGTACCCTAGTTTGATAGAAAAGACC<br>CATGACTTCTTTGAGCAAAGCTTGATCTATTATAATGGCAGACCTGACTTGATGCAG<br>ACAGACGGAGATGAGTTGCAAAACCGAACGGAAGCTTTGGTGTGTTGGAATGGTC<br>AGAAAGGAGGATTAGAAGGACTCAGGCAAAAAGGATGGAGCATCCTCAATCTGTT<br>GGTCATCAAAAGAGAATCTAAAATCAGAAACACTAAAGTGCAAACTTTGGCCCAG<br>GGAGACAATCAGGTAGTGTGTACCCAATACCGCATTATGCCAACCAGATCGACACT<br>TGAATTGCAGGCAGAACTTGAAAAAGTTAAGAAGAACAATCAAGTTATTATGGATG<br>CTATTGAGACAGGGACTAACAAATTAGGGCTACTGATAAACAATGATGAAACCATT<br>CAATCCGCTGATTTCCTAACATATGGTAAGGTTCCAATATTTAGAGGTAATATTCGC<br>TGTTTGGAAACTAAAAGATGGTCTAGAGTGACTTGTGTCACTAATGATCAATTGCCA<br>TCCCTTTCCAATGTCATGTCATCTGTGTCGACAAACAGTCTGACGGTCTCGCATTTCG<br>ATGTCAGTCCTATAGAATCCATGAGACAATACCTGTTCTTCGGAAATTTTGCTCGGA<br>GATTGGTGGAATTCCATAATCCTGCGATGAGAGTGCCGATTAGTCTAGAAGACCTG<br>GATTCCAAACAAAAGTCTGTTTATCTGAACGCAGTGCTTTTCTTGGACCCATCTCTG<br>GGTGGCGTGTCTGGTATGTCATTGTCAAGATTCTTGACTAGGATGTTTCCTGATCCCA<br>TCACAGAAGGATTATCTTTTTGGAAAATCGTTTACGAACATACCACTTCCAAAGAC<br>ACTCAACTGCTCTGCAGAATCGCTGGAAGCCCCGAATTGGCTAGACGCCAGAACAA<br>TCTGGACAAGTTGATAGAGAATCCAACAGCATTGAATCTGAGCAAAGAAACTTCTG<br>CCCTAAGTGTCATTAAGAAAGAAGTCAGGTCACGCCTGTACAAGGATTGCGACAA<br>ATTCAAGAACAAATTGATTGCAGATGCAATTGGGATTGCTAGAGATGAAGAGGCTC<br>ACCTGGAATTATTCTTAATGTCAATCCGACCATTATTTCCGAGATTCCTGGCAGAGT<br>TCAAAGCAGCAACATTTGTTGGCATCACAGAATCTCTCATTTCTTTATTCCAAAATT<br>CCAAAACCATTCGCAACATCTTCAGGAAGAAATATGCGAAAGAATTGGAACTTAG<br>AGTAGTGCAGTGTGAATACAGATCCATCAATTTGATGTTGAGCCTCGCAGATCGAT<br>CCCACTTAGATGAAATGTGGACATGCTCGGCATCTAAAGCAGACGAACTCCGAACC<br>TTATCATGGGGGACTACAATAATTGGAACAACTGTCCCTCATCCTTTAGAGATGATC<br>AATCATGCTCATATAGGACAGAAATGCGACTCGTTAGAAACCCTGGACTACATAAA<br>TGTCACTGTGGTTCAAGATCTGACTGATTGTCTAACTAGCAAAGGGAAGTTACCAG<br>CGTACTTAGGATCCAAAACATCAGAGACTACAAGCATCTTGCAACCATGGGAAAA<br>GGAGACAAAGATTCCTGTGATTCGTAGAGCTGCTAAATTGAGAGCTGCAATCACCT<br>GGTTTGTAGAGCCTGATTCTCTTTTGGCACAAAGCATCCTGAACAACATTGAAAGTC<br>TGACTGGAGAAGATTGGTCTGCTTCAATATCTGGATTCAAAAGGACAGGATCCGCA<br>CTACATCGCTTCACTAGTGCTAGAGTGAGTGCAGGAGGCTTTTCAGCTCAAAGCCC<br>GGCCAGATTGACCAGGATGATGGCAACAACAGATACTTTCCGAGAAATTGGGTCA<br>GATAATTACGATTTTATGTTCCAATCATTACTACTCTTTGCCCAAATGACTACAGGTG<br>AGATTTACAAAAGGAGTCCAGCTACTAATTTCCACTTTCATCTGAGTTGCCACCAAT<br>GTCTTCGTAAGATCGAAGAGCCTACCTTAAACTCTGATTTTGCCTACAACCCTATTC<br>AGAGATCGGACATCTTGGATAAGTGGAAACCTCAGACCACAGATTGGTCTTCAGAA<br>AGAAAGGCTCCGGAGATAGAGGAAGGGAATTGGGACAGATTGACACATCAGGAG<br>CAAAGTTTTCAGATAGGGAAGTCCATCGGGTTTCTGTTTGGAGATTTGACAATGACA<br>AAGAATTCACATGCTCAGGATTCTTCTATTTTCCCTTTATCAATTCAATACAAGATCA<br>CAGCTGCGGAGTTTCTGGAAGGAATTCTAGATGGGATAGTCAAGGCTTCTGCTTTGT<br>CTACTATCCACCGGAGGAACTTCGATCATCACAGCAAGTACAAGTCCACTGTTTCA<br>GGAACTGTTGATTATTTAATCGAATTGATTTCAGAGTCCGCGGGGTTCACAAATCTG<br>ACCAGGAATGGTCCCTTGAAGGCTTGCTTACTGACCATTCCACATAAGATACCTCCA<br>TCCTATCCTCTCAGCCAATCTGATCTTGGGCTATGTCAAGAAACTACCTCAGACTGT<br>TACACCGAAGAATGTCTACCGGCACCTACAAGACCAGGTGGCCGACAAATTGGAT<br>ATTCTCTGATATGATGAGCCCTAACATAATCTATCCATTTGTGATCAGTGTCTCCTGC<br>GTTGGACTGGCTTACTCATCATCTTGGACAAAGAAATCTGCTGACAAATTGAGAGG<br>GTTAAGAGGAGTAGCTGAGTTGATTCGATCATCAGATGATGTGCAATTGCCTGTTGG<br>GAAGCTATTCAAAACAGTAAACCAAGAAATTAGACATGCCATAAAACATCATGCA<br>TCGGACGATGCTGAAATCCCCGAAAGCCACATCCAAGAAGGCTGGAAAAAAGAGC<br>TCATAGTCAACATTAACATGCAGCCAATTGACTATAGTAGAACAGCGACAGCGAA<br>GTCGTTAGACCGGCCTGCTCAAATTCGAGACCCTTTAATATCTGGACTAAGAACAG<br>CACAATTGGCCACTGGATCCCACTACAAGTTACGATCCATACTAGTGCAAAACCGA<br>ATCCAAGTTACCGATGCTCTGTGCGGAGGGGATGGATCTGGTGGAATCGGGGCTTG<br>TGTCCTGAGACAATACCCGTTTGCTAAGCTGATTTACAACAGTCTATTCGAGATTCA<br>AGATCTGGATATGAGGGGGAGTGCACCTGGACCGCCATCTGCTATTGCCGCAATGG<br>GGAACATGAGTATGAGGTGTGTCAATCGAGATTCTGCATGGAAAAATCCATCTGAT<br>CTGAGCCACACTTCCACCTGGGAATATTTTCAATCGTTGATGACCCAACATCAGTTG<br>CGGTGCAATCTGTGGACCTTTGACATGGAGGTTCGCAGCCATGACATTTCAGATGCT<br>ATAGAAAAGCAAATAGTGGCCAATCTTCATTTGCTACCAAAAAATGGGACAATCAT<br>ATACAAGACATATCTAACGAAATTGTCTGACATGGAGACAACAATCTTGGACAGAC<br>TGGGTGGATTCTTCAAAAGAGTCAGTCTTGTGTCTACAGACGCTACATCAAGCCATA<br>GCTCTGAAGTCTACGCTGTATTCCAAAACAAACTGGATAAGAGACAATTGGAAATC<br>CACCCCAACTGGTCTTCCTGCAATTTAGGTTCTGACATACACCCATGCTGGAAAAGT<br>GAAGATGAGGAGTTTGAAAGGGCAAGAAGATTCTTTCACATGAAGAGACAACAAG<br>GGGTGCCGACTCGATTGAGACCTACTTTGGATTCCGAAATCCAGGTCCTGAGCGTCT<br>CGGCCGGAGTTGAAAACGGGGTAGCAATGACATTAGCTATGGACGTAAGCAATCA<br>GGTCTCGGATCCAACCACTGGGGCATTTCTTTGGCTTTTAGTAACTTTGCAACATAT<br>ATGCCCAATAGGCCCATCATTCAAGATCCCATCCAGTTCTGCAGTTGAATCTTATTT<br>GGCCATACTGATTGGATTCAGTAGTATCTACCAGCTTCAGACAGGCAATAACAAAG<br>CTTATGCCAGCATCAAGACCTGCTTGTCTCAATCTGCTCCATTCTTCTGCAACCCTGA<br>TGGATGGAGTTGCATTAAAGGACTTGACAAGTCTATGCGGATGGACAGAAAGTTGG<br>CTTTAGTGGGCAGTGTGATACGGGCATGGTCAAAGTGGAACTTGACTCAGTCAATC<br>AACTTTCACAAACTAGATGGTATGATGAGACATTATCTACCGAAGGGAACATTGAG<br>GCAGATTGGCTGCAAAACCGGGATCTGGGACTATATCAATGGGGCAGTGAAAGGA<br>GTACGAAGTGCTGCCAATCAGGACAGTCCAAAAGAGTCAGCAGCTGCTTGGAGAG<br>ATTAGTATATATAACATTGTGTCTATCATCCAATTCATTATTAGATAGGTGACAATT<br>AGTTAAAGAACAGATTACCCCATGTAAGAGAATTTTTTATGTCCTAATTTGCCGTAT	

	GCACTTGTAAATAAATGTGTATTTTAATGTATATGTGTATGTGTATTTGATGTATTGG<br>TGTATATGTCAACAAATCATAATATGATCACATAGGATAAGGTCAAGACCAATTGT<br>TTAAGTTTTTATTAGAATTCCGTGAATATGAAAAAAAACAAAACAGGTCCTTTTTGG<br>ATAAATCCTTAATAATGGGGG	
KC608033	TAAGGCTATTCTTTAACAGACATCTGTTTGTTTCATTGAAAATGGCTGCCATGAACT<br>CCATCAAAGTTACAGTTAGAGCCAATGGACAAGATTTCACCCCAGTTTGTCCTAAA<br>ATGGAGGATGAAGTGGCTTACCCATCTGATTATTTAGATGCTAATGGATTACCAACC<br>TTCCAGCTCTACTATCATGATCTGTCAAAAAAGGATCTGCTAGACCTGATCTGGGGA<br>GAAGCATCAGAGGCAAGGCTGCCTAGTGAGCTGGTGACTGCGTACGTGTACAAAGT<br>GGTGAGTGAGTGGAAAGAGAAGCTGGAGAGTGATTGGTCATCTTTCCGATTCCAGA<br>TTGGCAAAGCGGGAGAGGAGATAACCCCATTCAACCTGATTGGAATGACTGTGAA<br>CAGTCAGAAACTAGCTGACTACAAAAAAGCAGTTGCTCCTGAGGGGATAGATGAG<br>GTGGCAATGGTGATCTATCTCCTGGCACCATACCGGATAGTCGGAATTAAAAACGA<br>AGACTATCAAGATCGGGTGATCACCAACATCCAGAACCAGCTAGACAGTCTCGGC<br>GCAAAAAAGCTGCAGGTGAAGGCACTGAAGAATGTCACTACTCTCATCAACAGTG<br>CCAACTATCTGAGAATGGTGGCAGTGATTGACATGTTCTACTACCATTTCAAGAACA<br>GTCAAGAAAGAGCTGTTGTCAGGATTGCCACTCTGAGTTCCCGTCACAAAGACTGT<br>GCAGCTTTATCCACTCTGAATCACATCACCAGCTTCACTGGGAGGAGTTTTGTGCAG<br>GTGTTGGATTGGGTTTTCACAGATCAAGTTGCCAAGGAGATCGGCAGGATGATGCG<br>TGCGGGTCAAGAAATTGATCGTCCTGAATCCTACATGCCGTACCTGAAGGACTTGG<br>GGCTGAGCAGAAAATCCCCCTACTCCTCCTCAGCAAATCCTGGCACTCACTGCTGG<br>GCACAGATGGTATGTGCCATGATGGGATCCAAAAGATCTCAAAACGCAATCGCCA<br>GCACTGAAGAGAATCTCAGCAATCTGACAAGAAATGCTGAGATTATGGCCTATGCC<br>CTGGGAGTGGGAGCAGACCTGGTGAAGGGACTGATCATTGGAGATCAGAAAGAAG<br>GAGACTCCGGTGTCATCCAGGATGAGGAAGGAATGGATGAGCCCAACAACATGGA<br>GGCCCAAGATTGGCTGGAGTACATGGCCTCAAAGGGATTCAAGCTGACTCCAAATA<br>TGGAGCTGCAAGTCCGCCACATGTGCCTGCGAATCACCAATCCCAGAAAAGCCACT<br>CTGGGGAGTTACCTGCGGGAGAGATACAGTTCATCCCTGTGAGTTGAAGCAGAAAT<br>GAGTCATTTTTAGAACTATTATATTTTTATCCCAATAGCCTTAAATAGCTAGTCATCC<br>TGTGCTTAGATATTATGCCCTAGTTATATGAAAAAAACTTTAACAGAGATAAACAT<br>AGTTTCTACGGCTCTATTTTATCTCTCACATCTTAATTAACATGTTGTCATCTAACAA<br>AAACAAAAACAAATTCGACTTAGAGGGGATACAATTGCTGGCAAAAGGAGTCAAA<br>AATGCAGGGGAATCCATAGATAAAGCCACAGATGAGAAGCCTGAACATCTCTCTG<br>ACGCATTCTCAAAATACACAGAATATTTGTCAAATGAGAGCAAGGGGGATGAGGA<br>GGATTTCCAATTTGAACAGGTGGACTATGGATTTCAAGAGTCACCTGAATCCAATA<br>ACCCTCTGCAAGAGAGCTGGGTAGCGAAAGATGACCTGGAACCAGATATGGGAAA<br>GTCCTGGGTAGAGTATCAAGCTAAGATGTCTTTTGATTACAATGAGCAGGTGAAAC<br>CCACTGTGATGAGGGAGATCAACGGCTTGCTGGGTATGCTGGGTGGATTTGCTAAA<br>TTCCAAGATGGAAGGAAAGAATATCTTTTCTATCTTCCAGAGAAGAAAGAGTCAGA<br>AGAGAGAAGGTCTGATAAGAAACAATGTCCTTTCAAGGTTGATGTCACCCCAAAAC<br>AAGACCCAAAAATCACTCCCACACCTGAGAAAGAGCCAAAACATCTGCCCACATC<br>AGCTGCCGGCAAAGAAATCACCGAAGAAGCAGCCGTGATGCAAGGATTCTGGACG<br>GACGGAATGAGGCTGACAGAGAAGACATCAGGGAAGTATTGCCTGTTCTTCCCACA<br>AAAAATGGGATGGTCTCAAGCCGAATGGATCAGCAAATCAGAAGACATTAATCCC<br>CGAACTCTGGCACATGACATCTTCAAATGGATGGTATCCAAATCTCCGAAACGGGC<br>CACATACTTGAGGAAATACATGGTAGAAGAGTAACCACAGGTCTGAATCTCTTGAC<br>TATTTTTGATGCCTTAAATTGGCTTGACGTACACGTATTACTTATTACATATCTCAGT<br>TATTTATATGAAAAAAATGTTAACAGACGTCAATAACATTTGTTATATGCGCTATCA<br>TCGTGAGACTATATTCAAACATCGAATCAATTGAGGTTAAATGGCTCTATCTATATT<br>TAAGAAGAAGGGGAAGAAGACAGAATCTCCCAAGATGTTTCTAATGGATGCAAAC<br>CAGATGACCCCGAGTGCTCCACCCCCTTACCAGGCAGAACCTGGTCCGTTCGACAC<br>TTGGGGGAATGATGAGCTAGAAGAAGCTATGAAAGTGTGCTACTTAGTAGACACTT<br>GCTTGTCTGTGACAACAAGAGAACCTATTCGATCAGTGGTGGATGCCTATATCATA<br>GCACAAGGAGTCTTGGATCATTATACCGGCCCGATCCTTACCCGTCCGTTTTACATT<br>GCCTTGTTCTTGGGCGGGATCCACGGGATGCATGCGGGATTTAAAGGAGCGAGAAG<br>CATCCGGTATGAAAGAGAACATCATGGACCTCTGGTGTTCCCATATCACAGAAGCA<br>ACCCGCTAGATTGGACTCCCCGGGCTATTGAATTCCAGTACACCACTATTCTGCGGG<br>GGAAGCCGGTAGAGGTGAACTTCCAAGCCCGTCTGCAAGCTACCAGACAATTTGGA<br>CCTGGAGTAGAGGTTTATCTGGATGGCCTGCAAAGGATAAAACGCCCTAGCAACGA<br>ACTATTTCTAAAGCAATTCAGAGTCCCCCTGCTGATGCTGGAGAAAGGGAAATGGG<br>TGTTCAATCTTGATTCTGAGGGCAGTTACAATTGAAAGGATTCAACAATCACATCAT<br>CTGAGCCACTTTTGCTATAATTATACAGTCAAGTTTACCGCCACAAAAGGGTTGAGT<br>AGCATGTAGACACATATAAATGCATCATATTATTAATTAATTAATTGTTAAGTTCTA<br>TGAAAAAAACTAACAGTGATACACCATTGTTTTGAGACATTTGTTACTGTGAAATTC<br>AGTTGGTTCAATAGAGACATGGATACTCTGATTAAAATTCTGCTGATTATAGTCATT<br>CTAAAGTCTCTCCATGCTCACATCGAATTCGTGCCACATGATCTGAGCAAATGGAG<br>AGACATCAGCATTGAGCATCTGGATTGTCCAATCTATGGAGATCTGTCTAATCAAGC<br>AACTAGAACAACCCCTGTGAAATACAGCAGTGTTCAATGGGGCCTAAAAAATAAC<br>ATTGATGGGTACCTTTGTATATCTGCCAAATGGTCGGTCACTTGTGATTATAGGTGGT<br>ATGGCTCCAAATACATTTCAACCTCAATTGAATATGTTCCAACCAAAGAATCAGAA<br>TGCAGAGATGCCATCAAGTCATCAAAAAATGAAGAGTTAGTTAGTCCTCATTTCAT<br>GCCCGAAAATTGTGGATGGAACAATGTGTTAACGGAGAGTGTGACATACACAACTG<br>TATCATCTCACGAGGTAAAATTAGATCCATACCAGATGACTTTCGTGGATTCACTCT<br>TTCCGGGCGGTAAATGCTCCTCTTCAGTGTGTAGCACCATTTATCATCAAGGAGTCT<br>GGATAAACCCTAGTAACAATTTAGGATTTTGCAAAGATCCGGTCGATCATCAAGGT<br>CAGTTGTACATGGCTGGTCTTGTTGGAGCTCACGGAGAGATTGTGAAGGAAGTTTG<br>GAATCTCAGATCTGTGTTCAAGCCAGAGATTGGTAGATCCAAGCATTTGACTGGAT<br>CTTGTTGGATGACATACTGTGATCAAAGAGGACTGAGATTTTCAGATGGAGAATGG<br>GCAGGTTTCCAAATTCCAGAGATCTCAGCACTGAAACAGGTTCTGCTAGGACTTCCT<br>GAGTGCAAAGATGATGTGCTGGTTCATGCTCATGACACGAACACTGAGTTGAGGGA	KC608033.1 Eel virus European X isolate GG184, partial genome [54]

	AATTTTGGAACACATGGACGAGTCAGCATTGAATGCCATCTGCCAACAAGAAGTCC	
	GCAGGGCAAAAGAGAGAGGAGTGGTTTCCGATTGGCTATTGAGTATGATGACACC	
	ATTCACAGAGGGGTTGGGGCCAGTTTATCGGCTGAACAAAGGAAAACTAGAAGCA	
	TCAATGGGATATTATAGAAAGGTATATATAGACTCCAGCAACGCCCCTCAGGCCTT	
	CGGACAGACAGAAGACAAAGAATCTGTTGGATGGTCGGATCTTGTACCAAAAGAT	
	GCAGATGGAGCTATATCATCGATGTACAATGGGAATGTCGTCATCAATAATCAGAT	
	AAAATGGGCTAAAAATGCACTAGGATCTCATATATTAGATGAGATTTCTGCCTTAG	
	AATTTGAAACCCCCATTGTGCATCATCCTCATTTGACAATCTTGTCGGTAAATCATA	
	GTGATCTAGTGAGTTCCACACATCCAAATGGGCAAGGAGTTAATCTGATTGAAAGT	
	GTATCTCACTGGGCCGGTGGGTTGTGGGCATCCATAGGATCTGGGTTGATGATCTTG	
	GTTCTAGTGGCCTTAGTCGGATTTTGTACCATCAAGGTCTGCTTAGCTTATGTTCCAT	
	CGATTTGGGCACAAAAAAATCAAAATGGTAAGAGAAGAGGAACAACATCCCAAC	
	GATCAACAGAACAGGAGATGTTCGAGCTATCAGCTGTGTAGGTACAAGTGATACTT	
	CAGGTTAAATCGGAACTTTCATTTCCGTGGAATCAGATCTATATATTAAAAGACCTA	
	CTTAAAATATTGCATGTGTTACTGATGTCAGCGTAATAAGCTGAATCATAATCTACT	
	TCTCATGACAATTTGGTTAACCTACATGTTTTCATCTTATTACATTTTATTCTTATTTTT	
	ATTTTATGAATATATATGAAAAAAACTGTTCAACAGTCATCATGTATGACGAAGAT	
	CATTCAAGAGGATATGAATCCGATGACCATTATGACCTGCCTGAATGGCTGGAAGA	
	GGATATACAATCAGGAAATCCACTCAATCAGAAAGACTATAGCTTAAATTCTCCTC	
	TCATTGTAGATCTCACAGAAGCGCTGATAAAATATCTAAATCAAGGTACTATAGAG	
	AGACGATTCCTCAGACATCAAGACAGATTTGAGAACATACAAAGTGAAATCCGCA	
	ACATTGCATGGAAGAATCCATCGAGCTCAAATCACAGATGGTGGGGGAAGTGGGC	
	TCAACAAACAGAAAAGAGTCCAGAGTTTGTAAGGTTGTTGTCTGATGTTAACCAAG	
	ACATAGAAGAGACTAGTGACATCTTGATGTCATTCTTGAAAGGATGGATCCAAGAC	
	ACAACCTCTGTTCCAACAAAATTAAATTGGACATCTACTCAATTGAGCTATGGATCT	
	AAATTCTTCTTCATGCACAAATTGATCCTGTTCATGAATGCGCAATCCGATGAGGAG	
	AGGGTTATTCTCCAAAGGCACATCAAAGTCTCGGAAACAAAAAAGACTGGGATCT	
	ACAAAGGTTCTCATCCTAGCTTAGGAGATTTTGTGCTGACCTCTGAATTCTTACTACT	
	AGAACGACACAGAGTGATACTAGACCGTTCTTTCCTGTTGATGGTTAAAGATACTCT	
	AGTAGGGAGATTTCAGACACTCGCTAGCTTCATGAATAGGGAAGACAAAAAATAT	
	CCAGAAGACGTGATCGAAAAGGTGGAGACCCTCTATTCCTTAGGAGATCAATTGGT	
	AGAGGATCTAGGGGATGAAGCATATTCGGGGATTAAGTTGCTAGAACCAGCCTGCA	
	ACCTCCGACTCGCAGAGTTGGCTAGAGAATTCAGGCCTTTGATCCCCGAATTCCCAC	
	ACTTTCGAAATCATGTAGAGACAGCAATAGCAGAGGAGTCTGTCTTTAATCCTGGA	
	ATCACAGAATTTTTCACCCATGTAAATAAAGAAACAAATGTTGAAATCATCTTGGC	
	CTATTTCAGCTCTTTCCGACATTGGGGACATCCATACATTGATTATCTCCAAGGGTTA	
	ATCAAATTGAACAAACAAGTCACTCTAGAAAAAGATATTGATACAGAATACGCTA	
	ATGCACTGGCTAGTGATTTGGCCTACATGATTCTTCGAGGACATTTTAACACTAAAA	
	GAGTCTGGGCAGTAGACAAATCACTTGTGTCAAAGCAACACCCGTTGAGTGAGCAT	
	ATTTTCAATGCCACTTGGCCAACTCCGAAACAGATTGATGACTTCGGAGATCACTGG	
	CATGAACTGCCTCTGATCAAAATTTATGACATCCCAGATTTAATTGACCCATCTGTC	
	ATCTACTCTGACAAAAGCCATTCAATGGGGAGAGAAGAAGTGTTAAAACATGTTCA	
	AAGGAATCCAACACAAGCAATTCCTACAAAAAAGGTCTTAGAGACACTCTTACAA	
	AAACCTGCCACAAATTGGCCAGAATTCTTGTCCTCGATTGAAAAAGATGGCCTACC	
	TAAAGACAGCTTGATTATCGGACTGAAGGGAAAAGAGAGGGAACTAAAAAAAGCT	
	GGAAGATTCTTTTCTCTAATGTCCTGGGAACTGAGGGAGTATTTTGTAATAACTGAA	
	TACCTGATCAAAACTCATTACGTCCCTCTTTTTAAAGGATTGACTATGGCAGATGAC	
	ATGACAGAGGTTGTCAAGAAGATGTTAGAAAGAAGTCAAGGACAAGGAGAGGAT	
	GATTACGAGCATGTCAGCATTGCCAACCACATTGATTATGAAAAATGGAACAACCA	
	TCAAAGGAAAGAGTCTAACGGCCCAGTGTTCAGAGTCATGGGACAATTTCTTGGGT	
	ACCCTAGTTTGATAGAGAAGACCCATGACTTCTTTGAGCAAAGCTTGATCTATTATA	
	ATGGCAGACCTGACTTAATGCAGACAGACGGAGATGAGTTGCAAAACCGAACGGA	
	AGCTTTGGTGTGTTGGAATGGTCAGAAAGGAGGATTAGAAGGACTCAGGCAAAAA	
	GGATGGAGCATCCTCAATCTGTTGGTCATCAAAAGAGAATCTAAAATCAGAAACAC	
	TAAAGTGCAAACTTTGGCCCAGGGAGACAATCAGGTAGTGTGTACCCAATACCGCA	
	TTATGCCAACCAGATCGACACTTGAATTGCAGGCAGAACTTGAAAAAGTTAAGAAG	
	AACAATCAAGTTATTATGGATGCTATTGAGACAGGGACTAACAAATTAGGGCTACT	
	GATAAACAATGATGAAACCATTCAATCCGCTGATTTCCTAACATATGGTAAGGTTC	
	CAATATTTAGAGGTAATATTCGCTGTTTGGAAACCAAAAGATGGTCTAGAGTGACTT	
	GTGTCACTAATGATCAATTGCCATCCCTTTCCAATGTCATGTCATCTGTGTCGACAA	
	ACAGTCTGACGGTCTCGCATTTCGATGTCAGTCCTATAGAATCCATGAGACAATACC	
	TGTTCTTCGGAAATTTTGCCAGGAGATTGGTGGAATTCCATAATCCTGCGATGAGAG	
	TGCCGATTAGTCTAGAAGATCTGGATTCCAAACAAAAGTCCGTTTATCTGAACGCA	
	GTGCTTTTCTTGGACCCATCTCTGGGTGGCGTGTCTGGTATGTCATTGTCAAGATTCT	
	TGACTAGGATGTTTCCTGATCCCATCACAGAAGGATTATCTTTTTGGAAAATTGTTTA	
	CGAACATACCACTTCCAAAGACACTCAACTGCTTTGCAGAATCGCTGGAAGCCCCG	
	AATTGGCTAGACGCCAGAACAATCTAGACAAGTTGATAGAGAATCCAACAGCATT	
	GAATCTGAGCAAAGAAACTTCTGCCCTAAGTGTCATTAAGAAAGAAGTCAGGTCAC	
	GCCTGTACAAGGATTGCGACAAATTCAAGAACAAATTGATTGCAGATGCAATTGGG	
	ATTGCTAGAGATGAAGAGGCTCACCTGGAATTATTCTTAATGTCAATCCGACCATTA	
	TTTCCGAGATTCCTGGCAGAGTTCAAAGCAGCAACATTTGTTGGCATCACAGAATCT	
	CTCATTTCTTTATTCCAAAATTCCAAAACCATTCGCAACATATTCAGGAAGAAATAT	
	GCGAAAGAATTGGAACTTAGAGTAGTGCAGTGTGAATACAGATCCATCAATTTGAT	
	GCTGAGCCTCGCAGATCAATCCCACTTAGATGAAATGTGGACATGCTCGGCATCTA	
	AAGCAGACGAACTCCGAACCTTATCATGGGGGACTACAATAATTGGAACAACTGTC	
	CCTCATCCTTTAGAGATGATCAATCATGCTCATATAGGACAGAAATGCGACTCGTTA	
	GACACCCTGGACTACATAAATGTCACTGTGGTTCAAGATTTGACTGATTGTCTAACT	
	AGCAAAGGGAAGTTACCAGCGTACTTAGGATCCAAAACATCAGAGACTACAAGCA	
	TCTTGCAACCATGGGAAAAGGAGACAAAGATTCCTGTGATTCGTAGAGCTGCTAAA	
	TTGAGAGCTGCAATCACCTGGTTTGTAGAGCCTGATTCTCTTTTGGCACAAAGCATC	

	CTGAACAACATTGAAAGTCTGACTGGAGAAGATTGGTCTGCTTCAATATCTGGATTC<br>AAAAGGACAGGATCTGCACTACATCGCTTCACTAGTGCTAGAGTGAGTGCAGGAG<br>GATTTTCAGCTCAAAGCCCGGCCAGATTGACCAGGATGATGGCGACAACAGATACT<br>TTCCGAGAAATTGGGTCAGATAATTACGATTTTATGTTCCAATCATTACTACTCTTTG<br>CCCAAATGACTACAGGTGAGATTTACAAAAGGAGTCCAGCTACTAACTTCCACTTT<br>CATCTGAGTTGCCACCAATGTCTTCGTAAGATCGAAGAGCCTACCTTAAACTCTGAT<br>TTTGCCTACAACCCTATTCAGAGATCGGACATCTTGGATAAGTGGAAACCTCAGAC<br>CACAGATTGGTCTTCAGAAAGAAAGGCTCCAGAGATAGAGGAAGGGAATTGGGAC<br>AGATTGACACATCAGGAGCAAAGTTTTCAGATAGGGAAGTCCATCGGGTTTCTGTTT<br>GGAGATTTGACAATGACAAAGAATTCACATGCCCAGGATTCTTCTATTTTCCCTTTA<br>TCAATTCAATACAAGATCACAGCTGCGGAGTTTCTGGAAGGAATTCTAGATGGGAT<br>AGTCAAGGCTTCTGCTTTGTCTACTATCCACCGGAGGAACTTCGATCATCACAGCAA<br>GTACAAGTCCACTGTTTCAGGAACTGTTGATTATTTAATCGAATTGATTTCAGAGTC<br>CGCTGGGTTCACAAATCTGACCAGGAACGGTCCCTTGAAAGCTTGCTTACTGACCA<br>TTCCACATAAGATACCTCCATCCTATCCTCTCAGCCAATCTGATCTTGGTGCTATGTC<br>AAGAAACTACCTCAGACTGTTGCACCGAAGAATGTCTACCGGCACCTACAAGACC<br>AGGTGGCCGACAAATTGGATATTCTCTGATATGATGAGCCCTAACATAATCTATCCA<br>TTTGTGATCAGTGTCTCCTGCGTTGGACTGGCTTACTCGTCATCTTGGACAAAGAAA<br>TCTGCTGACAAATTGAGAGGGTTGAGAGGAGTAGCTGAGTTGATTCGATCATCAGA<br>TGATGTGCAATTGCCTGTTGGGAAGCTATTCAAAACAGTAAACCAAGAAATTAGAC<br>ATGCCATAAAACATCATGCATCGGATGATGCAGAAATCCCCGAAAGCCACATCCA<br>AGAAGGCTGGAAAAAAGAGCTCGTAGTCAACATTAACATGCAGCCAATTGACTAT<br>AGTAGAACAGCGACAGCGAAGTCGTTGGACCGGCCTGCTCAAATTCGAGACCCTTT<br>AATATCTGGACTAAGAACAGCACAATTGGCCACTGGATCCCACTACAAGTTACGAT<br>CCATACTAGTGCAAAACCGAATCCAAGTTACCGATGCTCTGTGCGGAGGGGATGGA<br>TCTGGTGGAATCGGGGCTTGTGTCCTGAGACAATACCCGTTTGCTAAACTGATTTAC<br>AACAGTCTATTCGAGATTCAAGATCTGGATATGAGGGGGAGTGCACCTGGACCGCC<br>ATCCGCTATTGCCGCAATGGGGAACATGAGCATGAGGTGTGTCAATCGAGATTCTG<br>CATGGAAAAATCCATCTGATCTGAGCCACCCTTCCACCTGGGAATATTTTCAATCGT<br>TGATGACCCAACATCAGTTGCGGTGCAATCTGTGGACCTTTGACATGGAGGTTCGC<br>AGCCATGACATTTCAGATGCGATAGAAAAGCAAATAGTGGCCAATCTTCATTTGCT<br>ACCAAAAAATGGGACAATCATATACAAGACATATCTAACGAAATTGTCTGACATG<br>GAGACAACAATCTTAGACAGACTGGGTGGATTCTTCAAAAGAGTCAGTCTTGTGTC<br>TACAGACGCTACATCAAGCCATAGCTCTGAAGTCTACGCTGTATTCCAAAACAAAC<br>TGGATAAGAGACAATTGGAAATCCACCCCAACTGGTCTTCCTGCAATTTAGGTTCTG<br>ACATACACCCATGCTGGAAAAGTGAAGATGAGGAGTTTGAAAGGGCAAGAAGATT<br>CTTTCACATGAAGAGACAACAAGGGGTGCCGACTAGATTGAGACCCACTTTGGATT<br>CCGAAATCCAGGTCCTGAGCGTCTCGGCTGGAGTTGAAAACGGGGTAGCAATGAC<br>ATTAGCTATGGACGTAAGCAATCAGGTCTCGGATCCAACCACTGGGGCATTTCTTTG<br>GCTTTTAGTAACTTTGCAACATATATGCCCAATAGGCCCATCATTCAAGATACCATC<br>CAGTTCTGCAGTTGAATCTTATTTGGCCATACTGATTGGATTCAGTAGTATCTACCAG<br>CTTCAGACAGGCAATAACAAAGCCTATGCCAGCATCAAGACCTGCTTGTCTCAATC<br>TGCTCCATTCTTCTGCAACCCTGATGGATGGAATTGCATTAAAGGACTTGACAAGTC<br>TATGCGGATGGACAGAAAGTTGGCTTTGGTGGGCAGTGTGATACGGGCATGGTCAA<br>AGTGGAACTTGACTCAGTCGATCAACTTTCACAAATTAGATGGTATGATGAGACAT<br>TATCTACCGAAGGGAACATTGAGGCAGATTGGCTGCAAAACCGGGATCTGGGACT<br>ATATTAATGGGGCAGTGAAAGGAGTACGAAGTGCTGCCAATCAGGACAGTCCAAA<br>AGAGTCAGCAGCTGCTTGGAGAGATTAGTATATATAACATTGTGTCTCTCATCCAAT<br>TTATTATTAGATAGGTGACAATTAGTTAATGAACAAGATTACCCCATGTAATAGAAT<br>TTTTTATGTCCTAATTTGCTGTATGCACTTGTAAATAAATATGTATTTCAATGTATAT<br>GTGTATGTGTATTTGATGCATTGGTGTATATGTCAACAAATCAATAATATGATCACA<br>TAGGATAAGGTCAAGACCAATTTTTTAATTTTTATTAGAAGTCCGTAAATATGAAAA<br>AAACAAAACAGKCYTT	
KC608034	TAAGGCTATTCTTTAACAGACATCTGTTTGTTTCATTGAAAATGGCTGCCATGAATTC<br>TATCAAAGTTACAGTTAGAGCCAATGGACAAGATTTCACCCCAGTTTGTCCTAAAA<br>TGGAGGATGAAGTGGCTTACCCATCTGATTATTTAGATGCTAATGGATTACCAACTT<br>TCCAGCTCTATTATCATGATCTGTCAAAAAAGGATCTGCTAGATCTGATCTGGGGAG<br>AAGCATCAGAGGCAAGGCTGCCTAGTGAGCTGGTGACAGCGTATGTGTACAAGGT<br>GGTGAGTGAGTGGAAAGAGAAGCTGGAGAGTGATTGGTCATCTTTCCGATTCCAGA<br>TTGGCAAAGCGGGAGAGGAGATAACCCCATTCAACCTGATTGGCACGACTGTGAA<br>CGGTCAGAAACTAGCTGACTACAAAAAAGCAGTTGCTCCTGAGGGGATAGATGAG<br>GTGGCAATGGTGATCTATCTCCTGGCACCATACCGGATAGTCGGAATTAAAAACGA<br>AGACTACCAAGACCGGGTGATCACCAACATCCAGAACCAGCTGGACAGTCTCGGA<br>GCAAAGAAGCTGCAGGTAAAGGCACTGAAGAATGTCACTACTCTCATCAACAGTG<br>CCAACTATCTGAAAATGGTGGCAGTGATTGACATGTTCTACTACCATTTCAAGAACA<br>GTCAAGAAAGAGCCGTTGTCAGGATTGCCACTCTGAGTTCCCGCCACAAAGACTGT<br>GCAGCTCTATCCACCCTGAATCACATCACCAGCTTCACTGGGAGGAGTTTTGTGCAG<br>GTGTTAGATTGGGTTTTCACAGATCAAGTCGCCAAGGAGATCGGCAGGATGATGCG<br>CGCGGGTCAAGAAATTGATCGTCCTGAATCCTACATGCCGTACCTGAAGGACCTGG<br>GGCTGAGCAGAAAATCCCCCTACTCCTCATCAGCAAATCCTGGCACTCACTGCTGG<br>GCACAGATGGTGTGTGCCATGATGGGATCCAAAAGATCTCAAAACGCAATCGCCA<br>GCACTGAGGAGAATCTCAGCAATCTGACAAGAAATGCTGAGATTATGGCCTATGCC<br>CTGGGAGTGGGAGCAGACCTGGTGAAGGGACTGATCATCGGGGATCAAAAGGAAG<br>GAGATTCCGGTGTCATTCAGGATGAGGAAGGAATGGATGAGCCCAACAACATGGA<br>GGCTCAAGATTGGCTGGAGTACATGGCCTCAAAGGGATTCAAGCTGACTCCAAATA<br>TGGAGCTGCAAGTCCGCCACATGTGCCTGCGAATCACCAATCCCAGAAAAGCTACT<br>CTGGGGAGTTACCTGCGGGAGAGATACAGTTCATCCCTGTGAGTTGAAGCAGAAAT<br>GAGTCATTTTTAGAACTATTATATTTCTATCTTAATAGCCTTAAATAGTTAGTCATTC<br>TGTGCTTAGATGTCATGCCTTAGTTATATGAAAAAAACTTTAACAGGGATAAACAT<br>AGTTTCTACGGCTCTATTTTATCTCTCACATTTTAATTAACATGTTGTCATCTAACAG	KC608034.1 Eel virus European X isolate DK3545, partial genome [55]

	AAACAAAAACAAATTCGACCTAGAGGGGATACAATTGCTGGCAAAAGGAGTCAAA<br>AATGCAGGAGAATCCATAGATAAAGCCACAGATGAGAAGCCTGAACATCTCTCGG<br>ACGCATTTTCAAAATACACAGAATATTTGTCAAATGAGAGCAAGGTGGAGGAGGA<br>GGATTTCCAATTCGAACAGGTGGAATATGGATTTCAAGAATCACCTGAATCCAATA<br>ACCCTCTGCAAGAGAGCTGGATAGCGAAAGATGACCTGGAACCAGATATGGGAAA<br>GTCCTGGGTAGAGTATCAAGCTAAGATGTCCTTCGATTACAATGAACAAGTGAAAC<br>CCACTGTGATGAGAGAGATCAACGGCTTGCTGAGTATGCTGGGTGGATTTGCTAAA<br>TTCCAGGACGGAAAGAAAGAATATCTTTTCTATCTTCCAGAGAAGAAAGAGTCAGA<br>AGAGAAAAGGTCTGATAAGAAGCAATGTCCGTTCAAGGTGGATGTCACTCCAAAA<br>CAAGACCCAAAAATCACTCCCACACCTGAGAAAGAGCCAAAACACCTGCCCACAT<br>CAACTGCCAGCAAAGAAATCACCGAAGAAGCAGCCGTGATGCAAGGATTTTGGAT<br>GGACGGAATGAGGCTGACAGAGAAGACATCAGGGAAGTATTGCCTGTTCTTCCCAC<br>AGAAAATGGGATGGTCTCAAGCCGAATGGATCAGCAAATCAGAAGACATTAATTC<br>CCGAACTCTAGCACATGACATCTTCAAATGGATGGTATCCAAATCTCCGAAACGGG<br>CCACATACTTGAGGAAATACATGGTGGAAGAGTAACCACAGGTCTGAAAATCTTGA<br>CCATTTTTGATGCCTTAAATTGGCTTGACATAAACATATTACTTATTACATATCTCAG<br>TTATCTCTATGAAAAAAACGTTAACAGACGTCAATAACATTTGTTATATACGCTATC<br>ATCGTGAGACTACATTCAAACGTCGAATCAATTGAGGTTAAATGGCTCTATCTATAT<br>TTAAGAAGAAGGGGAAGAAGACAGAATCTCCCAAGATGTTTTTAATGGATGCAAA<br>TCAGATGACCCCGAGTGCTCCACCCCCTTACCAGGCAGAACCTGGTCCGTTCGACA<br>CTTGGGGGAATGAGGAACTAGAAGAAGCTATGAAAGTGTGTTACTTAGTAGACACC<br>TGCTTGTCTGTGACAACAAGAGAACCCATTCGATCAGTGGTGGATGCCTATATCATA<br>GCACAAGGAGTCTTAGATCATTATACCGGCCCGATCCTTACCCGTCCGTTTTACATT<br>GCCTTGTTCTTGGGCGGGATCCACGGGATGCAGGCGGGGGTTAAAGGAGCGAGAA<br>GCATCCGGTATGAAAGAGAACATCATGGACCTCTGGTGTTCCCATATCACAGAAGC<br>AACCCGCTAGATTGGACTCCCCGGGCCATTGAATTCCAGTACACCACTAGTCTGCG<br>GGGGAAGCCGGTAGAGGTGAGCTTCCAGGCCCGTCTGCAAGCTACCAGACAATTTG<br>GACCTGGCGTAGAGGTTTATCTGGATGGCCTGCAAAGGATAGAACGCCCTGGCAAC<br>GAACTAGTTCTAAAGCAATTCAGAGTCCCCTTGCTGATGCTGGAGAAAGGGAAATG<br>GGTGTTCAATCTTGATTCTGAGGGCAGTTACAATTAAAAGGGTTCAACAATCACATC<br>ATCTAAGTCACTTTTGCTATAATTATACGGTCAAGTTTACCGCCACAAAAGGGTTGA<br>GTAGCATGTAGACACATATAAATGCATCATATTATTAATTAATTAATTGTTAAGTTA<br>TATGAAAAAAACTAACAGTGATACACCATTGTTTTGAGACATTTGTCACTGTGAA<br>ATTCAGTTGGTTCAATAGAGACATGGATACTCTGATTAAAATTTTGCTGATTATAGT<br>CATTCTAAAGTCTCTCCATGCTCACATCGAATTCGTGCCACATGATCTGAGTAAATG<br>GAGAGACATCAGCATAGAGCATCTGGATTGTCCAATCTATGGAGATCTGTCTAATC<br>AAGCAACTAGAACAACCCCTGTGAAATACAGCAGTGTTCAATGGGGCTTAAAAAA<br>TAACATTGATGGGTACCTTTGTATATCTGCCAAATGGTCGGTCACTTGTGATTATAG<br>GTGGTATGGCTCCAAATACATTTCAACCTCAATTGAATATGTTCCAACAAAAGAAT<br>CAGAATGCAGAGATGCCATCAAGTCATCAAAAAATGGAGAGTTAGTTAGTCCCCAT<br>TTTATGCCCGAAAATTGTGGATGGAACAATGTGTTAACGGAGAGTGTGACATACAC<br>AACTGTATCATCTCACGAGGTAAAACTAGATCCATACCAGATGACTTTCGTGGATTC<br>ACTCTTTCCGGGTGGTAAATGCTCCTCTTCGGTGTGTAGCACCATCTATCATCAAGG<br>AGTCTGGATAAACCCGAGTCACAACTTAGGGTTTTGCAAAGATCCAGTCGATCATC<br>AAGGTCAGTTGTACATGGCTGGTCTTGTTGGAGCTCACGGAGACATTGTGAGAGAA<br>GTGTGGAATCTCAGATCTGTGTTCAAGCCAGAAATTGGTAGATCCAAGCATTTGACT<br>GGATCTTGTTGGATGACATACTGTGATCAAAGAGGACTGAGATTTTCAGATGGAGA<br>ATGGGCAGGTTTCCAAATTCCAGAGATCTCAGCACTGAAACAGGTTCTGCTAGGAC<br>TTCCTGAGTGCAAAGATGATGTGCTGGTTCATGCTCATGACACGAACACTGAGTTGA<br>GGGAAATTTTGGAACACATGGACGAGTCAGCATTGAATGCCATCTGCCAACAAGA<br>AGTCCGCAGGGCAAAAGAGAGAGGAGTGGTTTCTGATTGGCTATTGAGTATGATGA<br>CACCATTCACAGAGGGGTTGGGGCCAGTTTATCGGCTGAACAAAGGAAAACTAGA<br>AGCATCAATGGGATATTATAGAAAGGTATATATAGACTCTAGCAACACCCCTCAGG<br>CCTTCGGACAGACAGAAGATAAAGAATCTGTTGGATGGTCAGATCTTGTACCAAAA<br>GATGCAGATGGAGCTATATCATCAATGTACAACGGGAATGTCGTCATCAATAATCA<br>GATAAAATGGGCAAAAAATGCACTAGGATCTCATATATTAGATGAGATTTCTGCCT<br>TAGAATTTGAAACCCCCGTTGTGCATCATCCTCATTTGACAATCTTGTCGGTAAATC<br>ATAGTGATCTAGTGAGTTCCACACATCCAAATGGGCAAGGAGTTAATCTGATTGAG<br>AGTGTATCTCACTGGGCCGGTGGGTTGTGGGCATCCATAGGATCTGGGTTGATGATC<br>TTGGTTCTAGTGGCTTTAGTCGGATTTTGTACCATCAAGGTATGCTTAGCTTATGTTC<br>CATCGATTTGGGCACAAAAAAATCGGAATGGTAAGAGAAGAGGAACAACATCCCA<br>ACGATCAACAGAACAGGAGATGTTCGAGCTATCAGCCGTGTAGGTACAAGTGATA<br>CTTCAGGTTAAATCGGAACTCTCATTTCTGTGTAATCAGATCTATATATTAAAAAAC<br>CTACTTAAAATATTGCATGTGTTACTGATGTCAGCGTAATAAGCTGAATCATAATTT<br>ACTTCCCACGACAATTTTGTTAACCTACATGTTTTCATCTTATTACATTTTATTTTTAT<br>TTTTATTTTTATGAATATATATGAAAAAAACTATTCAACAGTCATCATGTATGACGA<br>AGATTATTCACGAGGATATGAATCCGATGACCACTATGACCTGCCTGAATGGCTGG<br>AAGAGGATATACAATCAGGAAATCCACTCAATCAAAAAGACTATAGCTTAAATTCT<br>CCTCTCATTGTAGATCTCACAGAAGCGCTGGTAAAATATCTAAATCAAGGTAATAT<br>AGAGAGACGATTCCTCAGACATCAAGACAGATTTGAGAACATACAAAGTGAAATC<br>CGCAACGTTGCATGGAAGAATCCATCGAGCTCAAATCACAGATGGTGGGGGAAGT<br>GGGCTCAACAAACAGAAAAGAGTCCAGAGTTTGTAAGGCTGTTGTCTGATGTTAAC<br>CAGGACATAGAAGAGACTAGTGACGTCTTGATGTCATTCTTGAAAGGATGGATCCA<br>AGACACAACCTCTGTTCCAACAAAATTAAATTGGACATCTACTCAATTGAGCTATG<br>GATCCAAATTCTTCTTCATGCACAAATTGATCCTGTTCATGAATGCGCAATCCGATG<br>AGGAGAGGGTTATTCTCCAAGGGCACATCAAAGTCTCGGAAACAAAGAAGACTGG<br>GATCTACAAAGGTTCTCATCCTAGCTTAGGAGATTTTGTGCTGACCTCTGAATTCTTA<br>CTACTAGAAAGACACAGAGTGATACTAGACCGTTCTTTCCTGTTGATGGTTAAAGAT<br>ACTCTAGTGGGTAGATTTCAGACACTCGCTAGCTTCATGAATAGGGAAGACAAAAA<br>ATATCCAGAGGACGTGATAGAAAAGGTAGAGACCCTTTACTCCTTAGGAGATCAAT	

	TGGTAGAGGATCTAGGGGATGAAGCATACTCGGGTATAAAGTTGCTAGAGCCAGCC<br>TGCAACCTCCGACTCGCAGAGTTGGCTAGAGAATTTAGGCCTTTGATCCCCGAATTC<br>CCACACTTTCGAAACCATGTGGAGACAGCAATAGCAGAGGAGTCTATCCTTAGTCC<br>TGGAATCACAGAATTCTTCAACCATGTAAATAAGGAGACAAATGTTGAAATCATCT<br>TGGCCTACTTCAGCTCTTTCCGACATTGGGGACATCCATACATTGATTATTTCCAAG<br>GGTTAATCAAATTGAACAAGCAAGTCACTCTAGAGAAAGACATCGATACAGAATA<br>CGCCAATGCACTGGCTAGTGATTTGGCCTACATGATTCTTCGAGGACATTTTAACAC<br>TAAGAGAGTATGGGCGGTAGACAAAGCACTTGTGTCAAAGCAACATCCATTGAGTG<br>AGCATATTTTTAATGCCACTTGGCCAACTCCGAAACAGATTGATGACTTCGGAGATC<br>ACTGGCATGAATTGCCTCTGATCAAAATTTATGACATCCCAGATTTAATTGACCCTT<br>CTGTCATCTACTCTGACAAAAGTCATTCAATGGGGAGAGAAGAAGTGTTAAAACAT<br>GTTCAAAGGAATCCAACACAAGCAATTCCTACAAAAAAGGTCTTAGAGACACTCTT<br>ACAAAAACCTGCCACAAATTGGCCAGAATTCTTGTCCTCGATTGAAAAAGATGGCC<br>TACCTAAAGACAGCTTGATCATTGGACTGAAAGGGAAAGAGAGGGAACTGAAAAA<br>AGCTGGAAGGTTCTTTTCTCTAATGTCCTGGGAATTGAGAGAGTATTTTGTAATAAC<br>TGAATACCTGATCAAAACTCATTACGTCCCTCTCTTCAAAGGATTGACTATGGCAGA<br>TGACATGACAGAAGTCGTCAAGAAGATGTTAGAAAGAAGTCAAGGACAGGGAGA<br>GGATGATTACGAGCATGTCAGCATTGCCAACCACATCGATTATGAAAAATGGAACA<br>ACCATCAAAGGAAAGAGTCCAACGGCCCGGTGTTCAGAGTCATGGGACAATTTCTG<br>GGGTACCCTAGTTTGATAGAAAAGACCCATGACTTCTTTGAGCAAAGCTTGATCTAT<br>TATAACGGCAGACCTGACTTAATGCAGACAGACGGAGACGAGTTGCAAAACCGAA<br>CGGAAGCTTTGGTGTGTTGGAATGGTCAGAAAGGAGGATTAGAAGGACTCAGGCA<br>AAAAGGATGGAGCATCCTCAATCTGTTGGTCATCAAAAGAGAATCTAAAATCAGA<br>AACACCAAAGTGCAAACTTTAGCTCAGGGAGACAATCAGGTAGTGTGTACCCAAT<br>ACCGCATTATGCCAACCAGATCGACACTTGAATTGCAGGCAGAACTTGAAAAAGTT<br>AAGAAGAACAATCAAGTTATTATGGATGCTATTGAGACGGGGACTAACAAATTAG<br>GGCTACTGATAAACAATGATGAAACCATTCAATCCGCTGATTTCCTCACATATGGTA<br>AGGTCCCAATATTTAGAGGTAATATTCGCTGCTTGGAAACTAAAAGATGGTCTAGA<br>GTGACTTGTGTCACTAATGACCAATTGCCATCTCTTTCCAATGTCATGTCATCTGTGT<br>CGACAAACAGTCTGACGGTCTCGCATTTTGATGTCAGTCCTATAGAATCCATGAGAC<br>AATACCTGTTCTTCGGAAATTTTGCTCGGAGATTGGTGGAATTCCATAATCCTGCAA<br>TGAGAGTGCCGATTAGTTTAGAAGACCTGGATTCCAAACAAAAGTCTGTTTATCTGA<br>ACGCAGTGCTTTTCTTGGATCCGTCTCTGGGTGGTGTGTCTGGTATGTCATTGTCAAG<br>ATTCTTGACTAGGATGTTTCCTGATCCCATCACAGAAGGATTATCTTTTTGGAAAAT<br>CGTTTACGAACATACCGCTTCTAAAGACACTCAACTGCTCTGCAGAATCGCTGGAA<br>GCCCCGAATTGGCTAGACGCCAGAACAATCTGGACAAGTTGATAGAGAATCCAAC<br>AGCATTGAATCTGAGCAAGGAAACTTCTGCACTGAGTGTCATTAAGAAAGAAGTCA<br>GGTCACGCCTGTACAAGGATTGCGACAAATTCAAGAACAAATTGATTGCAGATGCA<br>ATTGGGATTGCTAGGGATGAAGAGGCTCACCTGGAATTATTCTTGATGTCAATCCGA<br>CCATTATTTCCAAGATTCCTAGCAGAGTTCAAAGCAGCCACGTTCGTTGGCATCACA<br>GAATCTCTCATTTCTTTATTCCAAAATTCCAAAACCATTCGCAACATCTTCAGGAAG<br>AAATATGCAAAAGAATTGGAACTTAGAGTAGTGCAGTGTGAATACAGATCCATCA<br>ATTTGATGTTGAGCCTCGCAGATCGATCCCATTTAGATGAAATGTGGACATGCTCGG<br>CATCTAAAGCAGATGAACTCCGAACCTTATCATGGGGGACTACAATAATTGGAACA<br>ACTGTCCCTCATCCTTTAGAGATGATCAATCATGCTCATATAGGACAGAAATGCGA<br>CTCGTTAGAAACCCTGGACTACATAAATGTCACTGTGGTTCAAGATCTGACTGATTG<br>TCTAACTAGCAAAGGGAAGTTACCAGCGTATTTAGGATCCAAAACATCGGAGACTA<br>CAAGCATCTTGCAACCATGGGAAAAGGAGACAAAGATTCCTGTGATTCGTAGAGCT<br>GCTAAATTGAGAGCTGCAATCACCTGGTTTGTAGAGCCTGATTCTCTTTTGGCACAA<br>AGCATCCTGAACAACATTGAAAGTCTGACTGGAGAAGATTGGTCTGCTTCAATATC<br>TGGATTCAAAAGGACAGGATCTGCACTACATCGCTTCACTAGTGCTAGAGTGAGTG<br>CAGGAGGCTTTTCAGCTCAAAGCCCGGCCAGATTGACCAGGATGATGGCAACAAC<br>AGATACTTTCCGAGAAATTGGGTCAGATAATTACGATTTTATGTTCCAATCATTACT<br>ACTCTTTGCCCAAATGACTACAGGTGAGATTTACAAAAGGAGTCCAGCTACTAATTT<br>CCACTTTCATCTGAGTTGCCACCAATGTCTTCGTAAGATCGAAGAGCCTACCTTAAA<br>CTCTGATTTTGCCTACAACCCTATTCAGAGATCGGACATCTTGGATAAGTGGAAACC<br>TCAGACCACAGATTGGTCTTCAGAAAGAAAGGCTCCGGAGATAGAGGAAGGGAAT<br>TGGGACAGATTGACACATCAGGAGCAAAGTTTTCAGATAGGGAAGTCCATCGGGTT<br>TCTGTTTGGAGATTTGACAATGACAAAGAATTCACATGCTCAGGATTCTTCTATTTTC<br>CCTTTATCAATTCAATACAAGATCACAGCTGCGGAGTTTCTGGAAGGAATTCTAGAT<br>GGGATAGTCAAGGCTTCTGCTTTGTCTACTATCCACCGGAGGAACTTCGATCATCAC<br>AGCAAGTACAAGTCCACTGTTTCAGGAACTGTTGATTATTTAATCGAATTGATTTCA<br>GAGTCCGCGGGGTTCACAAATCTGACCAGGAATGGTCCCTTGAAGGCTTGCTTACT<br>GACCATTCCACATAAGATACCTCCATCCTATCCTCTCAGCCAATCTGATCTTGGTGC<br>TATGTCAAGAAACTACCTCAGACTGTTGCACCGAAGAATGTCTACCGGCACCTACA<br>AGACCAGGTGGCCGACAAATTGGATATTCTCTGATATGATGAGCCCTAACATAATC<br>TATCCATTTGTGATCAGTGTCTCCTGCGTTGGACTGGCTTACTCATCATCTTGGACAA<br>AGAAATCTGCTGACAAATTGAGAGGGTTAAGAGGAGTAGCTGAGTTGATTCGATCA<br>TCAGATGATGTGCAATTGCCTGTTGGGAAGCTATTCAAAACAGTAAACCAAGAAAT<br>TAGACATGCCATAAAACATCATGCATCGGACGATGCCGAAATCCCCGAAAGCCAC<br>ATCCAAGAAGGCTGGAAAAAAGAGCTCATAGTCAACATTAACATGCAGCCAATTG<br>ACTATAGTAGAACAGCGACAGCGAAGTCGTTAGACCGGCCTGCTCAAATTCGAGA<br>CCCTTTAATATCTGGACTAAGAACAGCACAATTGGCCACTGGATCCCACTACAAGT<br>TACGATCCATACTAGTGCAAAACCGAATCCAAGTTACCGATGCTCTGTGCGGAGGG<br>GATGGATCTGGTGGAATCGGGGCTTGTGTCCTGAGACAATACCCGTTTGCTAAGCTG<br>ATTTACAACAGTCTATTCGAGATTCAAGATCTGGATATGAGGGGGAGTGCACCTGG<br>ACCGCCATCTGCTATTGCCGCAATGGGAAACATGAGTATGAGGTGTGTCAATCGGG<br>ATTCTGCATGGAAAAATCCATCTGATCTGAGCCACACTTCCACCTGGGAATATTTTC<br>AATCGTTGATGACCCAACATCAGTTGCGGTGCAATCTGTGGACCTTTGACATGGAG<br>GTTCGCAGCCATGACATTTCAGATGCTATAGAAAAGCAAATAGTGGCCAATCTTCA	

	TTTGCTACCAAAAAATGGGACAATCATATACAAGACATATCTAACGAAATTGTCTG<br>ACATGGAGACAACAGTCTTGGACAGACTGGGTGGATTCTTCAAAAGAGTCAGTCTT<br>GTGTCTACAGACGCTACATCAAGCCATAGCTCTGAAGTCTACGCTGTATTCCAAAA<br>CAAACTGGATAAGAGACAATTGGAAATCCACCCCAACTGGTCTTCCTGCAATTTAG<br>GTTCTGACATACACCCATGCTGGAAAAGTGAAGATGAGGAGTTTGAAAGGGCAAG<br>AAGATTCTTTCACATGAAGAGACAACAAGGGGTGCCGACTCGATTGAGGCCTACTT<br>TGGATTCCGAAATCCAGGTCCTGAGCGTCTCGGCCGGAGTTGAAAACGGGGTAGCA<br>ATGACATTAGCTATGGACGTAAGCAATCAGGTCTCGGATCCAACCACTGGGGCATT<br>TCTTTGGCTTTTAGTAACTTTGCAACATATATGCCCAATAGGCCCATCATTCAAGAT<br>CCCATCCAGTTCTGCAGTTGAATCTTATTTGGCCATACTGATTGGATTCAGTAGTATC<br>TACCAGCTTCAGACAGGCAATAACAAAGCTTATGCCAGCATCAAGACCTGCTTGTC<br>TCAATCTGCTCCATTCTTCTGCAACCCTGATGGATGGAGTTGCATTAAAGGACTTGA<br>CAAGTCTATGCGGATGGACAGAAAGTTGGCTTTAGTGGGCAGTGTGATACGGGCAT<br>GGTCAAAGTGGAACTTGACTCAGTCAATCAACTTTCACAAACTAGATGGTATGATG<br>AGACATTATCTACCGAAGGGAACATTGAGGCAGATTGGCTGCAAAACCGGGATCT<br>GGGACTATATCAATGGGGCAGTGAAAGGAGTACGAAGTGCTGCCAATCAGGACAG<br>TCCAAAAGAGTCAGCAGCTGCTTGGAGAGATTAGTATATATAACATTGTGTCTATC<br>ATCCAATTCATTATTAGATAGGTGACAATTAGTTAAAGAACAAGATTACCCCATGT<br>AAGAGAATTTTTTATGTCCTAATTTGCCGTATGCACTTGTAAATAAATGTGTATTGTA<br>ATGTATATGTGTATGTGTATTTGATGTATTGGTGTATATGTCAACAAATCAATAATAT<br>GATCACATAGGATAAGGTCAAGACCAATTGTTTAAGTTTTATTAGAATTCCGTGAAT<br>ATGAAAAAAACAAAACAGGTCCTCTCGGATAAATCCTTAATAATGGGG	
KC608035	TAAGGCTATTCTTTAACAGACATCTGTTTGTTTCATTGAAAATGGCTGCCATGAACT<br>CCATCAAAGTTACAGTTAGAGCCAATGGACAAGATTTCACCCCAGTTTGTCCTAAA<br>ATGGAGGATGAAGTGGCTTACCCATCTGATTATTTAGATGCTAATGGATTACCAACC<br>TTCCAGCTCTACTATCATGATCTGTCAAAAAAGGATCTGCTAGACCTGATCTGGGGA<br>GAAGCATCAGAGGCAAGGCTGCCTAGTGAGCTGGTGACCGCGTACGTGTACAAGG<br>TTGTGAGTGAGTGGAAAGAGAAGCTGGAGAGTGATTGGTCATCTTTCCGATTCCAG<br>ATTGGCAAAGCGGGAGAGGAGATAACCCCATTCAACCTGATTGGCATGACTGTGA<br>ACGGTCAGAAACTAGCTGACTACAAAAAAGCAGTTGCTCCTGAGGGGATAGATGA<br>GGTAGCAATGGTGATCTATCTCCTGGCACCATACCGGATAGTCGGAATTAAAAACG<br>AAGACTATCAAGATCGGGTGATCACCAACATCCAGAACCAGCTGGACAGTCTCGG<br>CGCAAAGAAGCTGCAGGTGAAGGCACTGAAGAATGTCACTACTCTCATCAACAGT<br>GCCAACTATCTGAAAATGGTGGCAGTGATTGACATGTTCTACTACCATTTCAAGAAC<br>AGTCAAGAAAGAGCTGTTGTCAGGATTGCCACTCTGAGTTCCCGTCACAAAGACTG<br>TGCAGCTCTATCCACCCTGAATCACATCACCAGCTTCACTGGGAGGAGTTTTGTGCA<br>GGTGTTGGATTGGGTTTTCACAGATCAAGTTGCCAAGGAGATCGGCAGGATGATGC<br>GTGCGGGTCAAGAAATTGATCGTCCTGAATCCTACATGCCGTACCTGAAGGACTTG<br>GGGCTGAGCAGAAAATCCCCCTACTCCTCCTCAGCAAATCCTGGCACTCACTGCTG<br>GGCACAGATGGTATGTGCCATGATGGGATCCAAAAGATCTCAAAACGCAATCGCC<br>AGCACTGAAGAGAATCTCAGCAATCTGACAAGAAATGCTGAGATTATGGCCTATGC<br>CCTGGGAGTGGGAGCAGACCTGGTGAAGGGACTGATCATTGGAGATCAGAAGGAA<br>GGAGACTCCGGTGTCATCCAGGATGAGGAGGGAATGGATGAGCCCAACAACATGG<br>AGGCCCAAGATTGGCTGGAGTACATGGCCTCAAAGGGATTCAAGCTGACTCCAAAT<br>ATGGAGCTGCAAGTCCGTCACATGTGCCTGCGAATCACCAATCCCAGAAAAGCCAC<br>TCTGGGGAGTTACCTGCGGGAGAGATACAGTTCATCCCTGTGAGTTGAAGCAGAAA<br>TGAGTCATTTTTAGAACTATTATATTTTTATCCTAATAGCCTTAAATAGTTAGTCATC<br>TTGTGCTTAGATATTATGCCCTAGTTATATGAAAAAAACTTTAACAGGGATAAACAT<br>AGTTTCTACGGCTCTATTTTATCTCTCATATCTTAATTAACATGTTGTCATCTAACAA<br>AAACAAAAACAAATTCGACTTAGAGGGGATACAATTGCTGGCAAAAGGAGTCAAA<br>AATGCAGGAGAATCCATAGATAAAGCCACAGATGAGAAGCCTGAACATCTCTCTG<br>ACGCATTTTCAAAATACACAGAATATTTGTCAAATGAGAGCAAGGGGGAGGAGGA<br>GGATTTCCAATTCGAACAGGTGGAATATGGATTTCAAGAGTCACCTGAATCCAATA<br>ACCCTCTGCAAGAGAGCTGGATAGCGAAAGATGACCTGGAACCAGATATGGGAAA<br>GTCCTGGGTAGAGTATCAAGCTAAGATGTCCTTTGATTACAGTGAGCAGGTGAAAC<br>CCACTGTGATGAGGGAGATCAACGGCTTGCTGGGTATGCTGGGTGGATTTGCTAAA<br>TTCCAAGATGGAAAGAAAGAATATCTTTTCTATCTTCCAGAGAAGAAAGAGTCAGA<br>AGAGAGAAAGTCTGATAAGAAACAATGTCCTTTCAAGGTTGATGTCACCCCAAAAC<br>AAGACCCAAAAATCACTCCCACACCTGAGAAAGAGCCAAAACATCTGCCCACATC<br>AGCTGCCGGCAAAGAAAGCACCGAAGAAGCAGCCGTGATGCAAGGATTCTGGATG<br>GACGGAATGAGGCTGACAGAGAAGACATCAGGGAAGTATTGCCTGTTCTTCCCACA<br>AAAAATGGGATGGTCTCAAGCCGAATGGATCAACAAATCAGAAGACATTAATCCC<br>CGAACTCTGGCACATGACATCTTCAAATGGATGGTATCCAAATCTCCGAAACGGGC<br>CACATACTTGAGGAAATACATGGTAGAAGAGTAACCACAGGTCTGAACCTCTTGAC<br>TATTTTTGATGCCTTAAATTGGCTTGACGTACATGTATTACTTATTACATATCTCAGTT<br>ATTTATATGAAAAAAATGTTAACAGACGTCAATAACATTTGTTATATGCGCTATCAT<br>CGTGAGACTACATTCAAACGTCGAATCAATTGAAGTTAAATGGCTCTATCTATATTT<br>AAGAAGAAGGGGAAGAAAACAGAATCTCCCAAGATGTTTCTAATGGATGCAAACC<br>AGATGACCCCGAGTGCTCCACCCCCTTACCAGGCAGAACCTGGTCCGTTCGACACT<br>TGGGGGAATGAGGAACTAGAAGAAGCTATGAAAGTGTGTTACTTAGTAGACACCT<br>GCTTGTCTGTGACAACAAGAGAACCCATTCGATCAGTGGTGGATGCCTATATTATA<br>GCACAAGGAGTCTTAGATCATTACACCGGCCCGATCCTGACCCGTCCGTTTTACATT<br>GCCTTGTTCTTGGGCGGGATTCACGGGATGCAGGCGGGGGTTAAAGGAGCGAGAA<br>GCATCCGGTATGAAAGAGAACATCATGGACCTCTGGTGTTCCCATATCACAGAAGC<br>AACCCGCTCGATTGGACTCCCCGGGCCATTGAGTTCCAGTACACCACTAGTCTGCG<br>GGGGAAGCCGGTGGAGGTGAGCTTCCAGGCTCGTCTGCAAGCTACCAGGCAATTTG<br>GACCTGGTGTAGAGGTTTATCTGGACGGCCTGCAAAGGATAGAACGCCCTAGCAAC<br>GAACTAGTTCTCAAGCAATTCAGGGTCCCCCTGCTGATGCTGGAGAAAGGGAAATG<br>GGTGTTCAATCTTGATTCTGAGGGTAGTTACAATTAAAATGGTCCAACAATCACATC<br>ATCTAAGCCTCTTCTGCTATAATCATACAGTCAAGTTTACCGCCACAAATGGGTTAA	KC608035.1 Eel virus European X isolate DK3631, partial genome [54]

	ATAGCATATAGACACATAGAAATGCATCATATTATTAATTAATTAATTGTTAAGTTC<br>TATGAAAAAAACTAACAGTGATACACCATTATTTTGAGATATTTGTCACTGTGAAAT<br>TCAGTTGGTTCAATAGAGACATGGATACTTTGATTAAAATTTTGCTGATTATAGTCA<br>TTCTAAAGTCTCTCCATGCTCACATCGAATTCGTACCACATGATCTGAGTAAATGGA<br>GAGACATCAGCATAGGGCACTTGGATTGTCCAATCTATGGAGATTTGTCTAATCAA<br>GCAACGAGAACAATCCCTGTGAAATACAGCAGTGTTCAATGGGGCCTAAAAAATA<br>ATATTGATGGGTACCTCTGTATATCTGCCAAATGGTCAGTCACTTGTGATTATAGGT<br>GGTATGGCTCCAAGTACATTTCAACCTCAATTGAATATGTTCCAACAAAAGAATCA<br>GAATGCAGAGATGCCATCAAGTCATCAAAAAATGGAGAGTTAGTTAGTCCCCATTT<br>TATGCCGGAAAATTGTGGATGGAACAATGTGTTAACGGAGAGTGTGACATACACAA<br>CTGTATCATCTCACGAGGTAAAATTAGATCCATACCAGATGACTTTCGTGGATTCAC<br>TCTTTCCGGGCGGTAAATGCTCCTCTTCGGTGTGTAGCACCATCTATCATCAAGGAG<br>TCTGGATAAACCCGAGTCACAACTTAGGGTTTTGCAAAGATCCAGTCGATCATCAA<br>GGTCAGTTGTACATGGCTGGTCTTGTTGGAGCTCACGGAGACATTGTGAAGGAAGT<br>GTGGAATCTCAGATCTGTGTTCAAGCCAGAGATTGGCAGATCTAAGCATTTGACTG<br>GATCTTTTTGGATGACATACTGTGGTCAAAAAGGACTGAGATTTTCAGATGGGGAAT<br>GGGCAGGTTTCCAAATTCCAGAGATCTCAGCACTGAAACAGGTTCTGCTAGGACTT<br>CCTGAGTGCAAAGATAATGTGCTAGTTCATGCTCATGACACGAACACTGAGTTGAG<br>GGAAATTTTAGAACACATGGATGAGTCAGCATTAAATGCCATCTGCCAGCAAGAGG<br>TCCGCAGGGCAAAAGAGAGAGGAGTGGTTTCTGATTGGCTATTAAGTATGATGACA<br>CCATTCACAGAGGGGTTGGGGCCAGTTTATCGGCTGAACAAAGGAAAACTAGAAG<br>CATCGATGGGATATTATAGAAAGGTATACATAGACTCTAGCAACACCCCTCAGGCC<br>TTCGGACAGACAGAAGATAAGGAATCTGTTGGATGGTCAGATCTTGTACCAAAAGA<br>TGCAGATGGAGCTATATCATCAATGTACAACGGGAATGTCGTCATCAATAATCAGA<br>TAAAATGGGCAAAAAATGCACTAGGATCTCATATATTAGATGAGATTTCTGCCTTA<br>GAATTTGAAACCCCCGTTGTGCATCATCCTCATTTGACAATCTTGTCGGTAAATCAT<br>AGTGATCTAGTGAGTTCCACACATCCAAATGGGCAAGGGGTTAATTTGATTGAGAG<br>TGTATCTCACTGGGCCGGTGGGTTGTGGGCATCCATAGGATCTGGGTTGATGATCTT<br>AGTTCTAGTGGCTTTAATTGGATTTTGTACCATCAAGGTATGCTTAGCTTATATTCCA<br>TCAATTTGGGCACAGAAAAATCGGAAGGGGAAGAGAAGAGGAACAACATCCCAA<br>CGATCAACAGAACAGGAGATGTTCGAGCTATCTGCTGTGTAGGTACAAGTGATACT<br>TCGAGTTCAATCGGAACTCTCATTTCTGTGTAATCAGATCTATGCATTAAAAAACCT<br>ACTTAAAATATTGTATGTGTTACTGATGTCAGCGTAGTAAGCTGAATCATAATTTAC<br>TTCCCACAATAATTTTGTTAACCTACATGTTTTCATCTTATTATATTTTATTTTTATTTT<br>TATTTTTATGAATATATATGAAAAAAACTATTTAACAGTCATCATGTATGACGAAGA<br>TTATTCACGAGGATATGAATCCGATGACCACTATGACCTGCCTGAATGGCTGGAAG<br>AGGATATACAATCAGGAAATCCACTCAATCAAAAAGACTATAGCTTAAATTCTCCT<br>CTCATTGTAGATCTCACAGAAGCGTTGGTAAAATATCTAAATCAAGGTAGTATAGA<br>GAGACGATTCCTCAGACATCAAGACAGATTTGAGAACATACAAAGCGAAATCCGC<br>AACATTGCATGGAAGAATCCATCTAGCTCAAATCACAGATGGTGGGGGAGATGGG<br>CTCAACAAACAGAAAAGAGTCCAGAGTTTGTAAGTCTGTTGTCTGATGTTAACCAG<br>GACATAGAAGAGACTAGTGACGTCTTGATGTCATTCTTGAAAGGATGGATCCAAGA<br>CACAACCTCTGTTCCAACAAAATTAAATTGGACATCTACTCAACTGAGCTATGGAT<br>CCAACTTCTTCTTCATGCACAAATTGATCCTGTTCATGAATGCGCAATCCGATGAGG<br>AGAGGGTTATTCTCCAAGGGCACATCAAAGTCTCGGAAACAAAAAAGACTGGGAT<br>CTACAAAGGTTCTCATCCTAGCTTAGGAAATTTTGTGCTGACCTCTGAATTCTTACTA<br>CTAGAAAGACACAGAGTGATACTAGACCGTTCTTTCCTGTTGATGGTTAAAGATACT<br>CTAGTAGGGAGATTTCAAACACTCGCTAGCTTCATGAATAGGGAAGACAAAAAAT<br>ATCCAGAAGACGTGATAGAAAAGGTGGAGACCCTTTATTCCTTAGGAGATCAATTA<br>GTAGAGGATCTAGGGGATGAAGCATATTCGGGGATAAAGTTGCTAGAACCAGCCT<br>GCAACCTCCGACTCGCAGAGTTGGCTAGAGAATTTAGGCCTTTGATCCCCGAATTCC<br>CACACTTTCGAAACCATGTGGAGACAGCAATAGCAGAGGAGTCTGTCTTTAGTCCT<br>GGAATCACAGAATTTTTCAGCCATGTAAATAAAGAAACAAATGTTGAAATCATCTT<br>GGCCTACTTCAGCTCTTTCCGACATTGGGGACATCCATACATTGATTATTTCCAAGG<br>GTTAATCAAATTGAACAAACAAGTCACTCTAGAAAAAGACATTGATACAGAATAC<br>GCTAATGCACTGGCTAGTGATTTGGCCTACATGATTCTTCGAGGACATTTTAACACT<br>AAAAGAGTCTGGGCGGTAGACAAATCACTTGTGTCAAAGCAACACCCGTTGAGTG<br>AGCATATCTTCAATGCCACTTGGCCAACTCCAAAACAGATTGATGACTTCGGAGA<br>TCACTGGCATGAATTGCCTCTGATCAAAATTTATGACATCCCAGATTTAATTGACCC<br>ATCTGTCATCTACTCTGACAAAAGTCATTCGATGGGGAGAGAAGAAGTGTTAAAAC<br>ATGTTCAAAGGAATCCAACACAAGCAATTCCTACAAAAAAGGTCTTAGAGACACT<br>CTTACAAAAACCTGCCACAAATTGGCCAGAATTCTTGTCCTCGATTGAAAAAGATG<br>GCCTACCTAAAGACAGCTTGATTATCGGACTAAAGGGAAAAGAGAGGGAACTAAA<br>AAAAGCTGGAAGGTTCTTTTCTCTAATGTCCTGGGAATTGAGGGAGTATTTTGTAAT<br>AACTGAATACCTGATCAAAACTCATTACGTCCCTCTCTTTAAAGGATTGACCATGGC<br>AGATGACATGACAGAAGTCGTCAAGAAGATGTTAGAAAGAAGTCAAGGACAGGG<br>AGAGGATGATTACGAGCATGTCAGCATTGCCAACCACATCGATTATGAAAAATGGA<br>ACAACCATCAAAGGAAAGAGTCCAACGGCCCGGTGTTCAGAGTCATGGGACAATT<br>TCCGGGGCACCCTAGTTTGATAGAAAAGACCCATGACTTCTTTGAGCAAAGCTTGA<br>TCTACTATAACGGCAGACCTGACTTAATGCAGACAGACGGAGACGAGTTGCAAAA<br>CCGAACAGAAGCTTTGGTGTGTTGGAATGGTCAGAAAGGAGGATTGGAAGGACTC<br>AGGCAAAAAGGATGGAGCATCCTCAATCTATTGGTCATCAAAAGAGAATCTAAAA<br>TCAGAAACACCAAAGTGCAAACTTTAGCTCAGGGAGACAATCAGGTAGTGTGTAC<br>CCAATACCGCATTATGCCAACCAGATCGACACTTGAATTGCAGGCAGAACTTGAAA<br>AAGTTAAGAAGAACAATCAAGTTATTATGGATGCTATTGAGACGGGGACTAACAA<br>ATTAGGGCTACTGATAAACAATGATGAAACCATTCAATCCGCTGATTTCCTCACATA<br>TGGTAAGGTCCCAATATTTAGAGGTAATATTCGCTGCTTGGAAACTAAAAGATGGT<br>CTAGAGTGACTTGTGTCACTAATGACCAATTGCCATCTCTTTCCAATGTCATGTCATC<br>TGTGTCGACAAACAGTCTGACGGTCTCGCATTTTGATGTCAGTCCTATAGAATCCAT<br>GAGACAATACCTGTTCTTCGGAAATTTTGCTCGGAGATTGGTGGAATTCCACAATCC	

	TGCGATGAGAGTGCCGATTAGTCTAGAAGACCTGGATTCCAAACAAAAGTCTGTTT<br>ATCTGAACGCAGTGCTTTTCTTGGACCCATCTCTGGGTGGCGTGTCTGGTATGTCATT<br>GTCAAGATTCTTAACTAGGATGTTTCCTGATCCCATCACAGAAGGATTATCTTTTTG<br>GAAAATCGTTTACGAACATACCACTTCCAAAGACACTCAACTGCTCTGCAGAATCG<br>CTGGAAGCCCCGAATTGGCTAGACGCCAGAACAATCTGGACAAGTTGATAGAGAA<br>TCCAACAGCATTGAATCTGAGCAAAGAAACTTCTGCCCTAAGTGTCATTAAGAAAG<br>AAGTCAGGTCACGCCTGTACAAGGATTGCGACAAATTCAAGAACAAATTGATTGCA<br>GATGCAATTGGGATTGCTAGAGATGAAGAGGCTCACCTGGAATTATTCTTAATGTC<br>AATCCGACCATTATTTCCGAGATTCCTGGCAGAGTTCAAAGCAGCAACATTTGTTGG<br>CATCACAGAATCTCTCATTTCTTTATTCCAAAATTCCAAAACCATTCGCAACATCTTC<br>AGGAAGAAATATGCGAAAGAATTGGAACTTAGAGTAGTGCAGTGTGAATACAGAT<br>CCATCAATTTGATGTTGAGCCTCGCAGATCGATCCCACTTAGATGAAATGTGGACAT<br>GCTCGGCATCTAAAGCAGATGAACTCCGAACCTTATCATGGGGGACTACAATAATT<br>GGAACAACTGTCCCTCATCCTTTGGAGATGATCAATCATGCTCATATAGGACAGAA<br>ATGTGACTCGTTAGAAACCCTGGACTACATAAATGTCACTGTGGTTCAAGATCTGAC<br>CGATTGTCTAACGAGCAAAGGGAAGTTGCCAGCGTATTTAGGATCTAAAACATCGG<br>AGACTACAAGCATCTTGCAACCATGGGAAAAAGAGACAAAGATTCCTGTGATTCG<br>CAGAGCTGCTAAATTGAGAGCTGCAATCACCTGGTTTGTAGAGCCTGATTCTCTTTT<br>GGCACAAAGCATCCTGAACAACATTGAAAGCCTGACTGGAGAAGACTGGTCTGCTT<br>CAATATCTGGATTCAAAAGGACAGGATCTGCACTACATCGCTTCACTAGTGCTAGA<br>GTGAGTGCAGGAGGCTTCTCAGCTCAAAGCCCGGCCAGATTGACCAGGATGATGGC<br>AACAACAGATACTTTCCGAGAAATTGGGTCAGATAATTACGATTTTATGTTCCAATC<br>ATTACTACTCTTCGCCCAAATGACTACAGGTGAGATTTACAAAAGGAGTCCAGCTA<br>CTAATTTCCACTTTCATCTGAGTTGCCACCAATGTCTTCGTAAGATCGAAGAGCCTA<br>CCTTAAACTCTGATTTTGCCTACAACCCTATTCAGAGATCGGACATCTTGGATAAGT<br>GGAAACCTCAGACCACAGATTGGTCTTCGGAAAGAAAGGCTCCAGAGATAGAGGA<br>AGGGAACTGGGACAGATTGACACATCAGGAACAAAGTTTTCAGATAGGGAAGTCT<br>ATCGGGTTTCTGTTTGGAGATTTGACAATGACAAAGAATTCACATGCTCAGGATTCT<br>TCTATTTTCCCTTTATCAATTCAATACAAGATCACAGCTGCGGAGTTTCTGGAAGGA<br>ATTCTAGATGGGATAGTCAAGGCTTCTGCTTTGTCTACTATCCACCGGAGGAACTTC<br>GATCATCACAGTAAGTACAAGTCCACTGTTTCAGGAACTGTTGATTATTTAATCGAA<br>TTGATTTCAGAGTCCGCGGGGTTCACAAATTTGACCAGGAATGGTCCCTTGAAGGCT<br>TGCTTACTGACCATTCCACACAAGATACCTCCATCCTATCCTCTCAGCCAATCGGAT<br>CTTGGTGCTATGTCAAGAAACTACCTCAGACTGTTGCACCGGAGAATGTCTACCGG<br>CACTTACAAGACCAGGTGGCCGACAAATTGGATATTCTCTGATATGATGAGCCCTA<br>ACATAATCTATCCATTTGTGATCAGTGTCTCTTGTGTTGGACTGGCTTACTCATCATC<br>TTGGACAAAGAAATCTGCTGATAAATTGAGAGGGTTAAGAGGAGTAGCTGAGTTGA<br>TTCGATCATCAGATGATGTGCAATTGCCTGTTGGGAAGTTATTCAAAACAGTAAACC<br>AGGAAATTAGACATGCCATAAAGCATCATGCATCAGACGATGCTGAAATCCCCGA<br>AAGCCACATCCAAGAGGGCTGGAAAAAAGAGCTCATAGTCAACATCAACATGCAG<br>CCAATTGACTATAGTAGAACAGCGACAGCGAAGTCGTTAGACCGGCCTGCTCAAAT<br>TCGGGATCCTTTGATATCTGGATTAAGAACAGCACAATTGGCCACTGGATCCCACTA<br>TAAGTTACGATCTATACTAGTGCAAAACCGAATTCAAGTCACCGATGCTTTGTGTGG<br>AGGGGATGGATCTGGTGGAATTGGAGCTTGTGTCCTGAGACAATACCCGTTTGCTA<br>AGCTGATTTACAACAGTCTATTCGAGATTCAAGATCTGGATATGAGGGGGAGTGCA<br>CCTGGACCACCATCTGCTATTGCCGCAATGGGGAACATGAGCATGAGGTGTGTCAA<br>TCGAGATTCTGCATGGAAAAACCCATCTGATCTGAGCCACACTTCCACCTGGGAAT<br>ATTTTCAATCGTTGATGACCCAACATCAATTGCGGTGCAATCTATGGACCTTTGACA<br>TGGAGGTTCGCAGCCATGACATTTCAGACGCTATAGAAGAGCAAATAGTAGCTAAT<br>CTTCATTTGCTACCAAAAAATGGGACAATCATATACAAGACATATCTAACGAAATT<br>GTCTGACATGGAGACAACAATCTTGGACAGATTGGGTGGATTCTTCAAGAGAGTCA<br>GTCTTGTGTCTACAGACGCTACATCAAGCCATAGCTCTGAAGTCTACGCTGTATTCC<br>AAAACAAACTGGATAAGAGACAATTAGAAATCCACCCCAACTGGTCTTCTTGCAAT<br>TTAGGTTCTGACATACACCCATGCTGGAAAAGTGAAGATGAGGAGTTTGAAAGGGC<br>AAGAAGATTCTTTCACATGAAGAGACAACAAGGGGTGCCAACTCGATTGAGACCT<br>ACTTTGGATTCCGAAATCCAGGTCCTGAGCGTCTCTGCTGGAGTCGAAAATGGGGT<br>AGCAATGACATTAGCCATGGACGTAAGCAATCAGGTCTCAGATCCCACCACTGGAG<br>CATTTCTTTGGCTTTTAGTAACCTTGCAACATATATGCCCAATAGGCCCATCATTCAA<br>GATCCCATCCAGTGTTGCAGTTGAATCTTATTTGGCCATACTGATTGGATTCAGTAGT<br>ATCTACCAGCTTCAGACGGGCAGTAACAAAGCTTATGCCAGCATCAAGACCTGCTT<br>GTCTCAATCTGCCCCATTCTTCTGCAACCCTGATGGATGGAGTTGCATTAAAGGACT<br>TGACAAGTCTATGCGGATGGACAGAAAGTTGGCTTTAGTAGGCAGTGTGATACGGG<br>CATGGTCGAAGTGGAACTTGACTCAGTCAATCAACTTTCGCAAACTAGATGGTATG<br>ATGAGACATTATCTACCGAAGGGAACATTGAGGCAGATTGGCTGCAAAACCGGGA<br>TCTGGGACTATATCAATGGGGCAGTGAAAGGAGTACGAAGTGCTGCCAATCAAGA<br>CAGTCCAAAAGAGTCAGCAGCTGCTTGGAGAGATTAGTATATATAACATTGTGTCT<br>ATCATCCAATTCATTATTCGATAGGTGACAATTAGTTAGAAAACAGGATTACCCCAT<br>GTAAGAGAATTTTTTATGTCCTAATTTGCCGTATGCACTTGTAAATAAATGTGTATTT<br>TAACGTATGTGTGTATGTGTATTTGATGTATTGGTGTATATGTCAACAAATCGATAAT<br>ATGATCACATAGGATAAGGTCAAGACCAATTATTTAAGTTTTATTATAATTCCGTGA<br>ATATGAAAAAAACAAAACAGGTCCTCTCGGATAAATCCTTAATAATGGGG	
KC608036	TAAGGCTATTCTTTAACAGACATCTGCTTGTTTCAGTGAAAATGGCTGCCATGAACT<br>CCATCAAAGTTACAGTTAGAGCCAATGGACAAGATTTCACCCCAGTTTGTCCTAAA<br>ATGGAGGATGAAGTGGCTTACCCATCTGATTATTTAGATGCTAATGGATTACCAACC<br>TTCCAGCTCTACTATCATGATCTGTCAAAAAAGGATCTGCTAGACCTGATCTGGGGA<br>GAAGCATCAGAGGCAAGGCTGCCTAGTGAGCTGGTGACCGCGTACGTGTACAAGG<br>TTGTGAGTGAGTGGAAAGAGAAGCTGGAGAGTGATTGGTCATCTTTCCGATTCCAG<br>ATTGGCAAAGCGGGAGAGGAGATAACCCCATTCAACCTGATTGGCACGACTGTGA<br>ACGGTCAGAAACTAGCTGACTACAAAAAAGCAGTTGCTCCTGAGGGGATAGATGA<br>GGTGGCAATGGTGATCTATCTCCTGGCACCATACCGGATAGTCGGAATTAAAAACG	KC608036.1 Eel virus European X isolate DK5743, partial genome

	AAGACTATCAAGATCGGGTGATCACCAACATCCAGAACCAGCTGGACAGTCTCGG<br>CGCAAAGAAGCTGCAGGTGAAGGCACTGAAGAATGTCACTACTCTCATCAACAGT<br>GCCAACTATCTGAAAATGGTGGCAGTGATTGACATGTTCTACTACCATTTCAAGAAC<br>AGTCAAGAAAGAGCTGTTGTCAGGATTGCCACTCTGAGTTCCCGTCACAAAGACTG<br>TGCAGCTCTATCCACCCTGAATCACATCACCAGCTTCACTGGGAGGAGTTTTGTGCA<br>GGTGTTGGATTGGGTTTTCACAGATCAAGTTGCCAAGGAGATCGGCAGGATGATGC<br>GTGCGGGTCAAGAAATTGATCGTCCTGAATCCTACATGCCGTACCTGAAGGACTTG<br>GGGCTGAGCAGAAAATCCCCCTACTCCTCCTCAGCAAATCCTGGCACTCACTGCTG<br>GGCACAGATGGTATGTGCCATGATGGGATCCAAAAGATCTCAAAACGCAATCGCC<br>AGCACTGAAGAGAATCTCAGCAATCTGACAAGAAATGCTGAGATTATGGCCTATGC<br>CCTGGGAGTGGGAGCAGACCTGGTGAAGGGACTGATCATTGGAGATCAGAAGGAA<br>GGAGACTCCGGTGTCATCCAGGATGAGGAGGGAATGGATGAGCCCAACAACATGG<br>AGGCCCAAGATTGGCTGGAGTACATGGCCTCAAAGGGATTCAAGCTGACTCCAAAT<br>ATGGAGCTGCAAGTCCGTCACATGTGCCTGCGAATCACCAATCCCAGAAAAGCCAC<br>TCTGGGGAGTTACCTGCGGGAGAGATACAGTTCATCCCTGTGAGTTGAAGCAGAAA<br>TGAGTCATTTTTAGAACTATTATATTTTTATCCTAATAGCCTTAAATAGTTAGTCATC<br>TTGTGCTTAGATATTATGCCCTAGTTATATGAAAAAAACTTTAACAGGGATAAACAT<br>AGTTTCTACGGCTCTATTTTATCTCTCACATCTTAATTAACATGTTGTCATCTAACAA<br>AAACAAAAACAAATTCGACTTAGAGGGGATACAATTGCTGGCAAAAGGAGTCAAA<br>AATGCAGGAGAATCCATAGATAAAGCCACAGATGAGAAGCCTGAACATCTCTCTG<br>ACGCATTCTCAAAATACACAGAATATTTGTCCAATGAGAGCAAGGGGGAGGAGGA<br>GGATTTCCAATTCGAACAGGTGGACTATGGATTTCAAGAGTCACCTGAATCCAATA<br>ACCCTCTGCAAGAGAGCTGGATAGCGAAAGATGACCTGGAACCAGATATGGGAAA<br>GTCCTGGGTAGAGTATCAAGCTAAGATGTCCTTTGATTACAGTGAGCAGGTGAAAC<br>CCACTGTGATGAGGGAGATCAACGGCTTGCTGGGTATGCTGGGTGGATTTGCTAAA<br>TTCCAAGATGGAAAGAAAGAATATCTTTTCTATCTTCCAGAGAAGAAAGAGTCAGA<br>AGAGAGAAGGTCTGATAAGAAACAATGTCCTTTCAAGGTTGATGTCACCCCAAAAC<br>AAGACCCAAAAATCACTCCCACACCTGAGAAAGAGCCAAAACATCTGCCCACATC<br>AGCTGCCGGCAAAGAAATCACCGAAGAAGCAGCCGTGATGCAAGGATTCTGGATG<br>GACGGAATGAGGCTGACAGAGAAGACATCAGGGAAGTATTGCCTGTTCTTCCCACA<br>AAAAATGGGATGGTCTCAAGCCGAATGGATCAGCAAATCAGAAGACATTAATCCC<br>CGAACTCTGGCACATGACATCTTCAAATGGATGGTATCCAAATCTCCGAAACGGGC<br>CACATACTTGAGGAAATACATGGTAGAAGAGTAACCACAGGTCTGAACCTCTTGAC<br>TATTTTTGATGCCTTAAATTGGCTTGACGTACACGTATTACTTATTACATATCTCAGT<br>TATTTATATGAAAAAAATGTTAACAGACGTCAATAACATTTGTTATATGCGCTATCA<br>TCGTGAGACTATATTCAAACGTCGAATCAATTGAAGTTAAATGGCTCTATCTATATT<br>TAAGAAGAAGGGGAAGAAGACAGAATCTCCCAAGATGTTTCTGATGGATGCAAAC<br>CAGATGACCCCGAGTGCTCCACCCCCTTACCAGGCAGAACCTGGTCCGTTCGACAC<br>TTGGGGGAATGAGGAACTAGAAGAAGCTATGAAAGTGTGCTACTTAGTAGACACCT<br>GCTTGTCTGTGACAACAAGAGAACCTATTCGATCCGTGGTGGATGCCTATATCATAG<br>CACAAGGAGTCTTAGATCATTATACCGGCCCGATCCTTACCCGTCCGTTTTACATTG<br>CCTTGTTCTTGGGCGGGATCCACGGGATGCAAGCGGGAGTTAAAGGAGCGAGAAG<br>CATCCTGTATGAAAGAGAACATCATGGACCTCTGGTGTTCCCATATCACAGAAGCA<br>ACCCGCTAGATGGGACTCCCCGGGCTATTGAATTCCAGTACACCACTAGTCTGCGG<br>GGGAAGCCGGTAGAGGTGAGCTTCCAGGCCCGTCTGCAAGCTACCAGACAATTTGG<br>ACCTGGCGTAGAGGTTTATCTGGATGGCCTGCAAAGGATAGAACGCCCTGGCAACG<br>AACTAGTTCTAAAGCAATTCAGAGTCCCCTTGCTGATGCTGGAGAAAGGGAAATGG<br>GTGTTCAATCTTGATTCTGAGGGCAGTTACAATTAAAAGGGTTCAACAATCACATCA<br>TCTAAGTCACTTTTGCTATAATTATACGGTCAGGTTTACCGCCACAAAAGGGTTGAG<br>TAGCATGTAGACACATATAAATGCATCATATTATTAATTAATTAATTGTTAAGTTAT<br>ATGAAAAAAACTAACAGTGATACACCATTGTTTTGAGACATTTGTCACTGTGAAATT<br>CAGTTGGTTCAATAGAGACATGGATACTCTGATTAAAATTCTGCTGATTATAGTCAT<br>TCTAAAGTCTCTCCATGCTCACATCGAATTCGTGCCACATGATCTGAGTAAATGGAG<br>AGACATCAGCATAGAGCATCTGGATTGTCCAATCTATGGAGATCTGTCTAATCAAG<br>CAACTAGAACAACCCCTGTGAAATACAGCAGTGTTCAATGGGGCTTAAAAAATAA<br>CATTGATGGGTACCTTTGTATATCTGCCAAATGGTCGGTCACTTGTGATTATAGGTG<br>GTATGGCTCCAAATACATTTCAACCTCAATTGAATATGTTCCAACCAAAGAATCAG<br>AATGCAGAGATGCCATCAAGTCATCAAAAAAWGGAGAGTTAGTTAATCCCCATTT<br>CATGCCCGAAAATTGTGGATGGAACAATGTGTTAACGGAGAGTGTGACATACACA<br>ACTGTATCATCTCCCGAGGTAAAATTAGATCCATACCAGATGACTTTCGTGGATTCA<br>CTCTTTCCGGGCGGTAAATGCTCCTCTTCAGTGTGTAGCACCATTTATCATCAAGGA<br>GTCTGGATAAACCCGAGCAATAACTTAGGGTTTTGCAAAGATCCGGTCGATCATCA<br>AGGTCAGTTGTACATGGCTGGTCTTGTTGGAGCTCACGGAGAGATTGTGAAAGAAG<br>TTCGGAATCTCAGATCTGTGTTCAAGCCAGAAATTGGTAGATCCAAGCATTTGACTG<br>GATCTTGTTGGATAACATACTGTGATCAAAGAGGACTGAGATTTTCAGATGGAGAA<br>TGGACAGGTTTCCAAATTCCAGAGATCTCAGCACTGAAACAGGTTCTGCTAGGACT<br>TCCTGAGTGCAAAGATGATGTGCTGGTTCATGCTCATGACACGAACACTGAGTTGA<br>GGGAAATTTTGGAACACATGGACGAGTCAGCATTGAATGCCATCTGCCAACAAGA<br>AGTCCGCAGGGCAAAAGAGAGAGGAGTGGTTTCTGATTGGCTATTGAGTATGATGA<br>CACCATTCACAGAGGGGTTGGGGCCAGTTTATCGGCTGAACAAAGGAAAACTAGA<br>AGCATCAATGGGATATTATAGAAAGGTATATATAGACTCTAGCAACGCCCCTCAGG<br>CCTTCGGACAGACAGAAGATAAAGAATCTGTTGGATGGTCGGATCTTGTACCAAAA<br>GATGCAGATGGAGCTATATCATCGATGTACAATGGGAATGTCGTCATCAATAATCA<br>GATAAAATGGGCTAAAAATGCACTAGGATCTCATATATTAGATGAGATTTCTGCCTT<br>AGAATTTGAAACCCCCGTTGTGCATCATCCGCATTTGACAATCTTGTCGGTAAATCA<br>TAGTGATCTAGTGAGTTCCACACATCCAAATGGGCAAGGAGTTAATCTGATTGAGA<br>GTGTATCTCACTGGGCCGGTGGGTTGTGGGCATCCATAGGATCTGGGTTGATGATCT<br>TGGTTCTAGTGGCTTTAGTCGGATTTTGTACCATCAAGGTATGCTTAGCTTATGTTCC<br>ATCGATTTGGGCACAAAAAAATCGGAATGGTAAGAGAAGAGGAACAACATCCCAA<br>CGATCAACAGAACAGGAGATGTTCGAGCTATCAGCCGTGTAGGTACAAGTGATACT	[54]

	TCAGGTTAAATCGGAACTCTCATTTCTGTGTAATCAGATCTATATATTAAAAGACCT<br>ACTTAAAATATTGCATGTGTTACTGATGTCAGCGTAATAAGCTGAATCATAATTTAC<br>TTCTCATGATAATTTTGTTAACCTACATGTTTCCATCTTATTACATTTTATTCTTATTTT<br>TATTTTTATGAATATATATGAAAAAAACTATTCAACAGTCATCATGTATGACGAAGA<br>TCATTCAAGAGGATATGAATCCGATGACCATTATGACCTGCCTGAATGGCTGGAAG<br>AGGATATACAATCAGGAAATCCACTCAATCAAAAAGACTATAGCTTAAATTCTCCT<br>CTCATTGTAGATCTCACAGAAGCGCTGGTAAAATATCTAAATCAAGGTAATATAGA<br>GAGACGATTCCTCAGACATCAAGACAGATTTGAGAACATACAAAGTGAAATCCGC<br>AACGTTGCATGGAAGAATCCATCGAGCTCAAATCACAGATGGTGGGGGAAGTGGG<br>CTCAACAAACAGAAAAGAGTCCAGAGTTTGTAAGGCTGTTGTCTGATGTTAACCAG<br>GACATAGAAGAGACTAGTGACGTCTTGATGTCATTCTTGAAAGGATGGATCCAAGA<br>CACAACCTCTGTTCCAACAAAATTAAATTGGACATCTACTCAATTGAGCTATGGATC<br>CAAATTCTTCTTCATGCACAAATTGATCCTGTTCATGAATGCGCAATCTGATGAGGA<br>GAGGGTTATTCTCCAAGGGCACATCAAAGTCTCGGAAACAAAAAAGACTGGGATC<br>TACAAAGGTTCTCATCCTAGCTTAGGAAATTTTGTGCTGACCTCTGAATTCTTACTAC<br>TAGAACGACACAGAGTGATACTAGACCGTTCTTTCCTGTTGATGGTTAAAGATACTC<br>TAGTAGGGAGATTTCAAACACTCGCTAGCTTCATGAATAGGGAAGACAAAAAATAT<br>CCAGAAGACGTGATAGAAAAGGTGGAGACCCTCTATTCCTTAGGAGATCAATTAGT<br>AGAGGATCTAGGGGATGAAGCATATTCGGGGATAAAGTTGCTAGAACCAGCCTGC<br>AACCTCCGACTCGCAGAGTTGGCTAGAGAATTTAGGCCTTTGATCCCCGAATTCCCA<br>CACTTTCGAAACCATGTAGAGACAGCAATAGCAGAGGAGTCTGTCTTTAGTCCTGG<br>AATCACAGAATTTTTCAACCATGTAAATAAAGAAACAAATGTTGAAATCATCTTGG<br>CCTACTTCAGCTCTTTCCGACATTGGGGACATCCATACATTGATTATTTCCAAGGGTT<br>AATCAAATTGAACAAACAAGTCACTCTAGAAAAAGACATTGATACAGAATACGCT<br>AATGCACTGGCTAGTGATTTGGCCTACATGATTCTTCGAGGACATTTTAACACTAAA<br>AGAGTCTGGGCGGTAGACAAATCACTTGTGTCAAAGCAACACCCGTTGAGTGAGCA<br>TATCTTCAATGCCACTTGGCCAACTCCAAAACAGATTGATGACTTCGGAGATCACTG<br>GCATGAATTGCCTCTGATCAAAATTTATGACATCCCAGATTTAATTGACCCATCTGT<br>CATCTACTCTGATAAAAGTCATTCAATGGGGAGAGAAGAAGTGTTAAAACATGTTC<br>AAAGGAATCCAACACAAGCAATTCCTACAAAAAAGGTCTTAGAGACACTCTTACA<br>AAAACCTGCCACAAATTGGCCAGAATTCTTGTCCTCGATTGAAAAAGATGGCCTAC<br>CTAAAGACAGCTTGATTATCGGACTGAAGGGAAAAGAGAGGGAACTAAAAAAAG<br>CTGGAAGGTTCTTTTCTCTAATGTCCTGGGAATTGAGGGAGTATTTTGTAATAACTG<br>AATACCTGATCAAAACTCATTACGTCCCTCTCTTTAAAGGATTGACCATGGCAGATG<br>ACATGACAGAGGTCGTCAAGAAGATGTTAGAAAGAAGTCAAGGACAAGGAGAGG<br>ATGATTACGAGCATGTCAGCATTGCCAACCACATTGATTATGAAAAATGGAACAAC<br>CATCAAAGGAAAGAGTCTAACGGCCCAGTATTCAGAGTCATGGGACAATTTCTGGG<br>GTACCCTAGTTTGATAGAAAAGACCCATGACTTCTTTGAGCAAAGCTTGATCTATTA<br>TAATGGCAGACCTGACTTGATGCAGACAGACGGAGATGAGTTGCAAAACCGAACG<br>GAAGCTTTGGTGTGTTGGAATGGTCAGAAAGGAGGATTAGAAGGACTCAGGCAAA<br>AAGGATGGAGCATCCTCAATCTGTTGGTCATCAAAAGAGAATCTAAAATCAGAAA<br>CACTAAAGTGCAAACTTTGGCCCAGGGAGACAATCAGGTAGTGTGTACCCAATACC<br>GCATTATGCCAACCAGATCGACACTTGAATTGCAGGCAGAACTTGAAAAAGTTAAG<br>AAGAACAATCAAGTTATTATGGATGCTATTGAGACAGGGACTAACAAATTAGGGCT<br>ACTGATAAACAATGATGAAACCATTCAATCCGCTGATTTCCTAACCTATGGTAAGG<br>TTCCAATATTTAGAGGTAATATTCGCTGTTTGGAAACTAAAAGATGGTCTAGAGTGA<br>CTTGTGTCACTAATGATCAATTGCCATCCCTTTCCAATGTCATGTCATCTGTGTCGAC<br>AAACAGTCTGACGGTCTCGCATTTCGATGTCAGTCCTATAGAATCCATGAGACAAT<br>ACCTGTTCTTCGGAAATTTTGCTCGGAGATTGGTGGAATTCCACAATCCTGCGATGA<br>GAGTGCCGATTAGTCTAGAAGACCTGGATTCCAAACAAAAGTCTGTTTATCTGAAC<br>GCAGTGCTTTTCTTGGACCCATCTCTGGGTGGCGTGTCTGGTATGTCATTGTCAAGAT<br>TCTTGACTAGGATGTTTCCTGATCCCATCACAGAAGGATTATCTTTTTGGAAAATCG<br>TTTACGAACATACCACTTCCAAAGACACTCAACTGCTCTGCAGAATCGCTGGAAGC<br>CCCGAATTGGCTAGACGCCAGAACAATCTGGACAAGTTGATAGAGAATCCAACAG<br>CATTGAATCTGAGCAAAGAAACTTCTGCCCTAAGTGTCATTAAGAAAGAAGTCAGG<br>TCACGCCTGTACAAGGATTGCGACAAATTCAAGAACAAATTGATTGCAGATGCAAT<br>TGGGATTGCTAGAGATGAAGAGGCTCACCTGGAATTATTCTTAATGTCAATCCGAC<br>CATTATTTCCGAGATTCCTGGCAGAGTTCAAAGCAGCAACATTTGTTGGCATCACAG<br>AATCTCTCATTTCTTTATTCCAAAATTCCAAAACCATTCGCAACATCTTCAGGAAGA<br>AATATGCGAAAGAATTGGAACTTAGAGTAGTGCAGTGTGAATACAGATCCATCAAT<br>TTGATGTTGAGCCTCGCAGATCGATCCCACTTAGATGAAATGTGGACATGCTCGGC<br>ATCTAAAGCAGACGAACTCCGAACCTTATCATGGGGGACTACAATAATTGGAACA<br>ACTGTCCCTCATCCTTTAGAGATGATCAATCATGCTCATATAGGACAGAAATGCGA<br>CTCGTTAGAAACCCTGGACTACATAAATGTCACTGTGGTTCAAGATCTGACTGATTG<br>TCTAACTAGCAAAGGGAAGTTACCAGCGTACTTAGGATCCAAAACATCAGAGACT<br>ACAAGCATCTTGCAACCATGGGAAAAGGAGACAAAGATTCCTGTGATTCGTAGAG<br>CTGCTAAATTGAGAGCTGCAATCACCTGGTTTGTAGAGCCTGATTCTCTTTTGGCAC<br>AAAGCATCCTGAACAACATTGAAAGTCTGACTGGAGAAGATTGGTCTGCTTCAATA<br>TCTGGATTCAAAAGGACAGGATCCGCACTACATCGCTTCACTAGTGCTAGAGTGAG<br>TGCAGGAGGCTTTTCAGCTCAAAGCCCGGCCAGATTGACCAGGATGATGGCAACAA<br>CAGATACTTTCCGAGAAATTGGGTCAGATAATTACGATTTTATGTTCCAATCATTAC<br>TACTCTTTGCCCAAATGACTACAGGTGAGATTTACAAAAGGAGTCCAGCTACTAATT<br>TCCACTTTCATCTGAGTTGCCACCAATGTCTTCGTAAGATCGAAGAGCCTACCTTAA<br>ACTCTGATTTTGCCTACAACCCTATTCATAGATCGGACATCTTGGATAAGTGGAAAC<br>CTCAGACCACAGATTGGTCTTCAGAAAGAAAGGCTCCAGAGATAGAGGAAGGGAA<br>TTGGGACAGATTGACACATCAGGAGCAAAGTTTTCAGATAGGGAAGTCCATTGGGT<br>TTCTGTTTGGAGATTTGACAATGACAAAGAATTCACATGCTCAGGATTCTTCTATTTT<br>CCCTTTATCAATTCAATACAAGATCACAGCTGCGGAGTTTCTGGAAGGAATTCTAGA<br>TGGGATAGTCAAGGCTTCTGCTTTGTCTACTATCCACCGGAGGAACTTCGATCATCA<br>CAGCAAGTACAAGTCCACTGTTTCAGGAACTGTTGATTATTTAATCGAATTGATTTC	

	AGAGTCCGCGGGGTTCACAAATCTGACCAGGAATGGTCCCTTGAAGGCTTGCTTAC<br>TGACCATTCCACATAAGATACCTCCATCCTATCCTCTCAGCCAATCTGATCTTGGTG<br>CTATGTCAAGAAATTACCTCAGACTGTTGCACCGAAGAATGTCTACCGGCACCTAC<br>AAGACCAGGTGGCCGACAAATTGGATATTCTCTGATATGATGAGCCCTAACATAAT<br>CTATCCATTTGTGATCAGTGTCTCCTGCGTTGGACTGGCTTACTCATCATCTTGGACA<br>AAGAAATCTGCTGACAAATTGAGAGGGTTAAGAGGAGTAGCTGAGTTGATTCGATC<br>ATCAGATGATGTGCAATTGCCTGTTGGGAAGCTATTCAAAACAGTAAACCAAGAAA<br>TTAGACATGCCATAAAACATCATGCATCGGACGATGCCGAAATCCCCGAAAGCCA<br>CATCCAAGAAGGCTGGAAAAAAGAGCTCATAGTCAACATTAACATGCAGCCAATT<br>GACTATAGTAGAACAGCGACAGCGAAGTCGTTAGACCGGCCTGCTCAAATTCGAG<br>ACCCTTTAATATCTGGACTAAGAACAGCACAATTGGCCACTGGATCCCACTACAAG<br>TTACGATCCATACTAGTGCAAAACCGAATCCAAGTTACCGATGCTCTGTGCGGAGG<br>GGATGGATCTGGTGGAATCGGGGCTTGTGTCCTGAGACAATACCCGTTTGCTAAGCT<br>GATTTACAACAGTCTATTCGAGATTCAAGATCTGGATATGAGGGGGAGTGCACCTG<br>GACCGCCATCTGCTATTGCCGCAATGGGAAACATGAGTATGAGGTGTGTCAATCGA<br>GATTCTGCATGGAAAAATCCATCTGATCTGAGCCACACTTCCACCTGGGAATATTTT<br>CAATCGTTGATGACCCAACATCAGTTGCGGTGCAATCTGTGGACCTTTGACATGGA<br>GGTTCGCAGCCATGACATTTCAGATGCTATAGAAAAGCAAATAGTGGCCAATCTTC<br>ATTTGCTACCAAAAAATGGGACAATCATATACAAGACATATCTAACGAAATTGTCT<br>GACATGGAGACAACAATCTTGGACAGACTGGGTGGATTCTTCAAAAGAGTCAGTCT<br>TGTGTCTACAGACGCTACATCAAGCCATAGCTCTGAAGTCTACGCTGTATTCCAAAA<br>CAAACTGGATAAGAGACAATTGGAAATCCACCCCAACTGGTCTTCCTGCAATTTAG<br>GTTCTGACATACACCCATGCTGGAAAAGTGAAGATGAGGAGTTTGAAAGGGCAAG<br>AAGATTCTTTCACATGAAGAGACAACAAGGGGTGCCGACTCGATTGAGGCCTACTT<br>TGGATTCCGAAATCCAGGTCCTGAGCGTCTCGGCCGGAGTTGAAAACGGGGTAGCA<br>ATGACATTAGCTATGGACGTAAGCAATCAGGTCTCGGATCCAACCACTGGGGCATT<br>TCTTTGGCTTTTAGTAACTTTGCAACATATATGCCCAATAGGCCCATCATTCAAGAT<br>CCCATCCAGTTCTGCAGTTGAATCTTATTTGGCCATACTGATTGGATTCAGTAGTATC<br>TACCAGCTTCAGACAGGCAATAACAAAGCTTATGCCAGCATCAAGACCTGCTTGTC<br>TCAATCTGCTCCATTCTTCTGCAACCCTGATGGATGGAGTTGCATTAAAGGACTTGA<br>CAAGTCTATGCGGATGGACAGAAAGTTGGCTTTAGTGGGCAGTGTGATACGGGCAT<br>GGTCAAAGTGGAACTTGACTCAGTCAATCAACTTTCACAAACTAGATGGTATGATG<br>AGACATTATCTACCGAAGGGAACATTGAGGCAGATTGGCTACAAAACCGGGATCT<br>GGGACTATATCAATGGGGCAGTGAAAGGAGTACGAAGTGCTGCCAATCAGGACAG<br>TCCAAAAGAGTCAGCAGCTGCTTGGAGAGATTAGTATATATAACATTGTGTCTATC<br>ATCCAATTCATTATTAGTAGGTGACAATTAGTTAAAGAACAAGATTACCCCATGTA<br>AGAGAATTTTTTATGTCCTAATTTGCCGTATGCACTTGTAMATAAATGTGTATTGTA<br>ATGTATATGTGTATGTGTATTTGATGTATTGGTGTATATGTCAACAAATCAATAATAT<br>GATCACATAGGATAAGGTCAAGACCAATTGTTTAAGTTTTATTAGAATTCCGTGAAT<br>ATGAAAAAAACAAACAGGCCC	
KC608037	TAAGGCTATTCTTTAACAGACATCTGTTTGTTTCATTGAAAATGGCTGCCATGAACT<br>CCATCAAAGTTACAGTTAGAGCCAATGGACAAGATTTCACCCCAGTTTGTCCTAAA<br>ATGGAGGATGAAGTGGCTTACCCATCTGATTATTTAGATGCTAATGGATTACCAACC<br>TTCCAGCTCTACTATCATGATCTGTCGAAAAAGGATCTGCTAGACCTGATCTGGGGA<br>GAAGCATCAGAGGCAAGGCTGCCTAGTGAGCTGGTGACCGCGTACGTGTACAAGG<br>TGGTGAGTGAGTGGAAAGAGATTCTGGAGAGTGATTGGTCATCTTTCCGATTCCAG<br>ATTGGCAAAGCGGGAGAGGAGATAACCCCATTCAACCTGATTGGCATGACTGTGA<br>ACAGTCAGAAACTAGCTGACTACAAGAAAGCAGTTGCTCCTGAGGGGATAGATGA<br>GGTGGCAATGGTGATCTATCTCCTGGCACCATACCGGATAGTCGGAATTAAAAACG<br>AAGACTATCAAGATCGGGTGATCACCAACATCCAGAACCAGCTGGACAGTCTCGG<br>CGCAAAGAAGCTGCAGGTGAAGGCACTGAAGAATGTCACTACTCTCATCAACAGT<br>GCCAACTATCTGAGAATGGTGGCAGTGATTGACATGTTCTACTACCATTTCAAGAAC<br>AGTCAAGAAAGAGCTGTTGTCAGGATTGCCACTCTGAGTTCCCGTCACAAAGACTG<br>TGCAGCTCTATCCACCCTGAATCACATCACCAGCTTCACTGGGAGGAGTTTTGTGCA<br>GGTGTTGGATTGGGTTTTCACAGATCAAGTTGCCAAGGAGATCGGCAGGATGATGC<br>GTGCGGGTCAAGAAATTGATCGTCCTGAATCCTACATGCCGTACCTGAAGGACTTG<br>GGGCTGAGCAGAAAATCCCCCTACTCCTCTTCAGCAAATCCTGGCACTCACTGCTG<br>GGCACAGATGGTATGTGCCATGATGGGATCCAAGAGATCTCAAAACGCAATTGCCA<br>GCACTGAAGAGAATCTCAGCAATCTGACAAGAAATGCTGAGATTATGGCCTATGCC<br>CTGGGAGTGGGAGCAGACCTGGTGAAGGGACTGATCATTGGAGATCAGAAGGAAG<br>GAGACTCCGGTGTCATCCAGGATGAGGAAGGAATGGATGAGCCCAACAACATGGA<br>GGCCCAAGATTGGCTGGAGTACATGGCCTCAAAGGGATTCAAGCTGACTCCAAATA<br>TGGAGCTGCAAGTCCGCCACATGTGCCTGCGAATCACCAATCCCAGAAAAGCCACT<br>TTGGGGAGTTACCTGCGGGAGAGATACAGTTCATCCTTGTGAGTTGAAGCAGAAAT<br>GAATCATTTTTAGAACTATTATATTTTTATCCTAATAGCCTTAAATAGTTAGTCATTT<br>TGTGCTTAGATATTATGCCCTAGTTATATGAAAAAAACATTAACAGGGATAAACAT<br>AGTTTCTACGGCTCTATTTGATCTCTCACATCTTAATTAACATGTTGTCATCTAACAA<br>AAACAAAAACAAATTCGATTTTGAGGGGATACAATTGCTGGCAAAAGGAGTCAAA<br>AATGCAGGAGAATCTATAGATAAAGCCACAGATGAGAAGCCTGAACATCTCTCTG<br>ACGCATTCTCAAAATACACAGAATATTTGTCCAATGAGAGCAAGGGGGAAGAAGA<br>GGATTTCCAATTCGAACAGGTGGACTATGGATTTCAAGAGTCACCTGAATCCAATA<br>ACCCTCTGCAAGAGAGCTGGATAGCGAAAGATGACCTGGAACCAGATATGGGAAA<br>GTCTTGGGTAGAGTACCAAGCTAAGATGTCCTTTGATTACAGTGAGCAGGTGAAAC<br>CCACTGTGATGAGGGAGATCAACGGCTTGCTGAGTATGCTGGGTGGATTTGCTAAA<br>TTCCAAGATGGAAAGAAAGAATATCTTTTCTATCTTCCAGAGAAGAAAGAGTCAGA<br>AGAGAGAAGGTCTGATAAGAAACAATGTCCTTTCAAGGTTGATGTCACCCCAAAAC<br>AAGACCCAAAAATCACTCTCACACCTGAGAAAGAGCCAAAACATCTGCCCACATC<br>TGCTGTCGGCAAAGAAATCACCGAAGAAGCAGCCGTGATGCAAGGATTCTGGATG<br>AACGGAATGAGGCTGACAGAGAAGACATCAGGGAAGTATTGCCTGTTCTTCCCACA<br>AAAAATGGGATGGTCTCAAGCCGAGTGGATCAGCAAATCAGAAGATATTAATCCC	KC608037.1 Eel virus European X isolate DF25/04, partial genome [54]

	CGAACTCTGGCACATGACATCTTCAAATGGATGGTATCTAAATCTCCGAAACGAGC<br>CACATACTTGAGGAAATACATGGTAGAAGAGTAACCACAGGTCTGAACCTCTTAAT<br>TATTTTTGATGCCTTAAATTGGCTTGACGTACACGTATTACTTATTACACATCTCAGT<br>TATTTATATGAAAAAAATGTTAACAGACGTCAATAACATTTGTTATATGCGCTATCA<br>TCGTGAGACTATATTCAAACGTCGAATCAATTGAAGTTAAATGGCTCTATCTATATT<br>TAAGAAGAAGGGGAAGAAGACAGAATCTCCCAAGATGTTTCTAATGGATGCAAAC<br>CAGATGACCCCGAGTGCTCCACCCCCTTACCAGGCAGAACCTGGTCCGTTTGACAC<br>TTGGGGGAATGAGGAACTAGAAGAAGCTATGAAAGTGTGCTACTTAGTAGACACTT<br>GCTTGTCTGTGACAACAAGAGAACCTATTCGATCAGTGGTGGATGCCTATATCATA<br>GCACAAGGAGTCTTAGATCATTATACCGGCCCGATCCTTACCCGTCCATTTTACATT<br>GCCTTGTTCTTGGGCGGGATCCACGGGATGCAGGCGGGAGTTAAAGGAGCGAGAA<br>GCATCCGGTATGAAAGAGAACATCATGGACCTCTGGTGTTCCCATATCACAGAAGC<br>AACCCGCTAGATTGGACTCCCCGGGCTATTGAATTCCAGTACACCACTAGTCTGCG<br>GGGGAAGCCGGTAGAGGTGAGCTTCCAGGCCCGTCTGCAAGCTACCAGACAATTTG<br>GACCTGGCATAGAGGTCTATCTGGATGGCCTGCAAAGGATAGAACGCCCTAGCAAC<br>GAACTATTTCTAAAGCAATTCAGAGTCCCCCTGCTGATGCTGGAGAAAGGGAAATG<br>GGTGTTCAATCTTGATTCTGAGGGTAGTTACAATTAAAAGGGTTCAACAATCACATC<br>ATCTGATCCACTTTTGCTATAATTATACGGTCAAGTTCACCGCCCCAAAAGGGTTGA<br>GTAGCATGTAGACACATATAAATGCATCATATTATTAATTAATTAATTGTTAATTTAT<br>ATGAAAAAAACTAACAGTGATACACCATTGTTTTGAGACATTTGTCACTGTGAA<br>ATTCAGTTGGTTCAATAGAGACATGGATACTCTGATTAAAATTCTGCTGATTATAGT<br>CATTCTAAAGTCTCTCCATGCTCATATCGAATTCGTGCCACATGATCTGAGTAAATG<br>GAGAGACATCAGCATAGAGCATCTGGATTGTCCAATCTATGGAGATCTGTCTAATC<br>AAGCAACTCGCACAACCCCTGTGAAATACAGCAGTGTTCAATGGGGCCTAAAAAA<br>TAATGTTGATGGGTACCTTTGTATATCTGCTAAATGGTCAGTCACTTGTGACTAT<br>CGGTGGTATGGCTCCAAATACATTTCAACCTCTATTGAATATGTTCCAACCAAAGAA<br>TCAGAATGCAGAGATGCCATCAAGTCATCAAAAAAATGGAGAGTTAGTTAGTCCCCA<br>TTTCATGCCCGAAAATTGTGGATGGAACAATGTGTTAACGGAGAGTGTGACATACA<br>CAACTGTATCATCTCACGAGGTAAAATTAGATCCATACCAGATGACTTTCGTGGATT<br>CACTTTTCCCGGGCGGTAAATGCTCCTCTTCAGTGTGTAGCACCATTTATCATCAAG<br>GAGTCTGGATAAGCCCGAGTGACAACTTAGGGTTTTGCAAAGATCCAGTCGATCAT<br>CAAGGTCAGTTGTACATGGCTGGTCTTGTTGGAGCTCACGGAGAGATTGTGAAGGA<br>AGTTTGGAATCTCAGATCTGTGTTCAAGCCAGAAATTGGTAGATCCAAGCATTTGAC<br>TGGATCTTGTTGGATGACATACTGTGATCAAAGAGGACTAAGATTTTCAGATGGAG<br>AATGGGCAGGTTTCCAAGTTCCAAAGATCTCAGCACTGAAACAGGTTCTGCTAGGA<br>CTTCCTGAGTGCAAAGATGATGTGCTGGTTCATGCTCATGACACGAACACTGAGTTG<br>AGGGAAATTTTGGAACACATGGACGAGTCAGCATTGAATGCCATCTGCCAACAAG<br>AAGTCCGCAGGGCAAAAGAGAGAGGAGTGGTTTCTGATTGGCTATTGAGTATGATG<br>ACACCATTCACAGAGGGGTTGGGGCCAGTTTATCGGCTGAACAAAGGAAAACTAG<br>AAGCATCAATGGGATATTATAGGAAGGTATATATAGACTCTAGCAACGCCCCTCAG<br>GCCTTCGGACAGACAGAAGATAAAGAATCTGTTGGATGGTCGGATCTTGTACCAAA<br>AGATGCAGATGGAGCTATATCATCGATGTACAATGGGAATGTCGTCATCAATAATC<br>AGATAAAATGGGCTAAAAATGCACTAGGATCTCATATATTAGATGAGATTTCTGCC<br>TTAGAATTTGAAACCCCCGTTGTGCATCATCCTCATTTGACAATCTTGTCTGTAAATC<br>ATAGTGATCTGGTGAGTTCCACACATCCAAATGGGCAAGGAGTTAATCTGATTGAG<br>AGTGTATCTCACTGGGCCGGTGGGTTGTGGGCATCCATAGGATCTGGGTTGATGATC<br>TTGGTTCTAGTGGCTTTAGTCGGATTTTGCACCATCAAGGTATGCTTAGCTTATGTTC<br>CATCGATTTGGGCACAAAAAAATCGGAAGAGAAGAGGAACAACATCCCAACGATC<br>AACAGAACAGGAGATGTTTGAGCTATCAGCTGTGTAAGTACAAGTGATACTTCAGG<br>TTAAATCGGAACTCTCATTTCTGTGTAATCAGATCGATATATTAAAAGACCTACTTA<br>AAATATTGCATGCATTACTGATGTCAGCGTAATAAGCTGAATCATAATTTACTTCTC<br>ATGACAAGTTTGTTCACCTACATGTTTTCATCTTATTACATTTTATTCTTATTTTTATTT<br>TTATGAATATATATGAAAAAAACTATTCAACAGTCATCATGTATGACGAAGATCAT<br>TCAAGAGGATATGAATCCGATGACCATTATGACCTGCCCGAATGGCTGGAAGAGG<br>ATATACAATCAGGAAATCCACTCAATCAAAAAGACTATAGCTTAAATTCTCCTCTC<br>ATCGTAGATCTCACAGAAGCGCTGGTAAAATATCTAAATCAAGGCAATATAGAGA<br>GACGATTCCTCCGACATCAAGACAGATTTGAGAACATACAAAGTGAAATCCGCAA<br>CATTGCATGGAAGAATCCATCAAGCTCAAATCACAGATGGTGGGGGAAGTGGGCT<br>CAACAAACAGAAAAGAGTCCAGAGTTCGTAAGGCTGTTGTCTGATGTTAACCAGGA<br>CATAGAAGAGACTAGTGATGTCTTGATGTCATTCTTGAAAGGATGGATCCAAGACA<br>CAACCTCTGTTCCAACAAAATTAAATTGGACATCTACTCAATTGAGCTATGGATCCA<br>AATTCTTCTTCATGCATAAATTGATCCTGTTCATGAATGCGCAATCCGATGAAGAGA<br>GGGTTATTCTCCAAAGGCACATTAAAGTCTCGGAAACAAAGAAGACTGGGATCTAC<br>AAAGGTTCTCATCCTAGCTTAGGAAATTTTGTACTGACATCTGAATTCTTACTACTA<br>GAACGACACAGAGTGATACTAGACCGTTCTTTCCTGTTAATGATTAAAGATACTCTA<br>GTAGGGAGATTTCAGACACTCGCTAGCTTCATGAATAGGGAAGACAAAAAATATCC<br>AGAAGACGTGATAGAAAAGGTGGAGACCCTCTATTCTTTAGGAGATCAATTAGTAG<br>AGGATCTAGGGGATGAAGCATATTCGGGAATAAAGTTGCTAGAACCAGCCTGCAA<br>CCTCAGACTCGCAGAGTTGGCTAGAGAATTTAGGCCTTTGATCCCCGAATTCCCACA<br>CTTTAGAAACCATGTAGAGACAGCAATAGCAGAGGAGTCTGTCTTTAGTCCTGGAA<br>TCACAGAATTTTTCAACCATGTAAATAAAGAAACAAATGTTGAAATCATCTTGGCC<br>TACTTCAGCTCTTTCCGACATTGGGGACATCCATACATTGATTATTTCCAAGGGTTA<br>ATCAAGTTGAACAAACAAGTCACTCTAGAAAAAGACATTGATACAGAATACGCTA<br>ATGCACTGGCTAGTGATCTGGCCTACATGATTCTTCGAGGACATTTTAACACTAAAA<br>GAGTCTGGGCGGTAGACAAATCACTTGTGTCAAAGCAACACCCGTTGAGTGAGCAT<br>ATTTTCAATGCCACTTGGCCAATTCCGAAACAAATTGATGATTTCGGAGATCACTG<br>GCATGAATTGCCTTTGATCAAAATTTACGACATCCCAGATTTAATTGACCCTTCTGT<br>CATCTACTCTGACAAAAGTCATTCAATGGGGAGAGAAGAAGTGTTAAAACATGTTC<br>AAAGGAATCCAACACAAGCAATTCCCACAAAAAAGGTCTTAGAGACACTCTTGCA<br>AAAACCTGCCACAAATTGGCCAGAATTCTTGTCCTCGATTGAAAAAGATGGCCTAC	

	CTAAAGACAGCTTGATTATCGGACTTAAGGGAAAAGAGAGGGAACTAAAAAAGGC<br>TGGAAGGTTTTTTTCTCTAATGTCCTGGGAATTGAGGGAGTATTTTGTAATAACTGA<br>ATACCTGATCAAAACTCATTACGTCCCTCTCTTTAAAGGATTGACCATGGCAGATGA<br>CATGACAGAGGTCGTCAAGAAGATGTTAGAAAGAAGTCAAGGACAAGGAGAGGA<br>TGATTACGAGCATGTCAGCATTGCCAACCACATTGATTATGAAAAATGGAACAATC<br>ATCAAAGAAAAGAATCTAACGGCCCAGTGTTCAGAGTCATGGGACAATTTCTGGGG<br>TACCCTAGTTTGATAGAAAAGACCCATGACTTCTTTGAGCAAAGCTTGATCTATTAT<br>AATGGCAGACCTGACTTGATGCAGACAGACGGAGATGAGTTGCAAAACCGAACGG<br>AAGCTTTGGTGTGTTGGAATGGTCAGAAAGGAGGATTAGAAGGACTCAGGCAAAA<br>GGGATGGAGCATCCTCAATCTGTTGGTCATCAAAAGAGAATCTAAAATCAGAAACA<br>CTAAAGTGCAAACTTTGGCCCAGGGGGACAATCAGGTAGTGTGTACCCAATACCGC<br>ATTATGCCAACCAGATCAACACTTGAATTGCAGGCAGAACTTGAAAAAGTTAAGA<br>AGAACAATCAAGTTATTATGGATGCTATTGAGACAGGGACTAACAAATTAGGGCTA<br>CTGATAAACAATGATGAAACCATTCAATCCGCTGATTTCCTAACATATGGTAAGGTT<br>CCAATATTTAGAGGCAATATTCGCTGTTTGGAAACTAAAAGATGGTCTAGAGTGAC<br>TTGTGTCACTAATGATCAATTGCCGTCCCTTTCTAATGTCATGTCGTCTGTGTCGACA<br>AACAGTCTTACGGTCTCGCATTTTGATGTCAGTCCCATAGAATCCATGAGACAATAC<br>CTGTTCTTCGGAAATTTTGCTCGGAGACTGGTGGAATTCCATAATCCTGCGATGAGA<br>GTGCCGATTAGTCTAGAAGACCTGGATTCCAAACAAAAGTCTGTTTATCTGAACGC<br>AGTGCTTTTCTTAGACCCATCTCTGGGTGGCGTGTCTGGTATGTCATTGTCAAGATTT<br>TTGACTAGGATGTTTCCTGATCCCATCACAGAAGGATTATCTTTTTGGAAAGTCGTTT<br>ACGAACATACCACTTCCAAAGACACTCAACTGCTCTGCAGAATCGCTGGAAGCCCC<br>GAATTGGCTAGACGCCAGAACAATCTGGACAAGTTGATAGAGAATCCAACAGCAT<br>TGAATCTGAGCAAAGAAACTTCTGCCCTAAGTGTCATTAAGAAAGAAGTCAGGTCA<br>CGCCTGTACAAGGATTGCGACAAATTCAAGAACAAATTGATTGCAGATGCAATTGG<br>GATTGCTAGAGATGAAGAGGCTCACCTGGAATTGTTCTTAATGTCAATCCGACCATT<br>ATTTCCGAGATTCCTGGCAGAGTTCAAAGCAGCAACATTTGTTGGCATCACAGAAT<br>CTCTCATTTCTTTATTCCAAAATTCCAAAACCATTCGCAACATCTTCAGGAAGAAAT<br>ATGCAAAAGAATTGGAACTTAGAGTAGTGCAATGTGAATACAGATCCATCAATTTG<br>ATGTTGAGCCTCGCAGATCGATCCCACTTAGATGAAATGTGGACATGCTCGGCATCT<br>AAAGCGGACGAACTTCGAACCCTATCATGGGGGACTACAATAATTGGAACAACTG<br>TCCCTCATCCTTTAGAGATGATCAATCATGCTCATATAGGACAGAAATGCGACTCGT<br>TAGAAACCCTAGACTACATAAATGTCACTGTGGTTCAAGATCTGACTGATTGTCTAA<br>CTAGCAAAGGGAAGTTACCAGCGTACTTAGGATCCAAAACATCAGAGACTACAAG<br>CATCTTGCAACCATGGGAAAAGGAGACAAAGATTCCTGTGATTCGTAGAGCTGCTA<br>AATTGAGAGCTGCAATCACCTGGTTTGTAGAGCCTGATTCTCTCTTGGCACAAAGCA<br>TCCTGAACAACATTGAAAGTCTGACTGGAGAAGACTGGTCTGCTTCAATATCTGGA<br>TTCAAAAGGACAGGGTCCGCACTACATCGCTTCACTAGTGCTAGAGTGAGTGCAGG<br>AGGCTTTTCAGCTCAAAGCCCGGCCAGATTGACCAGGATGATGGCAACAACAGAT<br>ACTTTCCGAGAAATTGGGTCAGATAATTACGATTTTATGTTCCAATCATTACTACTCT<br>TTGCCCAAATGACTACAGGTGAGATTTACAAAAGGAGTCCAGCTACTAATTTCCAC<br>TTTCATCTGAGTTGCCACCAATGTCTTCGTAAGATCGAAGAGCCTACCTTAAACTCT<br>GATTTTGCCTACAACCCCATTCAGAGATCGGACATCTTGGATAAGTGGAAACCTCA<br>GACCACAGATTGGTCTTCAGAAAGAAGAGCTCCGGAGATAGAGGAAGGGAATTGG<br>GACAGATTGACACATCAGGAGCAAAGTTTTCAGATAGGGAAGTCTATCGGGTTTCT<br>GTTTGGAGATTTGACAATGACAAAGAATTCACATGCTCAGGATTCTTCTATTTTCCC<br>TTTGTCAATTCAATACAAGATCACAGCTGCGGAGTTTCTGGAAGGAATTCTAGATGG<br>GATAGTCAAGGCTTCTGCTTTGTCTACTATCCACCGGAGGAACTTCGATCATCACAG<br>CAAGTACAAGTCTACTGTTTCAGGAACTGTTGATTATTTAATCGAATTGATTTCAGA<br>GTCCGCGGGGTTCACAAATCTAACCAGGAATGGTCCCTTGAAGGCTTGCTTACTGA<br>CCATTCCACATAAGATACCTCCATCCTATCCTCTCAGCCAATCTGATCTTGGTGCTAT<br>GTCAAGAAACTACCTCAGACTGTTGCACCGAAGAATGTCTACCGGTACCTACAA<br>GACCAGGTGGCCGACAAATTGGATATTCTCTGATATGATGAGCCCTAACATAATCT<br>ATCCATTTGTGATCAGTGTCTCCTGCGTTGGACTGGCTTACTCATCATCTTGGACAAA<br>GAAATCTGCTGACAAATTGAGAGGGTTAAGAGGAGTAGCTGAGTTGATTCGATCAT<br>CAGATGATGTGCAATTGCCTGTTGGGAAGCTATTCAAAACAGTAAACCAAGAAATT<br>AGACATGCCATAAAACATCATGCATCGGACGATGCTGAAATCTCCGAAAGCCAC<br>ATCCAAGAAGGCTGGAAAAAAGAGCTCATAGTCAACATTAACATGCAGCCAATTG<br>ACTATAGTAGAACAGCTACAGCGAAGCTGTTGGACCGGCCTGCTCAAATTCGAGAC<br>CCTTTAATATCTGGACTAAGAACAGCACAATTGGCCACTGGATCCCACTACAAGTT<br>ACGATCCATACTAGTGCAAAACCGAATCCAAGTTACCGATGCTCTATGCGGAGGGG<br>ATGGATCTGGTGGAATCGGGGCCTGCGTCCTGAGACAGTACCCGTTTGCTAAGCTG<br>ATTTACAACAGTCTATTCGAGATTCAAGATCTGGATATGAGGGGGAGTGCACCTGG<br>ACCGCCATCTGCTATTGCCGCAATGGGGAACATGAGTATGAGGTGTGTCAATCGAG<br>ATTCGGCATGGAAAAATCCATCTGATCTGAGCCACACTTCCACCTGGGAATATTTTC<br>AATCGTTGATGACCCAACATCAGTTGCGGTGCAATCTGTGGACCTTTGACATGGAG<br>GTTCGCAGCCATGATATTTCAGATGCTATAGAAAAGCAAATAGTGGCCAATCTTCA<br>TTTGCTACCAAAAAATGGGACAATCATATACAAGACATATCTAACTAAATTGTCTG<br>ACATGGAGACAACAATCTTGGACAGATTGGGTGGATTCTTCAAAAGAGTCAGTCTT<br>GTGTCTACAGACGCTACATCAAGCCATAGCTCTGAAGTCTATGCTCTATTCCAAAAC<br>AAACTGGACAAGAGACAATTGGAAATCCACCCCAACTGGGCTTCCTGCAATTTAGG<br>TTCTGACATACACCCATGCTGGAAAAGTGAAGATGAGGAGTTTGAAAGGGCAAGA<br>AGATTCTTTCACATGAAGAGACAACAAGGGGTGCCGACTCGATTGAGACCTACTTT<br>GGATTCCGAAATCCAAGTCCTAAGCGTCTCGGCCGGAGTTGAAAACGGGGTGGCA<br>ATGACATTAGCTATGGATGTAAGCAATCAGGTCTCGGATCCAACCACTGGGGCATT<br>TCTTTGGCTTTTAGTAACTTTGCAACATATATGCCCAATAGGCCCATCATTCAAGAT<br>CCCATCCAGTTCCGCAGTTGAATCTTACTTGGCCATACTGATTGGATTCAGTAGTA<br>TTTACCAGCTTCAGACAGGCAATAACAAAGCTTATGCCAGCATCAAGACCTGCTTG<br>TCCCAATCTGCTCCATTCTTCTGCAACCCTGATGGATGGAGTTGCATTAAAGGACTT<br>GACAAGTCTATGCGGATGGACAGAAAGTTGGCTTTAGTGGGCAGTGTGATACGGGC	

	ATGGTCAAAGTGGAACTTGACTCAGTCAATCAACTTTCACAAACTAGATGGTATGA<br>TGAGACATTATCTACCGAAGGGAACATTGAGGCAGATTGGCTGCAAAACCGGGAT<br>CTGGGACTATATCAATGGGGCAGTGAAAGGAGTACGAAGTGCTGCCAATCAGGAC<br>AGTCCAAAAGAGTCAGCAGCTGCTTGGAGAGATTAGTATATATGACATTGTGTCTA<br>TCATCCGATTCATTATTAGATAGGTGACAATTAGTTAAAGAACAAGATTACCCCATG<br>TAAGGGAATTTTTTTATGTCCTAATTTGCCGTATGCACTTGTAAATAAATGTGTATTT<br>TAACGTATATGTGTATGTGTATTTGATGTATTGGTGTGTATGTCAACAAATCAATAAT<br>ATGATCACATAGGATAAGGTCAAGACCAATTGTTTAAGTTTTATTAGAATTCCGTGA<br>ATATGAAAAAAACAAAACAGGTCCTTTTGGATAAATCCTTAATAATGGGG	
KC608038	TAAGGCTATTCTTTAACAGACATCTGTTTGTTTCATTGAAAATGGCTGCCATGAATTC<br>TATCAAAGTTACAGTTAGAGCCAATGGACAGGATTTCACTCCGGTTTGTCCTAAAAT<br>GGAAGATGAAGTGGCTTACCCATCCGATTATTTGGATGCCAATGGATTACCAACCTT<br>CCAGCTCTACTATCATGATCTGTCAAAAAAGGATCTGCTAGATTTGATCTGGGGAG<br>AAGCATCAGAAGCAAGGCTGCCCAGCGAGCTGGTGACAGCGTATGTGTACAAGGT<br>GGTGAGTGAGTGGAAAGAGATGCTGGAGAGTGATTGGTCCTCTTTCCGGTTCCAGA<br>TTGGCAAAGCAGGAGAAGAGATAACCCCATTCAACCTGATTGGCATGACTGTGAAC<br>AATCAGAAGCTGGCTGATTACAAAAAAGCAGTTGCTCCTGAGGGGATAGATGAGG<br>TGGCAATGGTGATCTATCTCCTGGCACCATACCGGATAGTCGGGATTAAAAATGAA<br>GACTATCAGGATCGGGTGATCACCAACATCCAGAACCAGCTGGACAGTCTCGGA<br>GCAAAGAAGCTGCAGGTGAAGGCACTGAAGAATGTCACTACTCTTATTAACAGTGC<br>AAACTATCTGAGAATGGTGGCAGTGATTGATATGTTCTACTACCATTTCAAGAACA<br>GTCAAGAAAGAGCTGTTGTCAGAATTGCCACTCTGAGTTCCCGTCACAAGGACTGT<br>GCAGCTCTATCCACCCTGAACCACATTACCAGCTTCACCGGGAGGAGTTTCGTGCA<br>GGTGTTGGACTGGGTTTTCACAGATCAAGTCGCCAAGGAGATCGGCAGGATGATGC<br>GTGCGGGTCAAGAAATTGATCGTCCTGAATCTTATATGCCGTACCTGAAAGACCTG<br>GGGCTGAGCAGAAAACCCCCCTACTCTTCTTCAGCTAATCCCGGCACTCACTGCTG<br>GGCACAGATGGTGTGCGCCATGATGGGGTCCAAAAGATCCCAAAATGCAATCGCC<br>AGCACCGAAGAGAATCTCAGCAATCTGACAAGAAATGCTGAGATCATGGCCTATG<br>CCCTGGGAGTGGGAGCAGACCTGGTGAAGGGACTGATCATTGGAGATCAGAAGGA<br>AGGAGACTCAGGTGTCATCCAGGATGAGGAAGGAATGGATGAGCCCAACAACATG<br>GAGGCTCAAGATTGGCTGGAGTACATGGCCTCAAAGGGATTCAAGCTGACTCCAAA<br>TATGGAGCTGCAAGTCCGCCACATGTGTCTGCGAATCACCAATCCCAGAAAGGCGA<br>CTTTGGGGAGTTACCTGCGGGAGAGATACAGTTCATCCCTGTGAGTTGAAGCAGGA<br>ATGAGTCATTTTTAAAACCATTGTATTTCTATTTTAGTAGTCTCAAATAGTTAGTCAT<br>CCTTTGCTTAGATGTAATGCTTTAGTTATATGAAAAAAACTTTAACAGGGATAAACA<br>TAGTTTCTATAGCACTATTTTATCTCACGCATTTCAATTAATATGTTGTCATCTAACA<br>GAAACAAAAACAAATTTGACTTAGAGGGGATACAATTGCTGGCAAAAGGAGTCAA<br>AAATGCAGGAGAATCTATAGATAAAGCCACAGATGAGAAGCCTGAACATCTCTCT<br>GACGCATTCTCAAAGTATACAGAATATCTGTCCAATGAGAGCAAGGTGGAGGAGG<br>AGGATTTGCAATTCGAACAGGTGGACTATGGATTTCAAGAATCACCGGAGTCCAAT<br>AACCCTCTGCAAGAGAGCTGGATCGCAGAAGATGATCTGGAGCCAGATATGGGAA<br>AGTCCTGGGTAGAGTATCAAGCTAAGATGTCCTTTGATTACAGTGAGCAGGTGAAA<br>CCCACTGTGATGAGGGAGATCAACTGCTTGCTGAGTATGCTGGGTGGATTCGCTAA<br>ATTCCAAGATGGAAAGAAGGAATATCTTTTCTATCTTCCAGAGAAAAAAGAGTCAG<br>ACGAAAAAGGATCTGATAAGAAACAATGTCCTTTAAAGGTTGATGTCACCCCAAA<br>ACAGGACCAAAAGATCACTCCAACACCTGAGAAAGAACCAAAACATCTGCCCACA<br>TCAGCTGTCGGCAAAGAAATCACTGAAGAAGCAGCCGTGATGCAAGGATTCTGGA<br>TGGACGGGATGAGGCTGACAGAGAAGACATCAGGGAAGTATTGCCTGTTCTTCCCA<br>CAAAAAATGGGATGGTCTCAGGCTGAATGGATCAGCAAATCAGAAGACATTAATC<br>CCAGAACTCTGGCACATGACATCTTCAAATGGATGGTATCCAAATCTCCGAAACGG<br>GCCACATACTTGAGGAAATACATGGTAGAAGAGTGATCACAGATCTGAAAACCTTG<br>ACTATTTTTGATGCCTTAAATTGGCTTGACTCATACATATTGCTTATTACATGTCTCA<br>GTAGTTTATATGAAAAAAACGTTAACAGACGTCAATAACATTTGTTATTTACGCTAT<br>CATTGTGAGAATATATTCAAACGTCGAGTCAATTGAAGTTAAATGGCTCTATCTATA<br>TTTAAGAAGAAGGGGAAGAAGACAGAATCTCCTAAGATGTTTTTAATGGATGCGAA<br>TCAGATGACCCCGAGTGCTCCACCCCCTTACCAAGCAGAACCTGGTCCGTTCGACA<br>CTTGGGGGAATGAGGAGCTAGAAGAAGCTATGAAGGTGTGCTACTTAGTAGACAC<br>CTGCTTGTCTGTGACAACAAGAGAACCCATCCGATCAGTGGTTGATGCTTATATCAT<br>AGCGCAGGGAGTCTTAGATCATTACACCGGCCCGATCCTTACCCGTCCTTTTTACAT<br>AGCCTTGTTTTTGGGCGGGATACACGGGATGCAGGCAGGAGTCAAAGGAGCGAGA<br>AGCATCAGGTATGAGAGAGAACATCAGGGACCTCTGGTGTTCCCATATCACAGAAG<br>CAACCCGCTCGATTGGACTCCCCGGGCAATTGAGTTCCAGTACACCACTAGTCTGC<br>GGGGAAAACCGGTGGAGGTGAGCTTCCAGGCCCGTCTACAAGCCACCAGACAATT<br>CGGACCCGGTGTTGAGGTCTATCTGGATGGCCTGCAACGGATAGAGCGCCCTAGCA<br>ACGAATTGGTTCTCAAACAGTTCAGAGTCCCCCTGCTGATGCTGGAAAAAGGGAAA<br>TGGGTGTTCAATCTTGATTCTGAAGGTAGTTATAATTAAAAGAGTCCAACAATTAAA<br>TCATCTAAGCCACTCTTGTTAGAATCATATGGGCAAGTCTATCACTAAACATAGATG<br>GAATAGCATGTACACACAGAAATGCATCATATTATTGATTATTAAGTTATATGAAA<br>AAAATTAACAGTGATACACGATTGTTTCGAGATACTTATCACTGTAAAACTTAATTG<br>ATTCAATAAAAAGATGGATACTCTGATTAAAGTTTTGCTGATTATAGTCATTCTAAA<br>ATCTCTCCGTGCTCACATTGAATTTGTTCCACCTGATCTGAGCAAATGGAGAGAAAT<br>CAGCATAGGGCACTTGGATTGTCCAATCTATGGAGATCTATCCAATCAAGCAACTA<br>GACCGGTCTCTGTGAAATACAGCAGTGTGCAATGGGGCTTAAAAAATAACATTGAT<br>GGGTACCTTTGTATATCTGCCAAATGGTCAGTCACTTGTGATTATAGGTGGTATG<br>GCTCAAAATACATTTCAACTTCAATTGAATATGTTCCAACCAAAGAATCGGAATGC<br>AGAGATGCCATCAAATCATCAAAAAAATGGAGAATTGGTCAGTCCCCACTTTATGCC<br>CGAAAATTGCGGATGGAACAATGTTCTAACGGAGAGTGTGACATACACAACTGTAT<br>CATCTCACGAGGTAAAACTAGATCCATACCAGATGACTTTCGTGGATTCACTCTTTC<br>CGGGCGGTAAATGCTCCTCTTCAGTGTGTAACACTATCTATCATCAAGGAGTCTG<br>GATAAACCTGAATGACAACTTAGGGTTTTGCAGAGAACCAGTCGATCATCAAGGTC	KC608038.1 Eel virus American isolate J6B4, partial genome<br><br>[54]

	AGTTGTACATGGCTGGTCTTGTTGGAGCTCAAGGAAACATTGTGAAAGAAGTGTGG<br>AATCTCAGATCTGTGTTCAAGCCAGAAATTGGCAGATCTAAGCATTTAACCGGATC<br>CTGTTGGATGACATACTGTGGGCAAAGAGGACTGAGATTCTCAGACGGAGAATGG<br>GCAGGTTTCCAAATTCCAGAGATCTCAGCACTGAAACAGGTTCTGCTAGGACTTCCT<br>GAGTGCAAAGATGATGTGCTGGTTCATGCTCATGACACGAACACTGAGTTGAGGGA<br>AATTTTGGAACACATGGACGAGTCAGCATTGAATGCCATCTGCCAACAAGAAGTCC<br>GCAGGGCAAAAGAGAGAGGAGTGGTTTCTGATTGGCTATTGAGTATGATGACACCA<br>TTCACAGAGGGGTTGGGGCCAGTTTATCGGCTGAACAAAGGAAAACTAGAAGCAT<br>CAATGGGATATTATAGAAAGGTATATATAGACTCTAGCAACGCCCCTCAGGCCTTC<br>GGACAGACAGAAGATAAAGAATCTGTTGGATGGTCGGATCTTGTACCAAAAGATG<br>CAGATGGAGCTATATCATCGATGTACAATGGGAATGTCGTCATCAATAATCAGATA<br>AAATGGGCTAAAAATGCACTAGGATCTCATATATTAGATGAGATTTCTGCCTTAGA<br>ATTTGAAACCCCCGTTGTGCATCATCCTCATTTGACAATCTTGTCAGTAAATCATAG<br>TGATCTAGTGAGTTCCACACATCCAAATGGGCAAGGAGTTAATCTGATTGAGAGTG<br>TATCTCACTGGGCCGGTGGGTTGTGGGCATCCATAGGATCTGGGTTGATGATCTTGG<br>TTCTAGTGGCTTTAGTCGGATTTTGTACCATCAAGGTATGCTTAGCTTATGTTCCATC<br>GATTTGGGCACAAAAAAATCGGAATGGTAAGAGAAGAGGAACAACATCCCAACG<br>ATCAACAGAACAGGAGATGTTCGAGCTATCAGCTGTGTAGGTACAAGTGATACTTC<br>AGGTTAAATCGGAACTCTCATTTCTGTGTAATCAGATCTATATATTAAAAGACCTA<br>CTTAAAATATTGCATGTGTTACTGATGTCAGCGTAATAAGCTGAATCATAATTTACT<br>TCTCATGACAATTTTGTTAACCTACATGTTTTCATCTTATTACATTTTATTCTTATTTTT<br>ATTTTTATGAATATATATGAAAAAAACTATTCAACAGTCATCATGTATGACGAAGAT<br>CATTCAAGAGGATATGAATCCGATGACCATTATGACCTGCCTGAATGGCTGGAAGA<br>GGATATACAATCAGGAAATCCACTCAATCAAAAAGACTATAGCTTAAATTCTCCTC<br>TCATTGTAGATCTCACAGAAGCGCTGGTAAAATATCTAAATCAAGGTAATATAGAG<br>AGACGATTCCTCAGACATCAAGACAGATTTGAGAACATACAAAGTGAAATCCGCA<br>ACATTGCATGGAAGAATCCATCGAGCTCAAATCACAGATGGTGGGGGAAGTGGGC<br>TCAACAAACAGAAAAGAGTCCAGAGTTTGTAAGGCTGTTGTCTGATGTTAACCAGG<br>ACATAGAAGAGACTAGTGACGTCTTGATGTCATTCTTGAAAGGATGGATCCAAGAC<br>ACAACCTCTGTTCCAACAAAATTAAATTGGACATCTACTCAATTGAGCTATGGATCC<br>AAATTCTTCTTCATGCACAAATTGATCCTGTTCATGAATGCGCAGTCAGACGAAGAG<br>AGAGTCATTCTCCAAGGGCACATCAAAGTCTCGGAAACAAAAAAAAACTGGGGTTT<br>ACAAAGGCTCTCATTCCAGCTTAGGAGACTTTGTTCTGACTTCTGAATTCTTACTACT<br>AGAACGACACAGAGTGATACTAGACCGTTCTTTCTTGTTGATGGTCAAAGATACTCT<br>AGTGGGGAGATTTCAAACACTCGCTAGCTTCATGAATAGGGAAGATCAGAAGTATC<br>CAGAAGATGTGATAGAAAAAGTAGAGACCCTCTATTCCTTAGGAGACCAATTAGTA<br>GAGGATCTAGGGGATGAAGCATATTCAGGTATAAAATTGCTGGAACCAGCCTGTAA<br>CCTCCGACTCGCAGAATTGGCTAGAGAATTTAGGCCTTTGATTCCCGAATTTCCACA<br>TTTTAGAAACCATGTGGAAACAGCAATAGCAGAGGAGTCTGCCTTCAGTCCTGGAA<br>TCACAGAATTCTTCAACCATGTAAATAAGGAGACAAACGTGGAAATCATTTTGGCC<br>TACTTCAGTTCTTTCCGACATTGGGGACATCCGTACATTGATTATTTTCAAGGATTGA<br>TCAAATTGAACAAGCAAGTCACTCTGGAGAAGGACATCGATACAGAGTACGCAAA<br>TGCGCTGGCTAGTGATTTAGCATACATGATTCTTCGAGGACATTTTAACACTAAGAG<br>AGTCTGGGCGGTCGACAAAGCACTTGTATCAAAGCAACATCCGCTGAGTGAGCATA<br>TTCTCAATGCCACTTGGCCGACTCCGAAACAGATTGATGACTTCGGAGATCATTGGC<br>ATGAATTGCCTCTGATCAAAATTTATGACATCCCAGATTTAATTGATCCATCTGTCAT<br>CTACTCTGATAAGAGTCATTCAATGGGTAGAGAAGAAGTGTTAAAACATGTTCAAA<br>GGAATCCAACACAAGCAATCCCTACAAAAAAGGTCTTGGAGACCCTCCTACAAAA<br>GCCTGCCACAAATTGGCCAGAGTTCTTGTCCTCTATTGAAAAAGATGGCCTACCGA<br>AAGACAGCTTGATTATAGGACTGAAAGGAAAAGAGAGGGAACTTAAAAAAGCCG<br>GAAGGTTCTTTTCCCTAATGTCTTGGGAATTAAGAGAGTATTTTGTGATAACCGAGT<br>ACCTGATCAAAACCCATTACGTCCCTCTCTTTAAAGGACTGACTATGGCCGACGAC<br>ATGACAGAAGTCGTCAAAAAGATGTTGGAAAGAAGTCAAGGACAGGGAGAGGAT<br>GATTATGAGCATGTCAGCATTGCCAATCATATTGATTACGAAAAATGGAACAACCA<br>TCAAAGGAAAGAGTCCAACGGTCCGGTGTTTCGAGTCATGGGACAATTTTTGGGGT<br>ACCCTAGTTTGATAGAAAAGACCCATGACTTTTTCGAGCAAAGCTTGATCTACTATA<br>ATGGCAGACCTGACTTGATGCAGACAGACGGGAATGAGTTGCAAAACCGAACTGA<br>AGCGTTGGTGTGTTGGAACGGTCAGAAGGGAGGATTGGAAGGACTCAGGCAAAAA<br>GGATGGAGCATTCTCAATCTGTTAGTCATCAAAAGAGAATCTAAAATCAGAAACAC<br>TAAAGTGCAAACTTTAGCTCAAGGAGACAATCAGGTAGTGTGCACCCAATACCGCA<br>TCATGCCAACCAGATCGACACTTGAATTGCAGGCAGAACTTGAAAAAGTTAAGAA<br>GAACAATCAAGTTATTATGGATGCTATTGAGACAGGGACTAACAAATTGGGATTAC<br>TGATCAACAACGATGAGACCATTCAATCTGCTGATTTCTTAACATATGGTAAGGTTC<br>CAATATTCCGGGGTAACATTCGATGCTTGGAAACTAAGAGATGGTCTAGAGTGACT<br>TGTGTCACCAATGACCAATTGCCATCTCTGTCCAATGTCATGTCATCTGTATCGACA<br>AACAGTCTGACTGTCTCGCACTTTGATGTCAGTCCCATAGAATCCATGAGACAATAC<br>CTGTTCTTTGGAAATTTTGCTCGGAGATTGGTGGAATTTCACAATCCTGCGATGAGA<br>GTGCCAATTAGTCTAGGGGACCTGGATTCTAAACAAAGGTCTGTTTATCTGAATGCA<br>GTGCTTTTCTTGGACCCCTCTCTGGGTGGTGTGTCTGGCATGTCATTATCCAGATTTTT<br>GACTAGGATGTTTCCTGATCCCATCACAGAAGGATTATCCTTTTGGAAAATTGTTTA<br>TGAACACACCACTTCTAAAAACACTCAACTGCTCTGCAGAATCGCTGGAAGCCCCG<br>AATTGGCCAGACGACAAAATAATCTAGACAAGTTGATAGAGAATCCAACAGCTTT<br>GAATCTTAGCAAAGAAACTTCCGCCCTAAGTGTCATTAAGAAAGAAGTCAGGTCAC<br>GCTTGTACAAGGATTGCGACAAATTCAAGAACAAATTGATTGCAGATGCAATCGGG<br>ATTGCTAGAGATGAAGAAGCTCACCTGGAATTGTTCTTAATGTCAATCCGACCA<br>TTATTTCCAAGATTTTTGGCAGAGTTCAAAGCAGCCACATTTGTTGGCATCACAGAA<br>TCTCTCATTTCTTTATTCCAAAATTCTAAAACCATTCGCAACATCTTTAGAAAGAAAT<br>ATGCAAAAGAATTAGAACTTAGAGTAGTGCAGTGTGAGTACAGATCCATCAATCTG<br>ATGTTGAGTCTCGCAGATCGATCCCACCTGGATGAAATGTGGACATGTTCAGCATCC<br>AAAGCGGATGAACTCCGAACACTATCTTGGGGGACTACAATAATTGGAACAACTGT	

	CCCTCATCCTTTAGAGATGATCAATCATGCTCATGTAGGACAAAGATGTGATTCGTT<br>GGAAACTCTGGACTACATAAATGTTACTGTGGTTCAAGATCTAACAGACTGTCTAA<br>CTAGCAAAGGGAAGTTACCAGCGTACTTAGGATCTAAAACATCAGAGACTACAAG<br>CATCTTGCAGCCATGGGAAAAAGAGACAAAAATCCCAGTGATTCGCAGGGCAGCC<br>AAATTGCGAGCTGCAATCACCTGGTTTGTAGAGCCCGACTCTCTTTTAGCACAAAGC<br>ATCCTGAACAACATTGAAAGTCTAACTGGAGAAGATTGGTCCGCTTCAATATCTGG<br>ATTCAAGAGGACAGGATCTGCACTACATCGCTTCACTAGTGCTAGAGTGAGTGCAG<br>GAGGCTTCTCAGCTCAAAGCCCAGCTAGATTGACCAGGATGATGGCAACAACAGA<br>TACCTTTAGAGAAATTGGATCTGACAATTACGACTTTATGTTCCAATCATTACTACTT<br>TTTGCCCAAATGACTACGGGGGAAATCTACAAAAGGAGTCCAGCGACTAATTTCCA<br>CTTTCATCTAAGTTGCCACCAATGTCTTCGTAAGATTGAAGAGCCTACACTAAACTC<br>TGATTTTGCTTACAATCCTATTCAGAGATCGGACATCTTGGATAAGTGGAAACCTCA<br>GACCACAGATTGGTCGTCAGAAAGAAAGGCTCCAGAGATAGAGGAAGGGAATTGG<br>GACAGATTGACACATCAGGAACAGAGTTTTCAGATAGGGAAATCTATCGGGTTTCT<br>ATTTGGAGATTTAACAATGACCAAGAATTCACATGCTCAAGACTCTTCTATTTTCCC<br>ACTGTCAATTCAATACAAGATCACAGCCGCGGAGTTCCTAGAAGGAATTCTAGATG<br>GGATAGTCAAGGCTTCTGCCCTGTCTACTATCCATCGGAGAAACTTCGATCATCACA<br>GTAAGTACAAGTCCACTGTTTCAGGAACTGTTGATTACTTAATCGAGTTGATTTCAG<br>AATCAGCAGGATTCACAAATTTGACCAGGAATGGTCCACTGAAGGCTTGCTTACTG<br>ACCATCCCACACAAGATACCTCCATCTTATCCTCTCAGCCAATCTGATCTTGGTGCT<br>ATGTCAAGAAACTACCTCAGACTGTTGCATCGGAGAATGTCTGCTGGTACCTACAA<br>GACTAGGTGGCCGACAAATTGGATATTTTCTGACATGATGAGCCCTAACATAATCT<br>ATCCATTTGTGATCAGTGTCTCTTGTGTTGGACTGGCTTACTCATCATCTTGGACAAA<br>GAAATCTGCTGATAAGTTGAGGGGATTAAGAGGAGTGGCTGAGTTGATTCGATCAT<br>CAGATGATGTGCAACTGCCTGTTGGGAAGCTATTCAAAACAGTGAACCAAGAAATT<br>AGACATGCCATAAAGCATCATGCATCGGACGATGCTGAAATTCCTGAAAGCCACAT<br>CCAAGAGAGCTGGAAGAAAGAACTCATAGTCAACATCAACGTGCAGCCAATTGAC<br>TACAGTAGGACAGCGACAGTAAAGTCTTTAGATCGGCCTGCACAAATTCGAGATCC<br>TTTGATATCTGGACTAAGGACAGCACAATTGGCCACTGGATCCCACTACAAATTAC<br>GATCTATACTGGTGCAAAACCGAATTCAAGTTACTGATGCTTTGTGTGGAGGGGAT<br>GGATCTGGTGGAATTGGGGCTTCTGTCATGAGACAATACCCGATTACACAGGTGAT<br>TTACAACAGTCTGTTCGAGATTCAAGACCTGGACATGCGGGGGAGTGCACCTGGAC<br>CACCATCTGCTATTGCCGCAATGGGGAGCATGAGCATGAGGTGTGTCAATCGAGAT<br>TCTGCATGGAAAAATCCATCCGATCTGAGCCACACTTCTACCTGGGAATATTTCCAG<br>TCGTTGATGACCCAACATCAGTTGCGGTGCAATCTGTGGACATTTGACATGGAAGTT<br>CGCAGCCATGACATTTCAGACGCTATAGAAAAGCAAATAGTCGCCAATCTTCATTT<br>GCTACCAAAAGGTGGGACCATCATATACAAGACATATCTAACGAAATTGTCTGACA<br>TGGAGACAACAATCCTAGACAGATTGGGTGGATTCTTTAAAAGAGTCAGTCTTGTG<br>TCCACTGATGCTACATCAAGTCATAGTTCCGAAGTCTATGCTGTATTCCAAAACAAG<br>TTGGATAAGAGACAATTGGAAATCCACCCCAATTGGTCTTCCTGCAATCTAGGTTCC<br>GACATACACCCATGTTGGAAAAGTGAAGAGGAAGAGTTTGAAAGGGCAAGAAGAT<br>TTTTCCACATGAGAAGACAACAAGGGGTGCCGAATCGATTGAGACCCACTCTGGAT<br>TCTGAAATCCAAGTCTTGAGTGTCTCTGCTGGAGTGGAAAATGGGGTATCAATGAC<br>ATTAGCTATGGATGTAAGCAATCAGGTCTCAGATCCAACCACTGGAGCATTTCTTTG<br>GCTCCTAGTAACCTTGCAACATATATGCCCAATAGGCCCATCATTCAAGATCCCATC<br>TAGTACTGCAGTCGAATCTTATCTGGCCATATTGATTGGATTCAGTAGTATTTATCAG<br>CTTCAGACGGGCAATGGCAAAGCTTATGCCAACATCAAGACCTGCTTGTCTCAATC<br>TGCTCCATTCTTCTGCAACTCCACTGGATGGAGTTGCGTTAAGGGACTTGACAAGTC<br>TATGCGGATGGACAGGAAACTGGCTTTAGTGGGTAGTGTAATACGGGCATGGTCGA<br>AGTGGAATCTGACTCAGTCAATCAACTTTCACAAGTTAGATGGCATGATGAGACAT<br>TATCTGCCGAAGGGAGCATTGAGGCAGATTGGCTGCAAAACCGGAATCTGGGATTA<br>CATAAATGGAGCAGTTAAAGGAGTGCGAAGTGCTGCCAATCAAGACTGTCCAAAA<br>GAGTCAGCAGCCGCTTGGAGAGATTAATATATAAGATTGGGTCTATCATCCAACTC<br>ATTATCCAATAGGTAACAATTAATCAAAAATAGGACTCCATTCCAGATAATTTTTG<br>TCCCAATTTGCTGTATGTACTTGTAAATAAATGTGTATCTTAATATATGTGTGTATAT<br>GTATTTGATGTATTGGTGTATATGTTAACAAATCACGATACGATAAAATAGGATAA<br>AGTCAAGATAAATTTTTCAAGCTTTATTATAATTCCGTAAATATGAAAAAAACAAA<br>ACGGGTCCTTTTGGATAAATCCTTAATAATGGGG	
KJ598499	AAATGGCTGCCATGAACTCTATCAAAGTTACAGTTAGGGCCAATGGACAAGATTTC<br>ACTCCAGTTTGTCCTAAAATGGAGGATGAGGTGGCTTACCCATCTGATTATTTAGAT<br>GCTAATGGATTACCAACCTTCCAGCTCTATTATCATGATCTGTCAAAAAAGGATCTG<br>CTAGACCTGATCTGGGGAGAAGCATCAGAGGCAAGGCTGCCTAGTGAGCTGGTGA<br>CCGCGTACGTGTACAAGGTGGTGAGTGAGTGGAAAGAGACGCTGGAGAGTGATTG<br>GTCATCTTTCCGATTCCAGATTGGCAAAGCGGGAGAGAAGATAACCCCATTCAACC<br>TGATTGGCACGACTGTGAATAGTCAGAAACTAGCTGATTACAAAAAAGCAGTCGCT<br>CCTGAGGGGATAGATGAGGTGGCAATGGTGATCTATCTCTTGGCACCATACCGGAT<br>AGTCGGAATTAAAAACGAGGACTACCAAGATCGGGTGATCACCAACATCCAGAAC<br>CAGCTGGACAGTCTCGGCGCAAAGAAGCTGCAGGTGAAGGCACTGAAAAATGTCA<br>CTACTCTCATCAACAGTGCCAACTATCTGAAAATGGTGGCAGTGATTGACATGTTCT<br>ACTACCATTTCAAGAACAGTCAAGAAAGAGCTGTTGTCAGGATTGCCACTCTGAGT<br>TCCCGTCACAAAGACTGTGCAGCTCTATCCACCCTCAATCACATCACCAGCTTCACT<br>GGGAGGAGTTTTGTGCAGGTGTTGGATTGGGTTTTCACAGATCAAGTTGCCAAGGA<br>GATCGGCAGGATGATGCGTGCGGGTCAAGAAATTGATCGTCCTGAATCCTACATGC<br>CGTACCTGAAGGACTTGGGACTGAGCAGAAAATCCCCCTACTCCTCCTCAGCAAAT<br>CCTGGCACTCACTGCTGGGCACAGATGGTATGTGCCATGATGGGATCCAAAAGATC<br>TCAAAACGCAATCGCCAGCACTGAAGAGAATCTCAGCAATCTGACAAGAAATGCT<br>GAGATTATGGCCTATGCCCTGGGAGTGGGAGCAGACCTGGTGAAGGGACTGATCAT<br>TGGAGATCAGAAGGAAGGAGACTCCGGTGTCATCCAAGATGAGGAAGGAATGGAG<br>GAGCCCAACAACATGGAGGCCCAAGATTGGCTGGAGTACATGGCCTCAAAGGGAT<br>TCAAGCTGACTCCAAACATGGAGCTGCAAGTCCGCCACATGTGCCTGCGAATCACC	KJ598499.1 Eel virus European X isolate ToBo nucleoprotein (N), phosphoprotein (P), putative coat protein (C), matrix protein (M), and glycoprotein (G) genes, complete cds<br><br>[49]

	AATCCCAGAAAAGCCACTCTGGGGAGTTACCTGCGGGAGAGATACAGTTCATCCCT<br>GTGAGTTGAAGCAGAAATGAGTCACCCCCAGAACTATTACATTTTAATCCTAATAG<br>CCTTAAATAGTTAGTCATCTTGTGTTTAGATATTATGCCCTAGTGATATGAAAAAAA<br>CTTTAACAGGGATAAACATAGTTTCTACGGCTCTATTTTATCTCTCACATCCTAATTA<br>ACATGTTGTCATCTAACAAAAACAAAAACAAATTCGACTTAGAGGGGATACAATTG<br>CTGGCAAAAGGAGTCAAAAATGCAGGAGAATCCATAGATAAAGCCACAGATGAG<br>AAGCCTGAACATCTCTCTGACGCATTCTCAAAATACACAGAATATTTGTCCAATGA<br>GAGCAAGGGGGAGGAGGAGGATTTCCAATTCGAACAGGTGGACTATGGATTTCAA<br>GAGTCACCTGAATCCAATAACCCTCTGCAAGAGAGCTGGATAGCGAAAGATGACC<br>TGGAACCAGATATGGGAAAGTCCTGGGTAGAGTATCAAGCTAAGATGTCCTTTGAT<br>TACAGTGAGCAGGTGAAACCCACTGTGATGAGGGAGATCAACGGCTTGCTGGGTAT<br>GCTGGGTGGATTTGGTAAATTCCAAGATGGAAAGAAAGAATATCTCTTCTATCTTCC<br>AGAGAAGACAGAGTCAGAAGAGAAAAGGTCTGATAAGAAACAATGTCCTTTCAAG<br>GTTGATGTCACCCCAAAACAAGACCCAAAAATCACTCCCACACCTGAGAAAGAGC<br>CAAAACATCTGCCCACATCAGCTGCCAGCAGAGAAATCACCGAAGAAGCAGCCGT<br>GATGCAAGGATTCTGGATGGATGGAATGAGGCTGACAGAGAAGACATCAGGGAAG<br>TATTGCCTGTTCTTCCCACAAAAAATGGGATGGTCTCAAGCCGAATGGATCAGCAA<br>ATCAGAAGACATTAATCCCCGAACTCTGGCACATGACATCTTCAAATGGATGGTAT<br>CCAAATCTCCGAAACGGGCCACATACTTGAGGAAATACATGGTAGAAGAGTAACC<br>ACAGGTCTGAATCTCTTGACTATTTTTGATGCCTTAAATTGGCTTGACGTACACGTAT<br>TACTTATTACATATCTCAGTTATTTATATGAAAAAAATGTTAACAGACGTCAATAAC<br>ATTTGTTATATGCGCTATCATCGTGAGACTATATTCAAACGTCAAATCAATTGAAGT<br>TAAATGGCTCTATCTATATTTAAAAAGAAGGGGAAGAAGACAGAATCTCCCAAGAT<br>GTTTCTAATGGATGCAAACCAGATGACCCCGAGTGCTCCACCCCCTTACCAGGCAG<br>AACCTGGTCCGTTCGACACTTGGGGGAATGAGGAACTAGAAGAAGCTATGAAAGT<br>GTGCTACTTAGTAGACACCTGCTTGTCTGTGACAACAAGAGAACCTATTCGATCAGT<br>GGTGGATGCCTATATCATAGCACAAGGAGTCTTAGATCATTATACCGGCCCGATTCT<br>TACCCGTCCGTTTTACATTGCCTTGTTCTTGGGCGGGATCCACGGGATGCAGGCGGG<br>AGTTAAAGGAGCGAGAAGCATCCGGTATGAAAGAGAACATCATGGACCTCTGGTG<br>TTCCCATATCACAGAAGCAACCCGCTCGATTGGACTCCTCGGGCTATTGAATTCCAG<br>TACACCACTAGTCTGCGGGGGAAGCCGGTAGAGGTGAGCTTCCAGGCCCGTCTGCA<br>AGCTACCAGACAATTTGGACCTGGCGTAGAGGTTTATCTGGATGGCCTGCAAAGGA<br>TAGAACGCCCTAGCAACGAACTAGTTCTAAAGCAATTCAGAGTCCCCTTGCTGATG<br>CTGGAGAAAGGGAAATGGGTGTTCAATCTTGATTCTGAGGGCAGTTACAATTGAAA<br>GGGTTCAACAATCACATCATCTGAGCCACGTTTGCTATAATTATGCGATCAAGTTTA<br>CCGCCACAAAAGAGTTGAGTAGCATGTAGACACATATAAATGCATCATATTATTAA<br>TTAATTAATTGTTAAGTTATATGAAAAAAACTAACAGTGATACACCATTGTTTTGAG<br>ACATTTGTCACTGTGAAATTCAGTTGGTTCAATAGAGACATGGATACCCTGATTAA<br>AATTCTGCTGATCATAGTCATTCTAAAGTCTCTCCATGCTCACATCGAATTTGTGCCA<br>CATGATCTGAGTAAATGGAGAGACATCAGCATAGAGCATCTGGATTGTCCAATCTA<br>TGGAGATCTGTCTAATCAAGCAACTAGAACAATCCCTGTGAAATACAGCAGTGTTC<br>AATGGGGCCTAAAAAATGACATTGATGGATACCTTTGTATATCTGCCAAATGGTCG<br>GTCACTTGTGATTATAGGTGGTATGGCTCCAAATACATTTCAACCTCAATTGAA<br>TATGTTCCAACCAAAGAATCAGAATGCAGAGATGCCATCAAGTCATCAAAAAATG<br>GAGAGTTAGTTAGTCCCCATTTCATGCCCGAAAATTGTGGATGGAACAATGTGTTAA<br>CGGAGAGTGTGACATACACAACTGTATCATCTCACGAGGTAAAATTAGATCCATAC<br>CAGATGACTTTCGTGGATTCACTCTTTCCGGGCGGTAAATGCTCCTCTTCAGTGTGTA<br>GCACCATTTATCATCAAGGAGTATGGATAAACCCGAGTGACAACTTAGGGTTTT<br>GCAAAGATCCGGTCGATCATCAAGGTCAGTTGTACATGGCTGGTCTTGTTGGAGCTC<br>ACGGAGATATTGTGAAGGAAGTTTGGAATCTCAGATCTGTGTTCAAGCCAGAAATT<br>GGTAGATCCAAACATTTGACTGGATCCTGTTGGATGACATACTGTGATCAAAGAGG<br>GCTGAGATTTTCAGATGGAGAATGGGCAGGTTTCCAAATTCCAGAGATCTCAGCAC<br>TGAAACAAGTTCTGCTAGGACTTCCTGAGTGCAATGATGATGTGCTGGTTCATGC<br>TCATGACACGAACACTGAGTTGAGGGAATTTTGGGAACACATGGACGAGTCAGCAT<br>TGAATGCCATCTGCCAACAAGAAGTCCGCAGGGCAAAAGAGAGAGGAGTGGTTTC<br>TGATTGGCTATTGAGTATGATGACACCATTCACAGAGGGGTTGGGGCCAGTTTATCG<br>GCTGAACAAAGGAAAACTAGAAGCATCAATGGGATATTATAGAAAGGTATATATA<br>GACTCTAGCAACGCCCCTCAGGCGTTCGGACAGACAGAAGATAAAGAGTCTGTTGG<br>ATGGTCAGATCTTGTGCCAAAAGATGCAGATGGAGCTATATCATCGATGTACAATG<br>GGAATGTCGTCATCAATAATCAGATAAAATGGGCTAAAAATGCACTAGGATCTCAT<br>ATATTAGATGAGATTTCTGCCTTAGAATTTGAAACCCCCGTTGTGCATCACCCTCATT<br>TGACAATCTTGTCGGTAAATCACAGCGATCTAGTGAGTTCCACACATCCAAATGGG<br>CAAGGAGTTAATCTGATTGAGAGTGTATCTCACTGGGCCGGTGGGTTGTGGGCAT<br>CCATAGGATCTGGGTTGATGATCTTGGTTCTAGTGGCTTTAGTCGGATTTTGTACCAT<br>CAAGGTATGCTTAGCTTATGTTCCATCTATTTGGGCACAAAAAAATCGGAATGGTA<br>AGAGAAGAGGAACAACATCCCAACGATCAACAGAACAGGAGATGTTCGAGCTATC<br>AGCTGTGTAGGTACAAGTGATACTTCAGGTTAAATCGGAACTCTCATTTCTGTGTAA<br>TCAGATCTATATATTAAAAGACCCACTTAAAATATTGCATGTGTTACTGATATCAGC<br>GTAATAAGCTGAATCATACTTTACTTCTCATGACAATTTTGTTAACCTACATGTTTTC<br>ATCTTATTATATTTTATWCGTATTTTTATTTTTATGAATATATATGAAAAAAACTATT<br>CAACAGTCATCATGTATGACGAAGATCAT	
